# Supplementary material for: The dimorphic diaspore model Aethionema arabicum (Brassicaceae): Distinct molecular and morphological control of responses to parental and germination temperatures
Source: Plant Cell. 2024 Mar 21;36(7):2465–90. doi: 10.1093/plcell/koae085 (PMC11218780; doi:10.1093/plcell/koae085)
Supplement: koae085_Supplementary_Data [file koae085_supplementary_data.zip › Chandler2024_Supplemental Data.pdf]

A ***Aethionema arabicum* plant growth (glasshouse)**

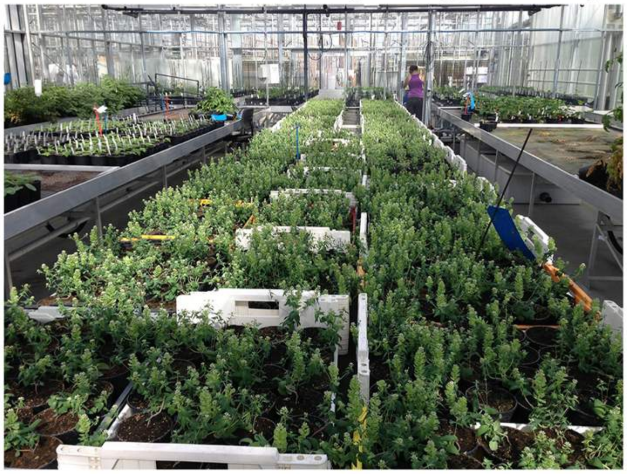

Glasshouse conditions: 16h light, 20ºC for vegetative growth of all plants

↓

Transfer to distinct temperature regimes during flowering/reproductive growth:

Day/night cycle: 16h / 8h
Light intensity: 170 µmol • s^{-1} • m^{-2}
Relative humidity: 65%
Temperature at night: 20ºC

Distinct day temperatures (PT, parental temperature during reproduction):
Set 1 (1000 plants): parental temp. 20ºC
Set 2 (1000 plants): parental temp. 25ºC

↓↓

Harvest of fruits and seeds followed by germination experiments and sampling

B **Sampling scheme during seed and fruit imbibition for transcriptome and hormone analyses**

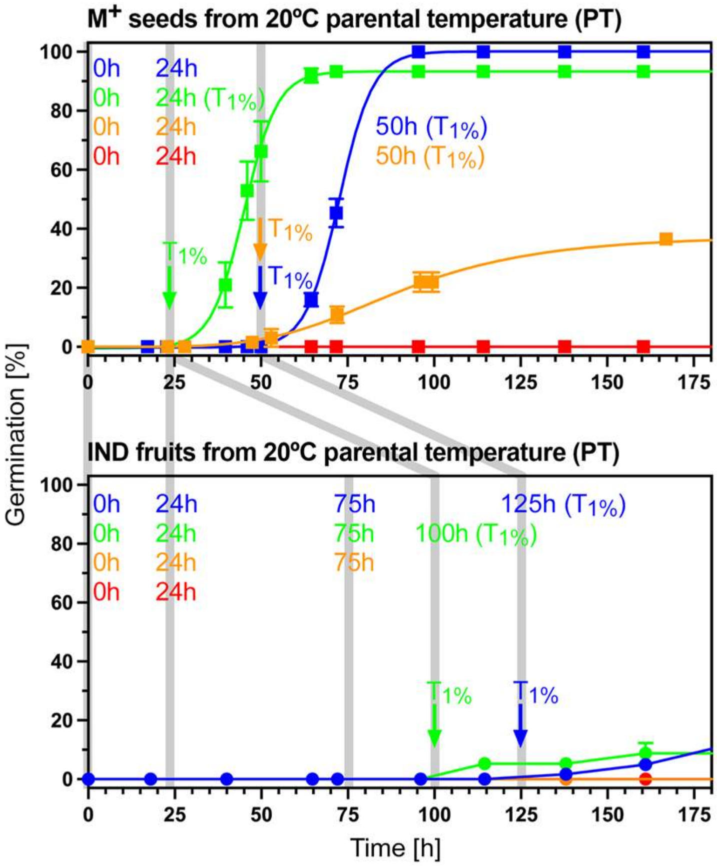

M^+ seed: 9ºC, 14ºC, 20ºC, 24ºC

M^+ seed $T_{1\%}$ times:

ºC	PT20	PT25
9	50h	35h
14	24h	24h
20	50h	no germ.
24	no germination	

Physiological times $T_{1\%}$ ↓

Sampling times (h) for $20M^+$ seeds as indicated in scheme; comparisons of physical and physiological times possible (grey lines).

IND fruit: 9ºC, 14ºC, 20ºC, 24ºC

IND fruit $T_{1\%}$ times:

ºC	PT20	PT25
9	125h	75h
14	100h	75h
20	no germination	
24	no germination	

Sampling times (h) for 20IND fruits as indicated in scheme; note that IND fruits were separated into M^- seeds and pericarp (fruit coat); transcriptomes were conducted with M^- seeds from imbibed IND fruits and for comparison with imbibed M^- seeds; hormones were also analysed in pericarps.

Supplemental Figure S1 continued next page…

C

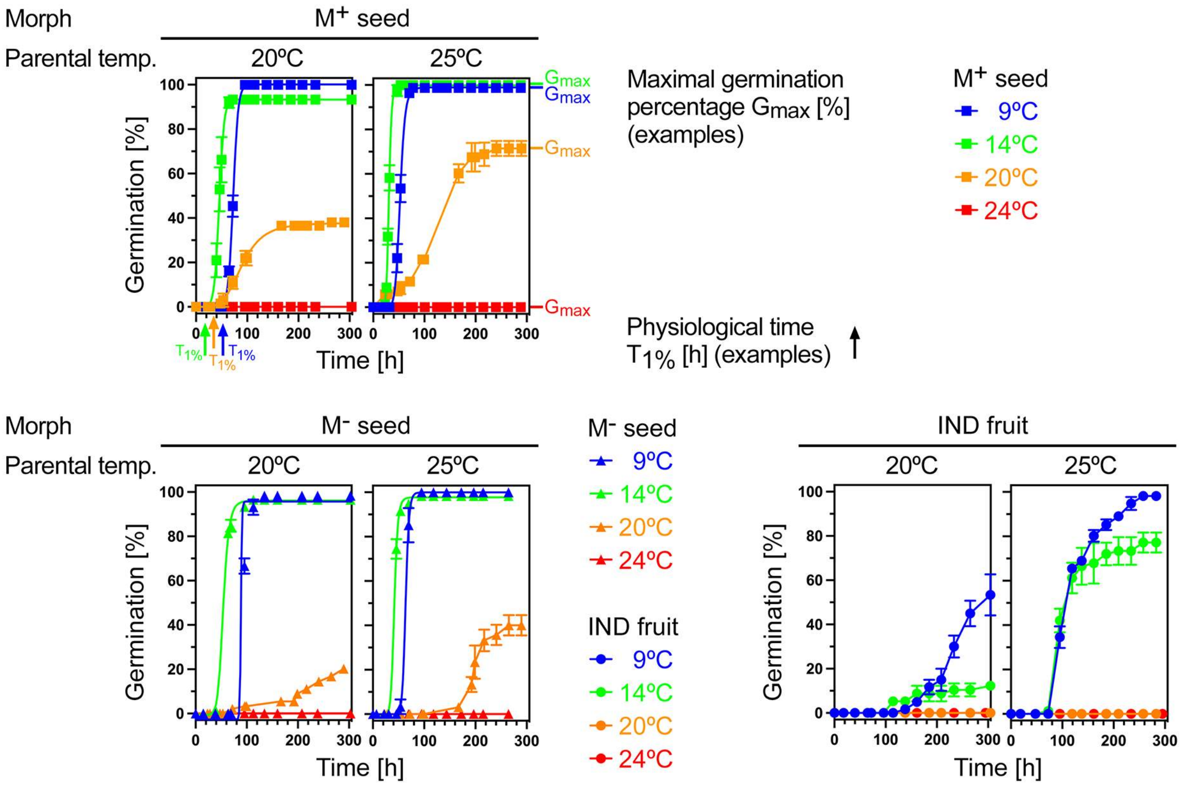

Supplemental Figure S1. Large-scale *Aethionema arabicum* diaspore production experiment, example germination curves and sampling scheme for the molecular analyses (Supports Figure 1). A, Plant growth with two distinct maternal temperatures during reproduction. B, Sampling scheme for the transcriptome and hormone analyses during seed and fruit imbibition. A combination of dry (0 h), physical (e.g. 24 h) and physiological ($T_{1\%}$) timepoints was used; $T_{1\%}$ represents the population's onset of germination completion and is therefore specific for each morph and imbibition temperature. C, Germination curves and maximal germination percentages (G_{max}). for *Ae. arabicum* M^+ seeds, IND fruits and bare M^- seeds (with IND pericarp manually removed) at different imbibition temperatures and as affected by distinct maternal temperature environments. Mean ± SEM values of 3 replicates each with 20 seeds or fruits are presented.

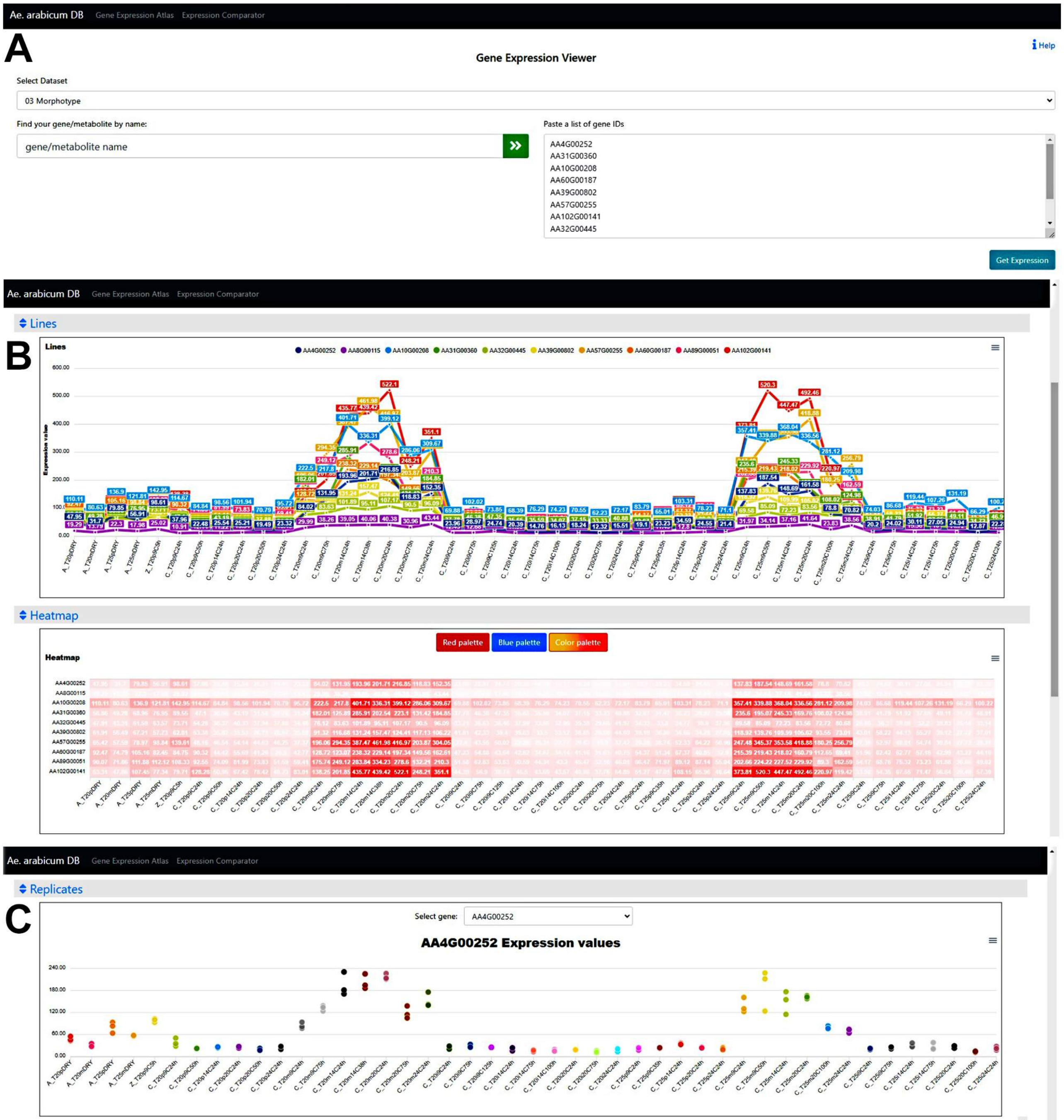

Average values

Copy | CSV | Excel | PDF | Print | Column visibility

Search:

	A_T20pDRY	A_T20mDRY	A_T25pDRY	A_T25mDRY	Z_T20p9C5h	C_T20p9C24h	C_T20p9C50h	C_T20p14C24h	C_T20p20C24h	C_T20p20C50h	C_T20p24C24h	C_T20m9C24h	C_T20m9C75h	C_T20m14C24h
AA4G00252	47.95	31.70	79.85	56.91	98.61	37.96	22.48	25.54	25.21	19.49	23.32	84.02	131.95	193.96
AA8G00115	19.29	13.79	22.30	17.98	25.02	10.91	12.14	11.22	13.34	13.11	13.43	29.99	38.26	39.05
AA10G00208	110.11	80.63	136.90	121.81	142.95	114.67	84.84	98.56	101.94	70.79	95.72	222.50	217.80	401.71
AA31G00360	56.86	49.28	68.96	76.95	89.55	47.10	30.66	43.19	31.58	21.75	31.24	182.01	125.89	285.91
AA32G00445	47.91	43.31	61.59	63.57	73.71	54.28	38.37	40.33	37.94	37.88	34.46	76.12	83.63	101.89
AA39G00802	61.91	56.49	67.21	57.73	62.81	53.38	35.82	35.53	26.71	28.97	25.58	91.32	116.68	131.24
AA57G00255	65.42	57.59	78.97	98.84	139.61	48.16	46.54	54.14	44.63	46.25	37.37	196.06	294.35	387.47
AA60G00187	92.47	74.79	105.16	82.45	84.75	90.32	44.42	55.69	41.26	26.90	42.77	128.72	123.07	238.32
AA89G00051	90.07	71.86	111.88	112.12	108.33	92.55	74.09	81.99	73.83	51.59	59.41	175.74	249.12	283.84
AA102G00141	53.31	47.86	107.45	77.34	79.71	128.28	50.95	67.42	78.42	40.23	83.01	138.25	201.85	435.77

Supplemental Figure S2 continued next page…

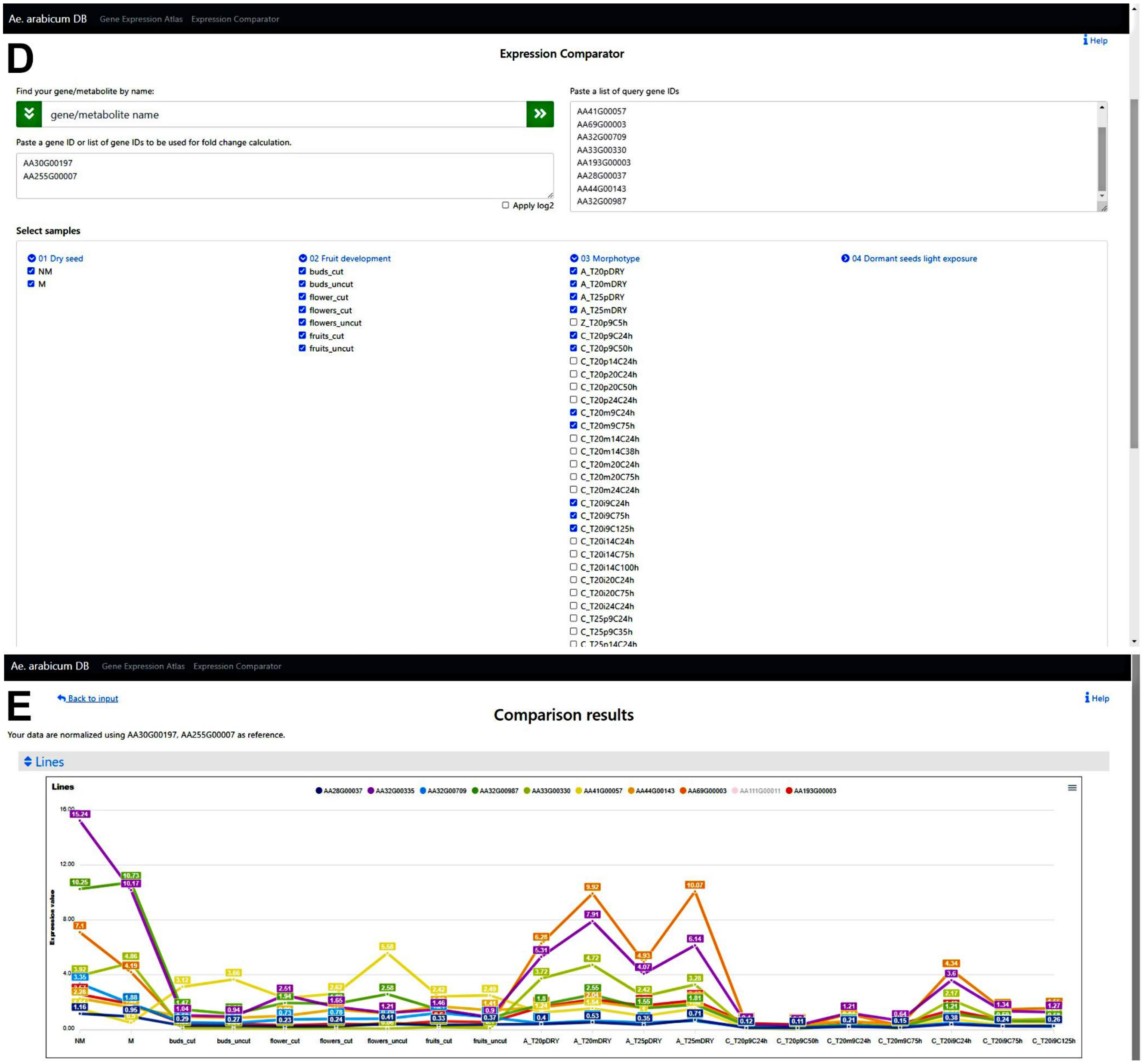

Supplemental Figure S2. *Aethionema arabicum* Gene Expression Atlas tool (https://plantcode.cup.uni-freiburg.de/easy_gdb/tools/expression/expression_input.php) (Supports Figures 2, 3, and 4). A, Genes with the highest module membership of the pink co-expressed gene module AA4G00252, AA31G00360, AA10G00208, AA60G00187, AA39G00802, AA57G00255, AA102G00141, AA32G00445, AA89G00051, AA8G00115, which was associated with pericarp removal (more highly expressed in imbibed bare M$^-$ seeds, pericarp removed) were used as a query in the Gene Expression Atlas tool using the '03 Morphotype' experiment dataset, the dataset used for this study. B, Expression trends in the atlas (line graph and heatmap) show the strong increase in the imbibed M$^-$ seed samples compared to M$^+$ and IND. Sample naming scheme used for the atlas is available in Supplementary Data Set S1. C, Separation of replicates across samples and average values as a table can also be visualized using the expression atlas. D, The associated expression comparator tool was used to visualize expression of the top ten yellow module genes AA111G00011, AA32G00335, AA41G00057, AA69G00003, AA32G00709, AA33G00330, AA193G00003, AA28G00037, AA44G00143, AA32G00987 in the

experiments of Wilhelmsson *et al.* (2019) on dry seed, Arshad *et al.* (2021) on fruit development and in dry and imbibed seeds from this study. The normalization option was used to normalize the expression to reference genes used for RT-qPCR in this study (AA30G00197, AA255G00007). E, The trends suggest that the yellow module genes are generally most highly expressed in dry seeds, including in Wilhelmsson *et al.* (2019). Expression may be relatively higher in buds, flowers and fruits than in imbibed M^+ and M^- seeds.

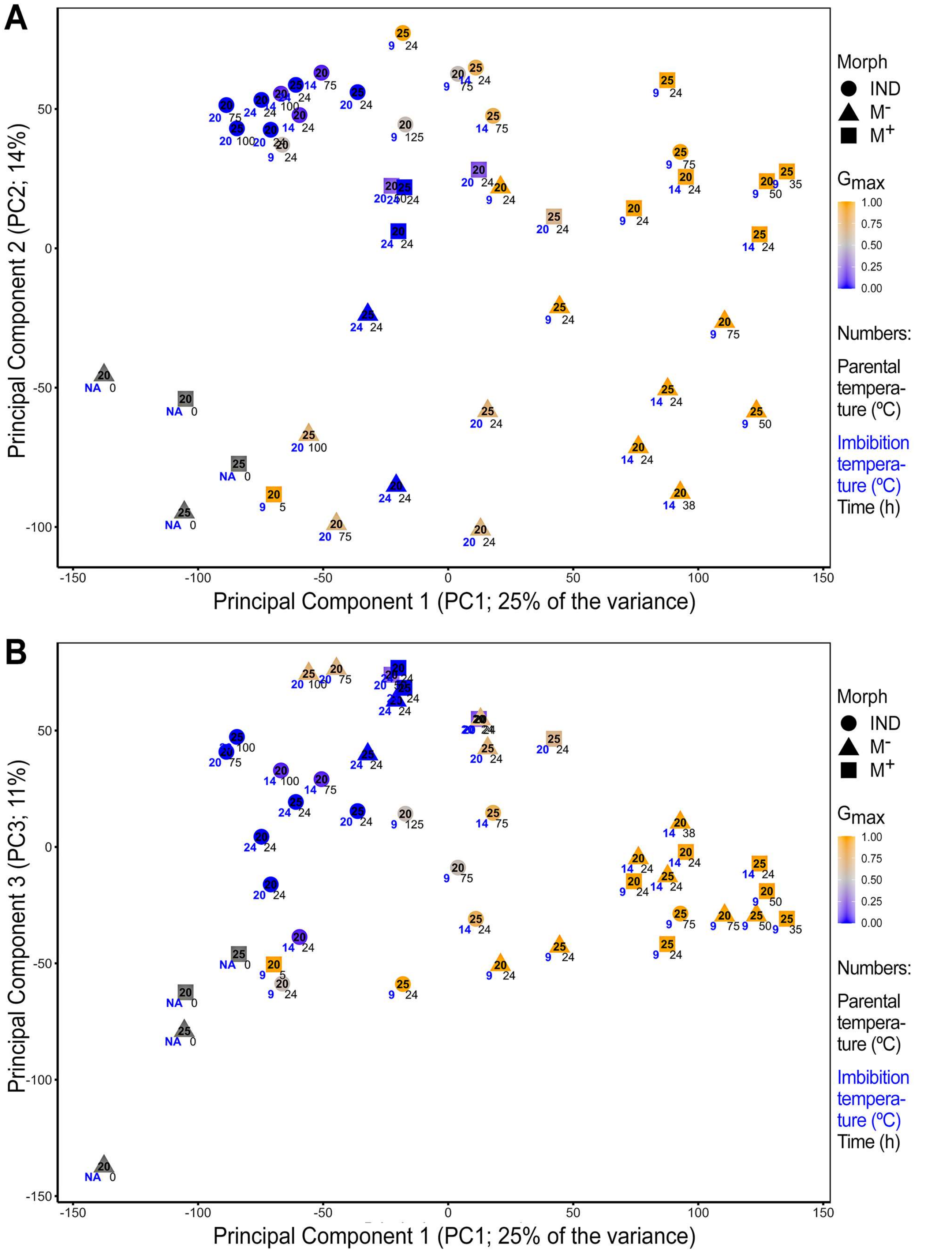
A
Principal Component 2 (PC2; 14%)
50
0
-50
-100
-150
-100
-50
0
50
100
150
Principal Component 1 (PC1; 25% of the variance)
Morph
IND
M-
M+
Gmax
1.00
0.75
0.50
0.25
0.00
Numbers:
Parental tempera-ture (ºC)
Imbibition tempera-ture (ºC)
Time (h)
B
Principal Component 3 (PC3; 11%)
50
0
-50
-100
-150
Principal Component 1 (PC1; 25% of the variance)
Morph
IND
M-
M+
Gmax
Numbers:
Parental tempera-ture (ºC)
Imbibition tempera-ture (ºC)
Time (h)

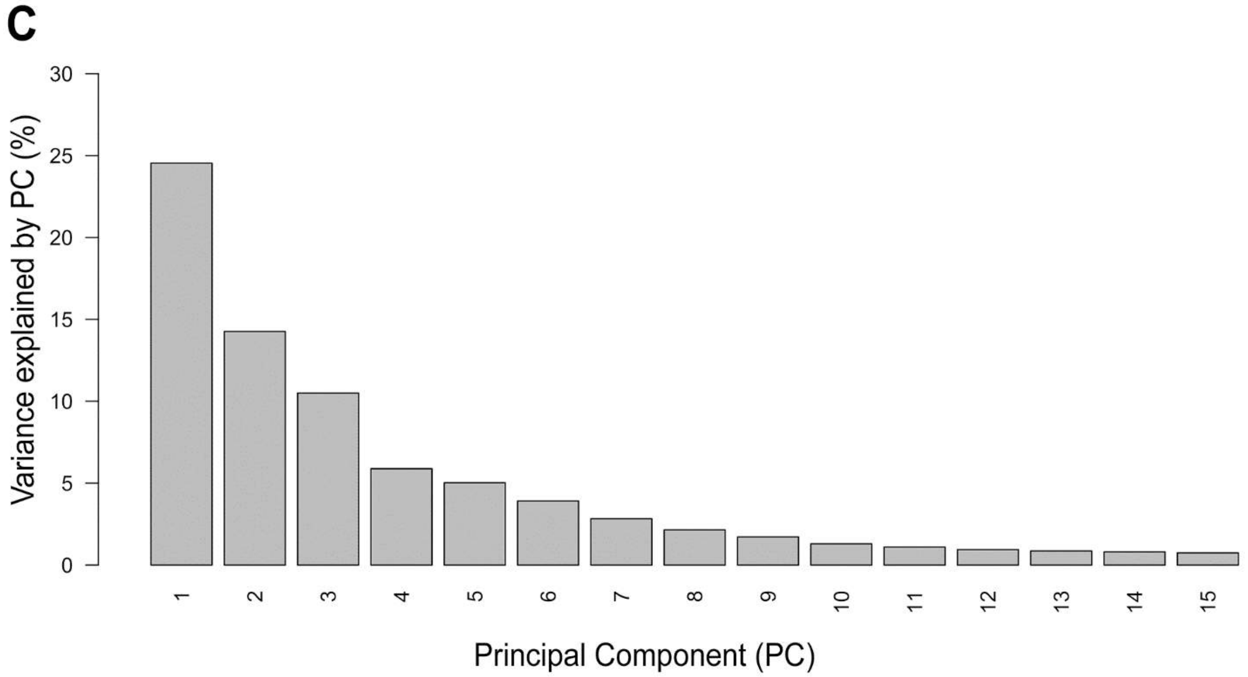

Supplemental Figure S3. Principal components analysis (PCA) comparing the seed mRNA transcriptome data (RNA-sequencing analysis) of *Aethionema arabicum* (Supports Figure 2). A, Average PCA coordinates for PC2 versus PC1 (Figure 2) and samples shaded relative to final germination percentages (G_{max}) comparing diaspore morphs, maternal and imbibition temperatures. Icon shape represents morph, lower left bold blue text indicates imbibition temperature, lower right black text indicates time-point, central bold black text indicates maternal temperature, and shading indicated G_{max}. B, Average PCA coordinates for PC3 versus PC1. C, Variance explained by the top 15 PCs. Note that a broad trend was observed following imbibition in that under conditions that are generally germination-permissive, with increasing imbibition time, samples travel positively along PC1 (25% of variance). PC2 (14% of variance) on the other hand appears to generally separate IND and M^+ from bare M^- seed. PC3 (11% of variance) may have some relation to imbibition temperature. PCA plots were drawn in R with the ggplot2 package (v3.3.6; Wickham, 2016).

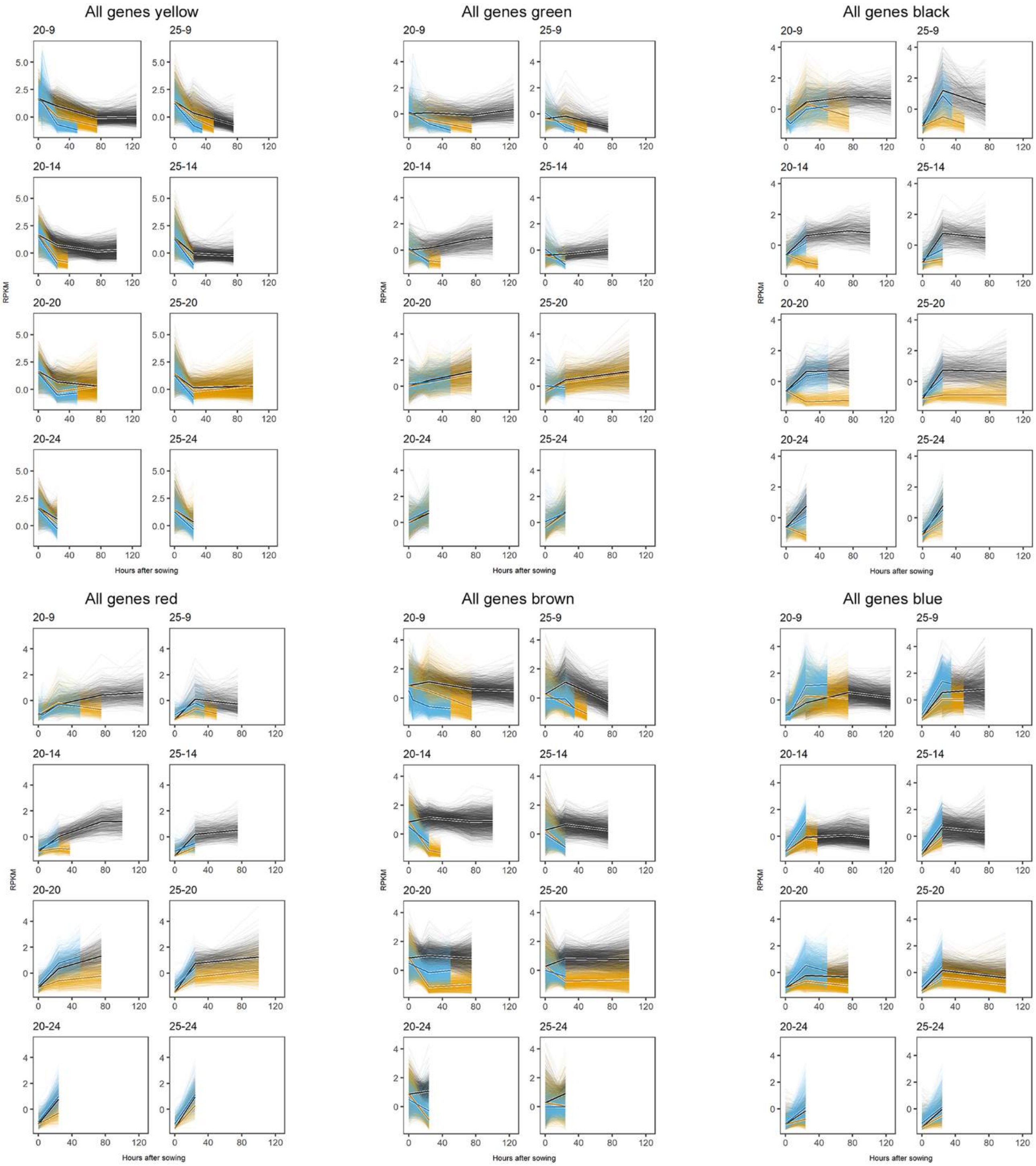

Supplemental Figure S4 continued next page…

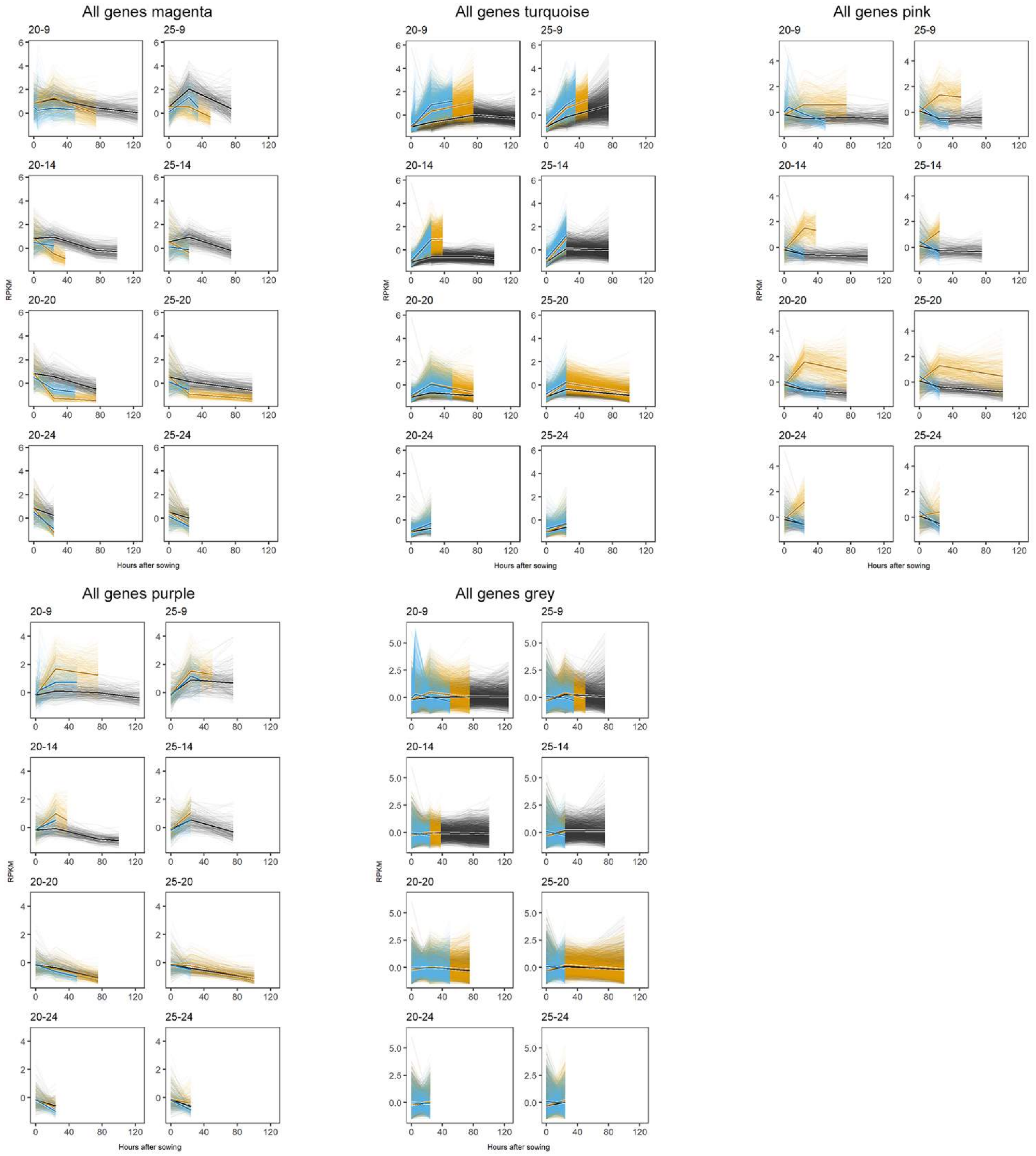

Supplemental Figure S4. Expression of WGCNA modules over all *Aethionema arabicum* samples (Supports Figure 3), M^+ and M^- seeds and IND fruits from two maternal temperatures (20ºC and 25ºC), at four imbibition temperatures (9ºC, 14ºC, 20ºC and 24ºC), and multiple time-points. Panels show sample means (thick lines) of mean Z-score expression of module member genes as well as sample mean Z-scores of each individual module gene member (thin lines, 'spaghetti plots). Modules are referred to by color (yellow, red, green, brown, black, blue, magenta, purple, turquoise, grey, pink) as detailed in Figure 3. Plots drawn in R with ggplot2 (v3.1.0; Wickham, 2016).

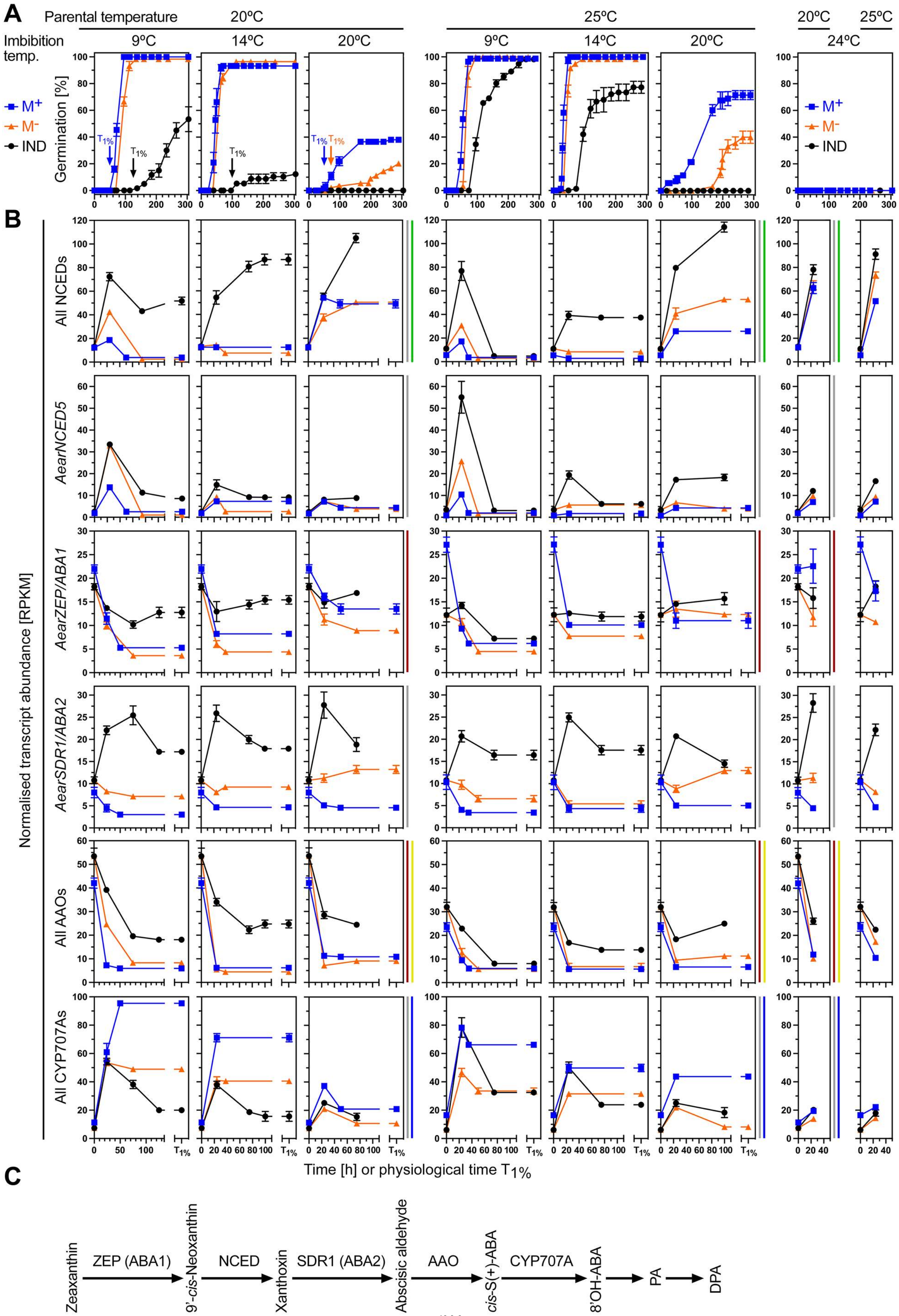
A
Parental temperature
20ºC
25ºC
20ºC
25ºC
Imbibition temp.
9ºC
14ºC
20ºC
24ºC
Germination [%]
M+
M-
IND
T1%
B
Normalised transcript abundance [RPKM]
All NCEDs
AearNCED5
AearZEP/ABA1
AearSDR1/ABA2
All AAOs
All CYP707As
Time [h] or physiological time T1%
C
Zeaxanthin
ZEP (ABA1)
9'-cis-Neoxanthin
NCED
Xanthoxin
SDR1 (ABA2)
Abscisic aldehyde
AAO
cis-S(+)-ABA
CYP707A
8'OH-ABA
PA
DPA

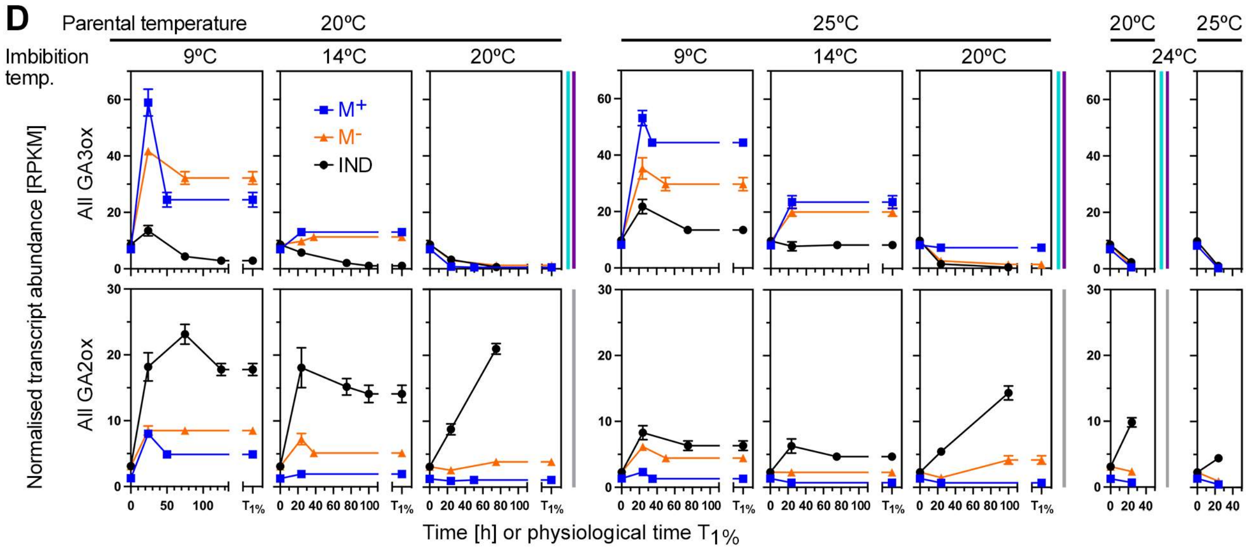

Supplemental Figure S5. Temperature responses and ABA and GA metabolism (Supports Figures 4 and 5). Comparative analysis of germination responses at different temperatures and associated abscisic acid (ABA) and gibberellin (GA) metabolism gene transcript abundance patterns of *Aethionema arabicum* dimorphic diaspores. A, Dimorphic diaspores (M^+ seeds, IND fruits) and bare M^- seeds (extracted from IND fruits) from two maternal temperature regimes during reproduction (20ºC versus 25ºC) were compared for their kinetics of germination at four different imbibition temperatures (9, 14, 20 and 24ºC). Comparative results were obtained for physical (in hours) and physiological time-points ($T_{1\%}$, representing the population's onset of germination completion). Mean ± SEM values of 3 replicates each with 20 seeds. B, Normalized transcript abundances in reads per kilobase per million (RPKM) from the transcriptomes (RNA-seq) are presented for the ABA metabolism genes 9-*cis*-epoxycarotenoid dioxygenase (*AearNCED*), zeaxanthin epoxidase (*AearZEP/ABA1*), short-chain dehydrogenase/reductase (*AearSDR1/ABA2*), abscisic aldehyde oxidase (*AearAAO*), and ABA 8′-hydroxylase (*AearCYP707A*). WGCNA modules (Figure 3) for these genes are indicated by the vertical color lines next to the graphs. Mean ± SEM values of 3 replicates each with 60-80 (RNA-seq) seeds. C, Simplified ABA biosynthesis and degradation pathway. Major metabolites and associated enzymes are indicated; phaseic acid (PA) and dihydrophaseic acid (DPA) are generated non-enzymatically. D, Normalized transcript abundances from the transcriptomes (RNA-seq) for gibberellin GA3-oxidases and GA2-oxidases which catalyze the formation of bioactive GAs from inactive precursors and the inactivation of bioactive GAs, respectively. For *Ae. arabicum* gene names and IDs see Supplemental Table S2 or the Gene Expression Atlas (https://plantcode.cup.uni-freiburg.de/easy_gdb/tools/expression/expression_input.php); for RNAseq single values see the Expression Atlas or Supplemental Data Set S1. For gene IDs from and modules of the presented expression results (individual or as cumulative sum) see Supplemental Table S2.

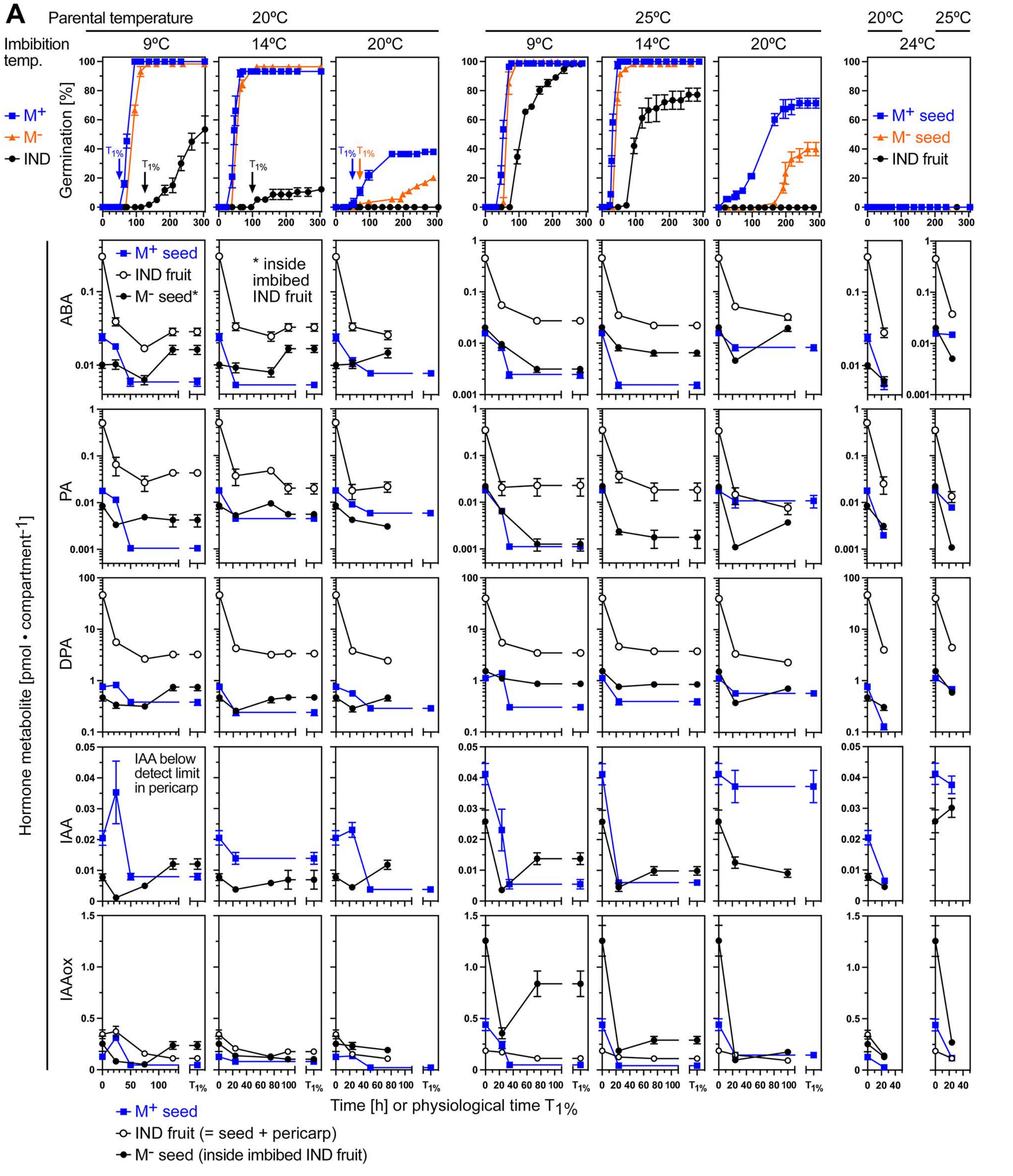

Supplemental Figure S6 continued next page…

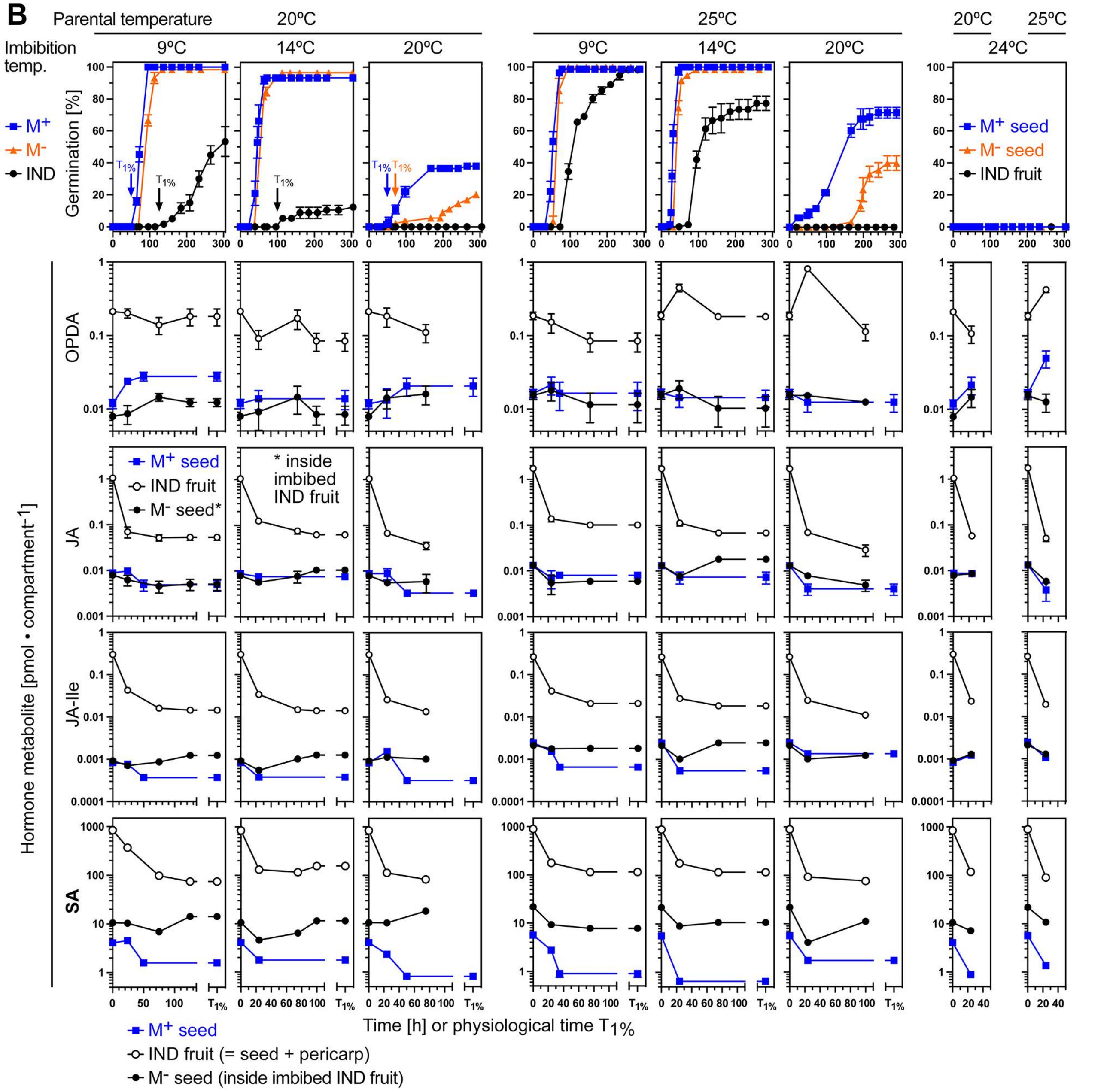

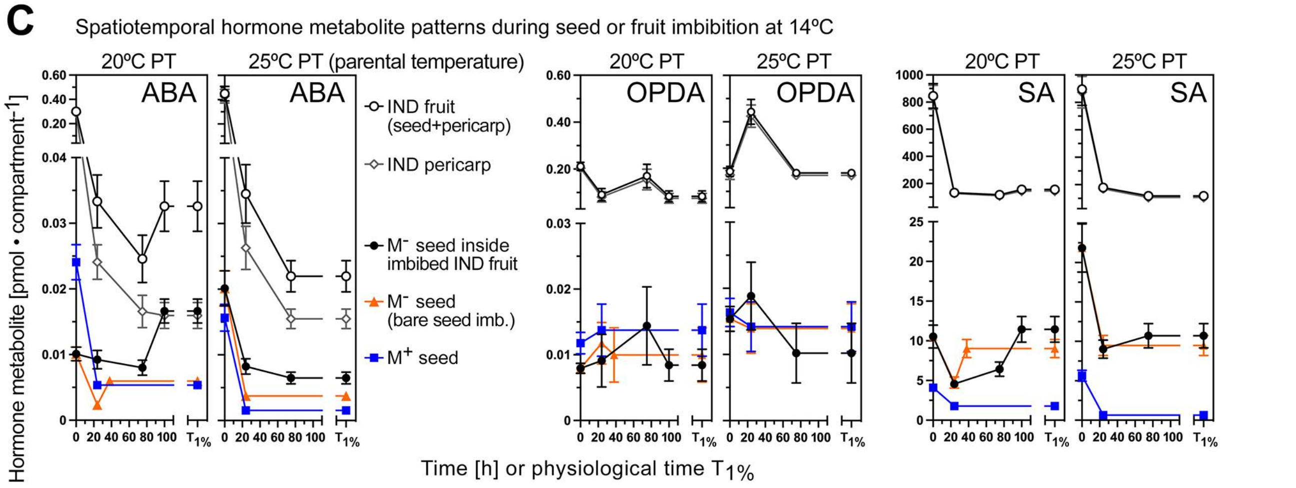

Supplemental Figure S6 continued next page…

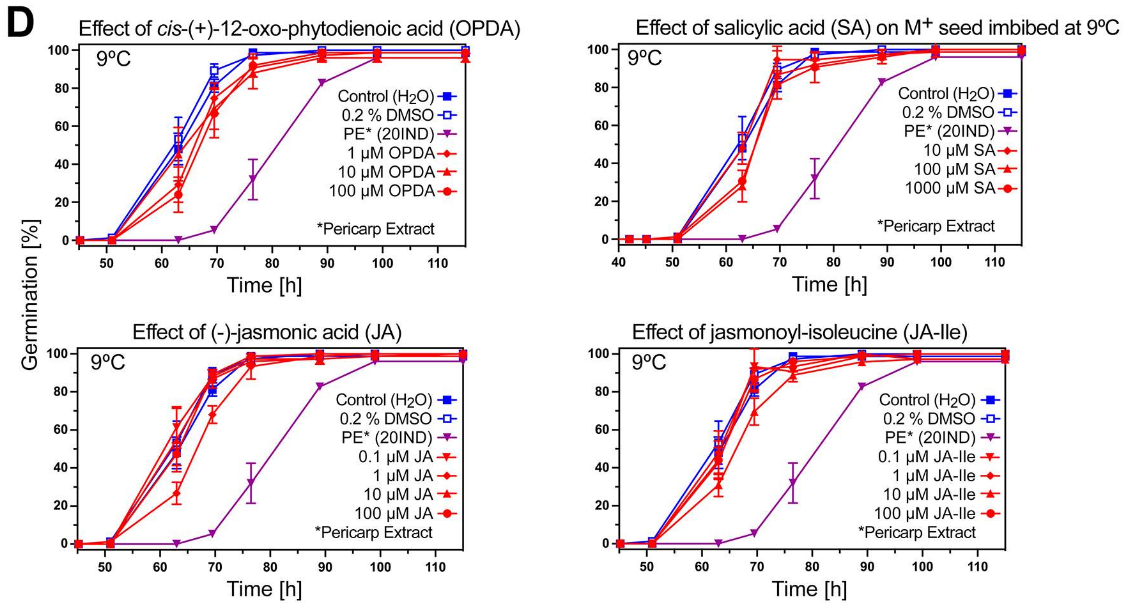

Supplemental Figure S6. Comparative analysis of *Aethionema arabicum* dimorphic diaspore germination responses at different temperatures, associated hormone metabolite contents and effects of hormones (Supports Figure 6). A, Dimorphic diaspores (M^+ seeds, IND fruits) and bare M^- seeds (extracted from IND fruits) from two maternal temperature regimes during reproduction (20ºC versus 25ºC) were compared for their kinetics of germination and hormone metabolite contents at four different imbibition temperatures (9, 14, 20 and 24ºC). Comparative results were obtained for physical (in hours) and physiological time-points ($T_{1\%}$, representing the population's onset of germination completion). Hormone metabolites contents in pericarp and in M^+ and M^- seeds presented: abscisic acid (ABA) and ABA degradation products phaseic acid (PA) and dihydrophaseic acid (DPA), and salicylic acid (SA). B, Comparative analysis of indole-3-acetic acid (IAA), 2-oxoindole-3-acetic acid (IAAox), *cis*-(+)-12-oxophytodienoic acid (OPDA), jasmonic acid (JA) and its isoleucine conjugate (JA-Ile) contents in pericarp and in M^+ and M^- seeds. C, Spatiotemporal ABA, OPDA and SA patterns during seed or fruit imbibition at 14ºC. D, Effect of OPDA, JA, JA-Ile, and SA on M^+ seed germination. Mean ± SEM values of 3 (germination) or 5 (metabolites) biological replicate samples each with 20 (germination) or 30-40 (metabolites) are presented.

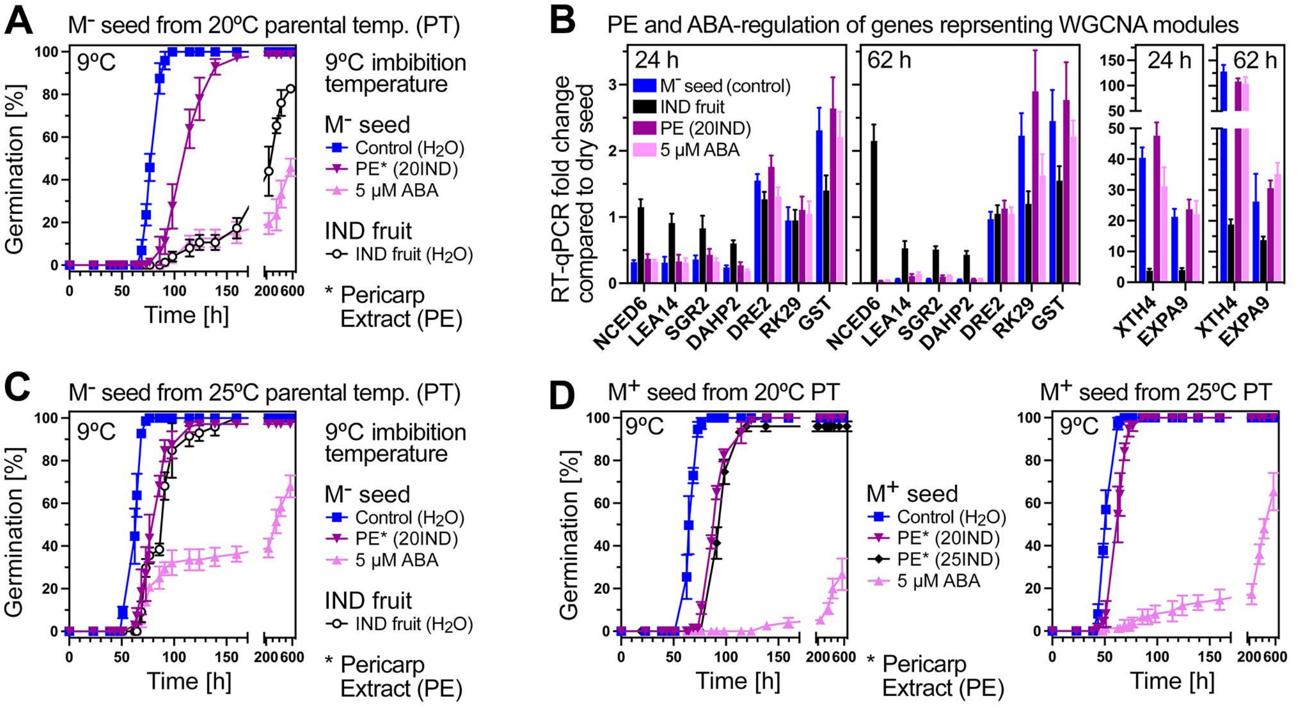

Supplemental Figure S7. Effects of IND pericarp extract (PE) and abscisic acid (ABA) of *Aethionema arabicum* dimorphic diaspore germination and expression of selected genes representing the WGCNA modules (Supports Figures 6 and 8). A, The effect pf IND PE and ABA on the germination of bare M⁻ seeds (obtained from IND fruits by pericarp removal) from plants grown at 20ºC parental termperature (PT). B, The effect of PE and ABA on the expression of selected genes representing the WGCNA modules. Note that the corresponding effect of hypoxia on the expression of these genes is presented in Figure 8B. For *Ae. arabicum* gene names and IDs see Supplemental Table S2 or the Expression Atlas (https://plantcode.cup.uni-freiburg.de/aetar_db/index.php). C, The effect pf IND PE and ABA on the germination of bare M⁻ seeds from plants grown at 20ºC PT. D, The effect pf IND PE and ABA on the germination of M⁺ seeds from plants grown at 20ºC or 25ºC PT. Mean ± SEM values of 3 (germination, RT-qPCR) biological replicate samples are presented.

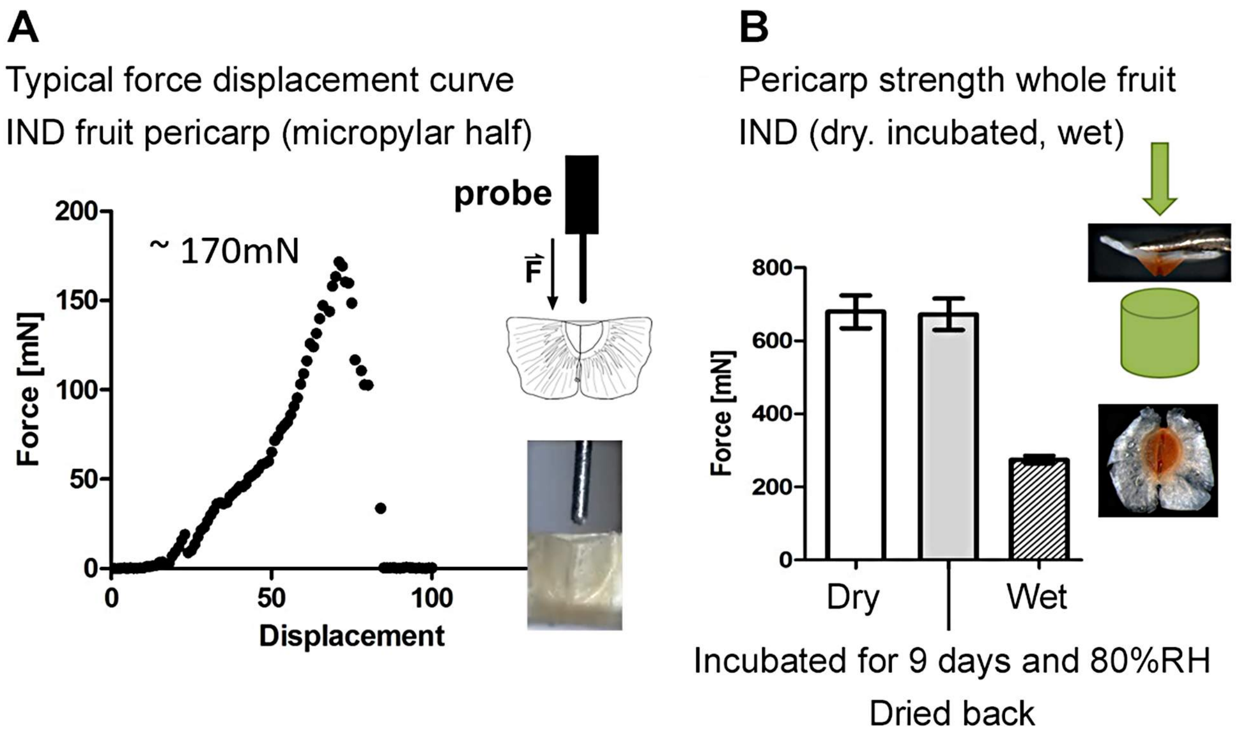

Supplemental Figure S8. *Aethionema arabicum* pericarp (fruit coat) biomechanics (Supports Figure 6). A, Typical force-displacement curve of a IND fruit pericarp. Fruits have been cut in half, seeds removed and the dry, empty fruits halves have been probed with a rounded 0.3 mm needle. B, pericarp strength of whole IND fruits. Fruit was laid flat on a sample holder and a 0.3 mm steel probe was driven into the sample while force and displacement were measured simultaneously. Dry fruits show no significant difference to re-dried fruits that had been incubated for 9 days at 80% relative humidity. Imbibed fruits (3 h in water) show a significantly lower pericarp resistance.

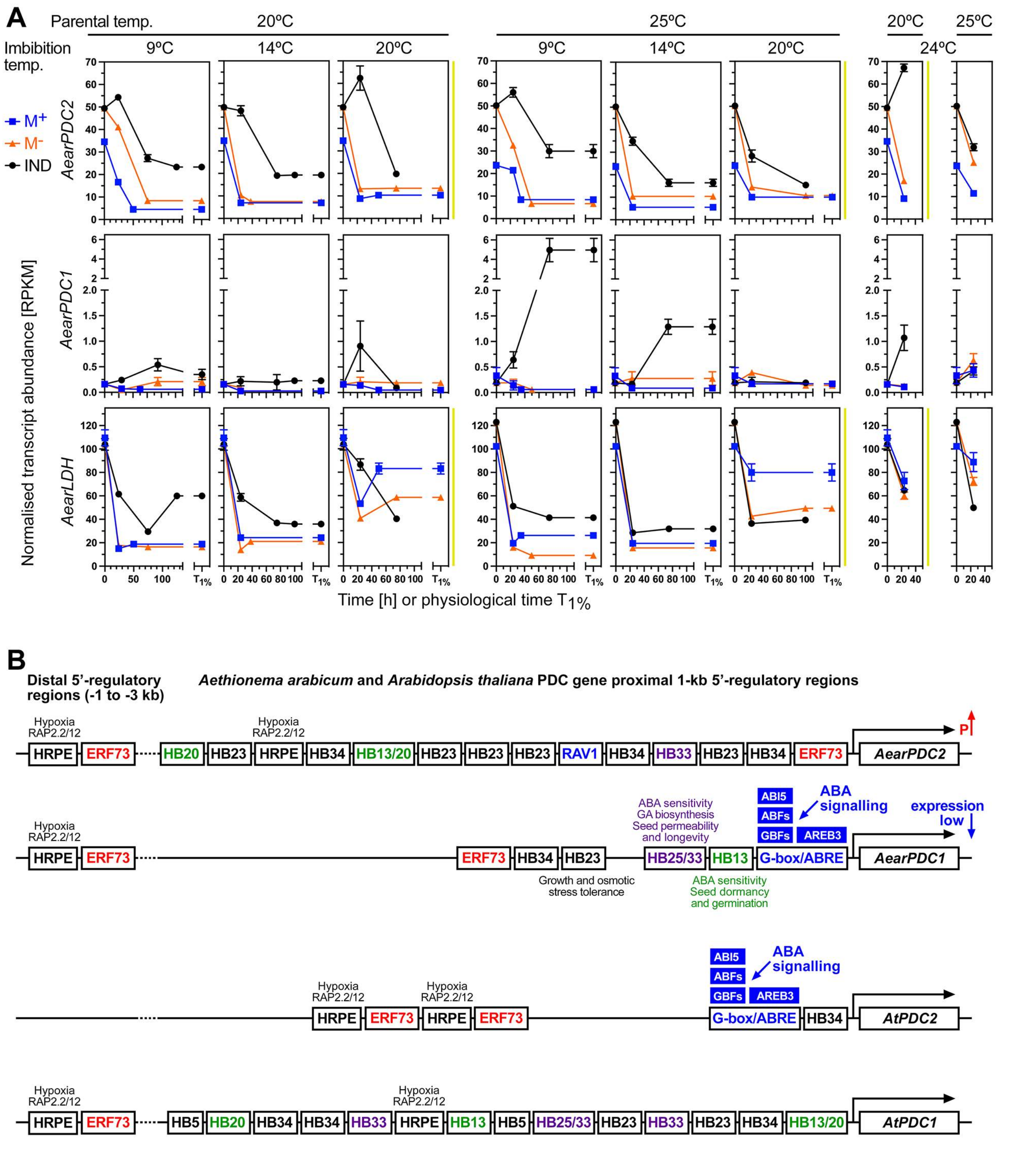

Supplemental Figure S9. Transcription factor (TF) and target *cis*-regulatory motif analysis of *Aethionema arabicum* gene expression with focus on hypoxia and ABA related genes (Supports Figure 7). A, Transcript abundance patterns (RNA-seq) of the *Ae. arabicum* pyruvate decarboxylase (*AearPDC2* and *AearPDC1*) and lactate dehydrogenase (*AearLDH*) genes in seeds of imbibed dimorphic diaspores (M^+ seeds, IND fruits) and bare M^- seeds (extracted from IND fruits) from two maternal temperature regimes (20ºC versus 25ºC) at four different imbibition temperatures (9, 14, 20 and 24ºC). WGCNA modules (Figure 3) for these genes are indicated by the vertical color lines next to the graphs. Mean ± SEM values of 3 replicates each with 60-80 seeds are presented. C, Addition to working model for the pericarp-mediated hypoxia up-regulation (P↑) and ABA signaling (Figure 6C) for

the *A. thaliana* and *Ae. arabicum* PDC genes. Motifs indicated include the hypoxia-responsive promoter element HRPE, the G-box and ABA-responsive element (ABRE), the ERF73 *cis*-regulatory element and HB-motifs for the binding of homeobox TFs. These motifs are the targets for the AearERF71/73 TF and the ABA related ABI5, ABF (ABRE-binding factors), GBF (G-box-binding factors), and AREB3 TFs (*blue boxes*). For details about the *cis*-regulatory motif analysis see Supplemental Figure S10. The *AearADH1a* 5'-regulatory gene region contains ERF73 and HRPE motifs and is distinct from the *AtADH1* and *AearADH1b* 5'-regulatory gene regions in that it does not contain G-box/ABRE and HB binding motifs (Figure 7C). The *AearERF71/73* 5'-regulatory gene region was also distinct from its *A. thaliana* homologs by the presence of two ERF73 and one HB13/20 motif, suggesting that the *AearERF71/73* gene possibly provides a positive feedback regulation on the pericarp/hypoxia-mediated *AearADH1a* and *AearERF71/73* expression (Figure 7C). Further support for the importance of these motifs is that the pericarp/hypoxia-induced *AearPDC2* 5'-regulatory gene region also contains these motifs, as well as no G-box/ABRE motifs. In contrast to this, the low expressed *AearPDC1* and *AearADH1b* 5'-regulatory regions contain, as the *AtPDC2* and *AtADH1* gene promoters, in addition G-box/ABRE motifs (Figure 7C; Supplemental Figure S10). Supplemental Figure S10 provides a more detailed comparative analysis of *Ae. arabicum* and *A. thaliana* 5'-regulatory gene regions, including for *AearDOG1* (Figure 7C). For *Ae. arabicum* gene names and IDs see Supplemental Table S2 or the Gene Expression Atlas (https://plantcode.cup.uni-freiburg.de/easy_gdb/tools/expression/expression_input.php); for RNAseq single values see the Expression Atlas or Supplemental Data Set S1. For gene IDs from and modules of the presented expression results (individual or as cumulative sum) see Supplemental Table S2.

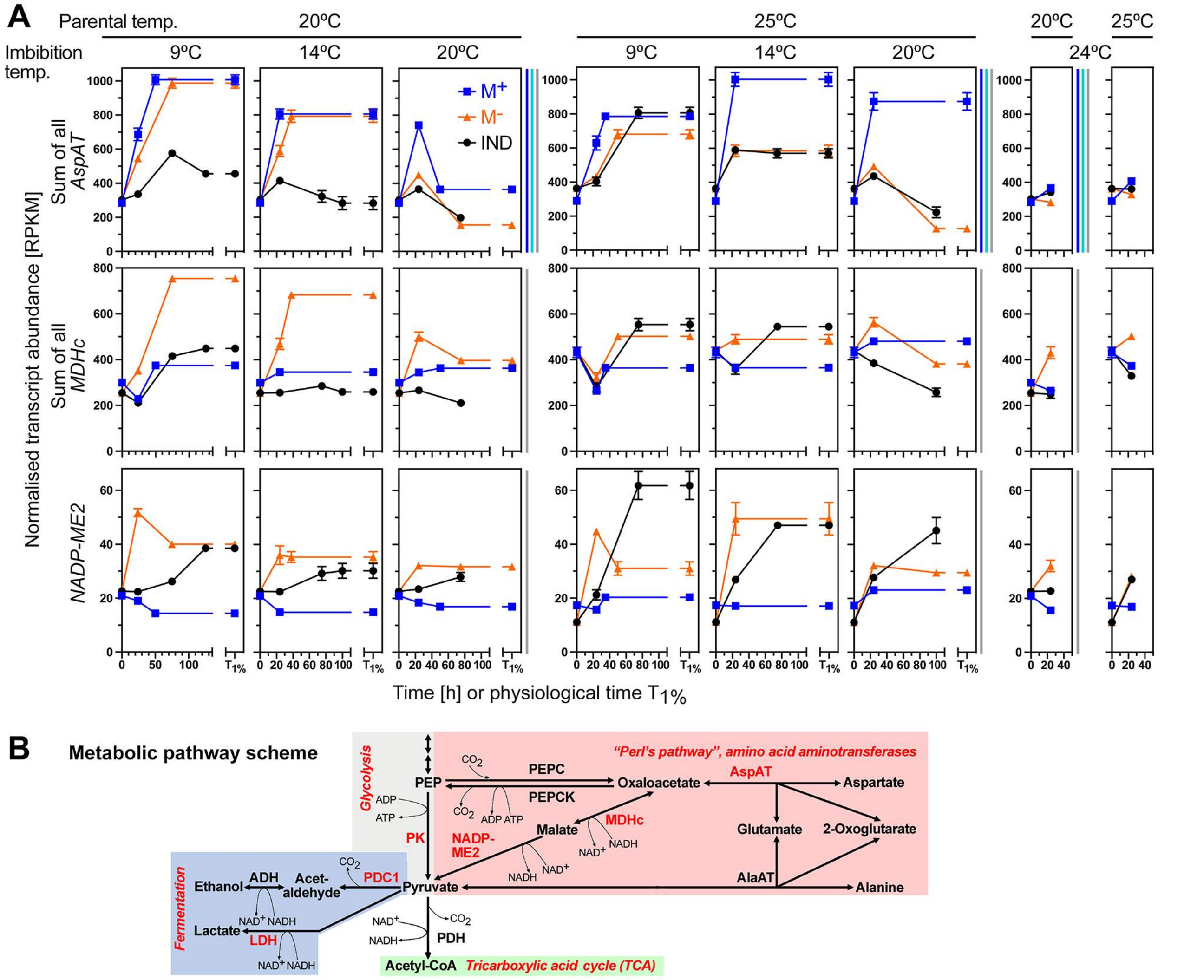

Supplemental Figure S10. Expression patterns of *Aethionema arabicum* genes involved in the seed-specific "Perl's pathway" (Supports Figure 7). A, Transcript abundance patterns (RNA-seq) of *Ae. arabicum* aspartate amino tranferase (*AspAT*), cytosolic malate dehydrogenase (*MDHc*), and NADP-dependent malic enzyme (*NADP-ME2*) genes in seeds of imbibed dimorphic diaspores (M^+ seeds, IND fruits) and bare M^- seeds (extracted from IND fruits) from two maternal temperature regimes (20ºC versus 25ºC) at four different imbibition temperatures (9, 14, 20 and 24ºC). WGCNA modules (Figure 3) for these genes are indicated by the vertical color lines next to the graphs. Mean ± SEM values of 3 replicates each with 60-80 seeds are presented. B, Scheme of the "Perl's pathway" in *A. thaliana*, for details about this metabolic shunt pathway see Weitbrecht *et al.* (2011). For gene IDs from and modules of the presented expression results (individual or as cumulative sum) see Supplemental Table 2. For *Ae. arabicum* gene names and IDs see Supplemental Table S2 or the Gene Expression Atlas (https://plantcode.cup.uni-freiburg.de/easy_gdb/tools/expression/expression_input.php); for RNAseq single values see the Expression Atlas or Supplemental Data Set S1.

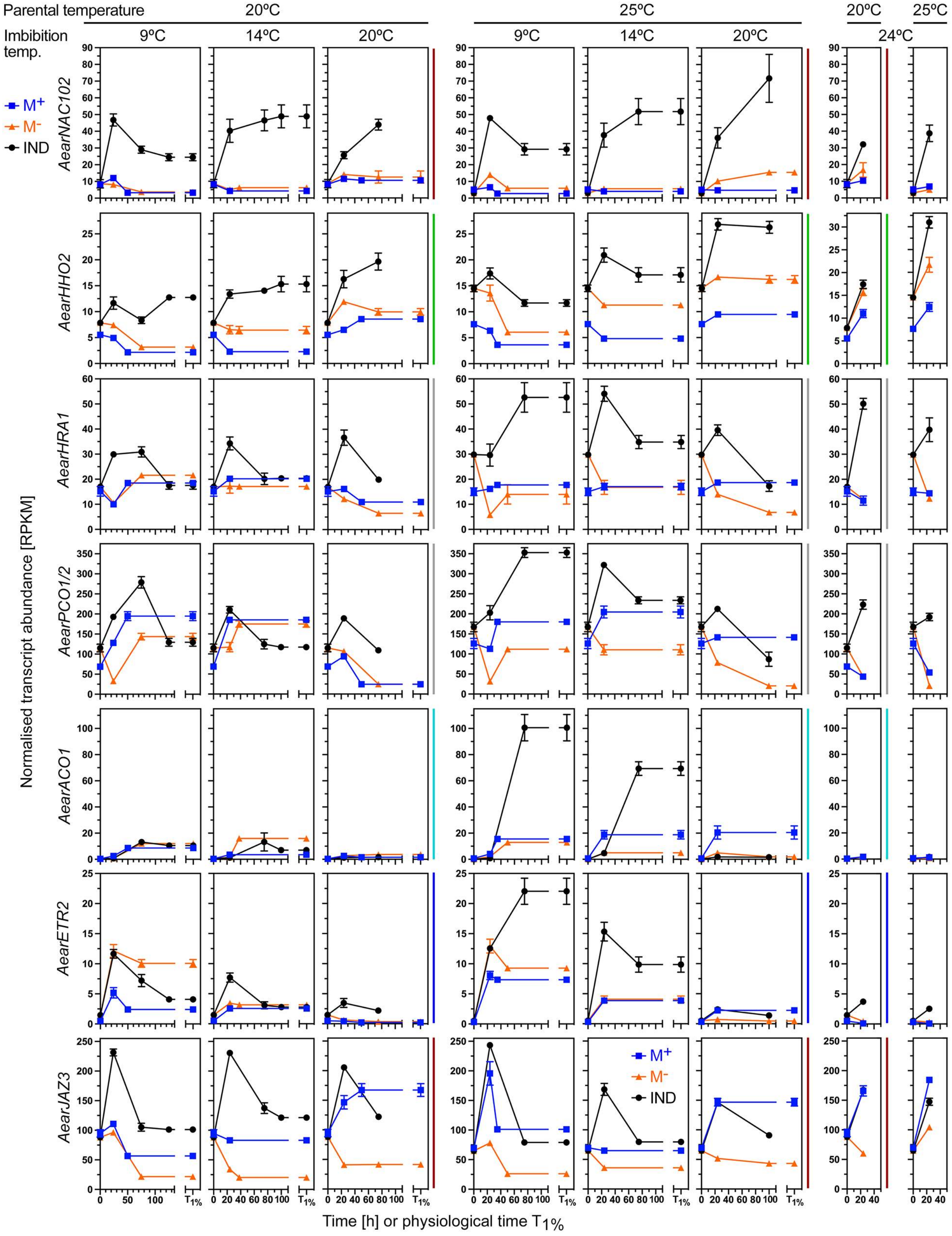

Supplemental Figure S11. Expression patterns of *Aethionema arabicum* genes known to be regulated by hypoxia in *Arabidopsis thaliana* seedlings (Supports Figure 7). Transcript abundance patterns (RNA-seq) of *Ae. arabicum* genes in seeds of imbibed dimorphic diaspores (M^+ seeds, IND fruits) and M^-

seeds (extracted from IND fruits) from two maternal temperature regimes (20ºC versus 25ºC) at four different imbibition temperatures (9, 14, 20 and 24ºC). WGCNA modules (Figure 3) for these genes are indicated by the vertical color lines next to the graphs. Mean ± SEM values of 3 replicates each with 60-80 seeds are presented. Examples presented (references for *A. thaliana* hypoxia experiments): NAC-domain containing TF *AearNAC102* (*NAM* (*no apical meristem*), *ATAF1,2 and CUC2* (*cup-shaped cotyledon*)) (Christianson et al., 2009), Myb-type TF *AearHHO2* (*HRS1* (*hypersensitive to low Pi-elicited primary root shortening*) homolog), *AearHAR1* (receptor kinase), plant cysteine oxidase *AearPCO1/2* (Lee et al., 2019), 1-aminocyclopropane-1-carboxylic acid oxidase *AearACO1*, *AearETR1* (*Ethylene response 1*), *AearJAZ3* (*Jasmonate-zim-domain protein*) (Ju et al., 2019; Gasch et al., 2016). For *Ae. arabicum* gene names and IDs see Supplemental Table S2 or the Gene Expression Atlas (https://plantcode.cup.uni-freiburg.de/easy_gdb/tools/expression/expression_input.php); for RNAseq single values see the Expression Atlas or Supplemental Data Set S1. For gene IDs from and modules of the presented expression results (individual or as cumulative sum) see Supplemental Table S2.

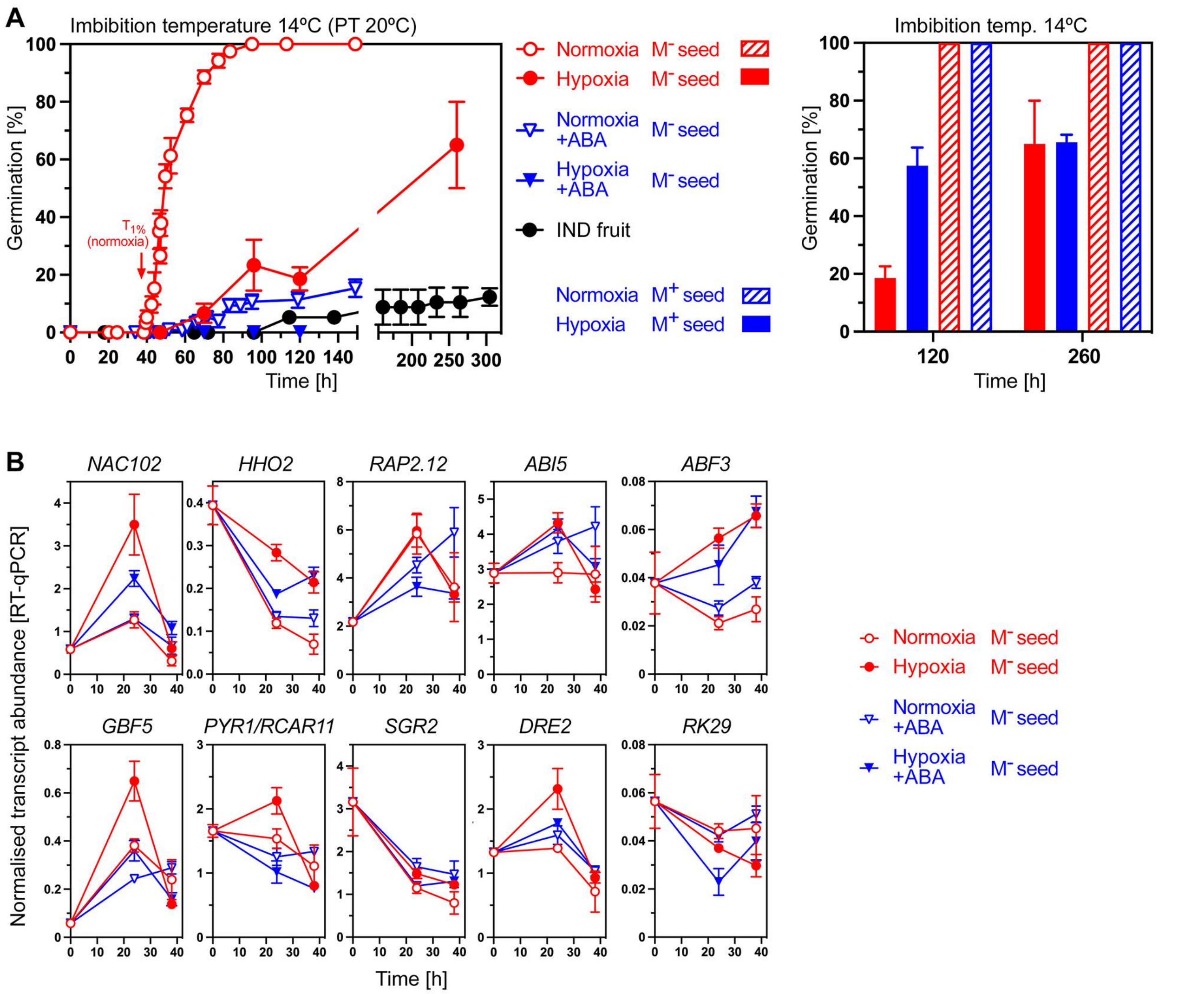

Supplemental Figure S12 continued next page…

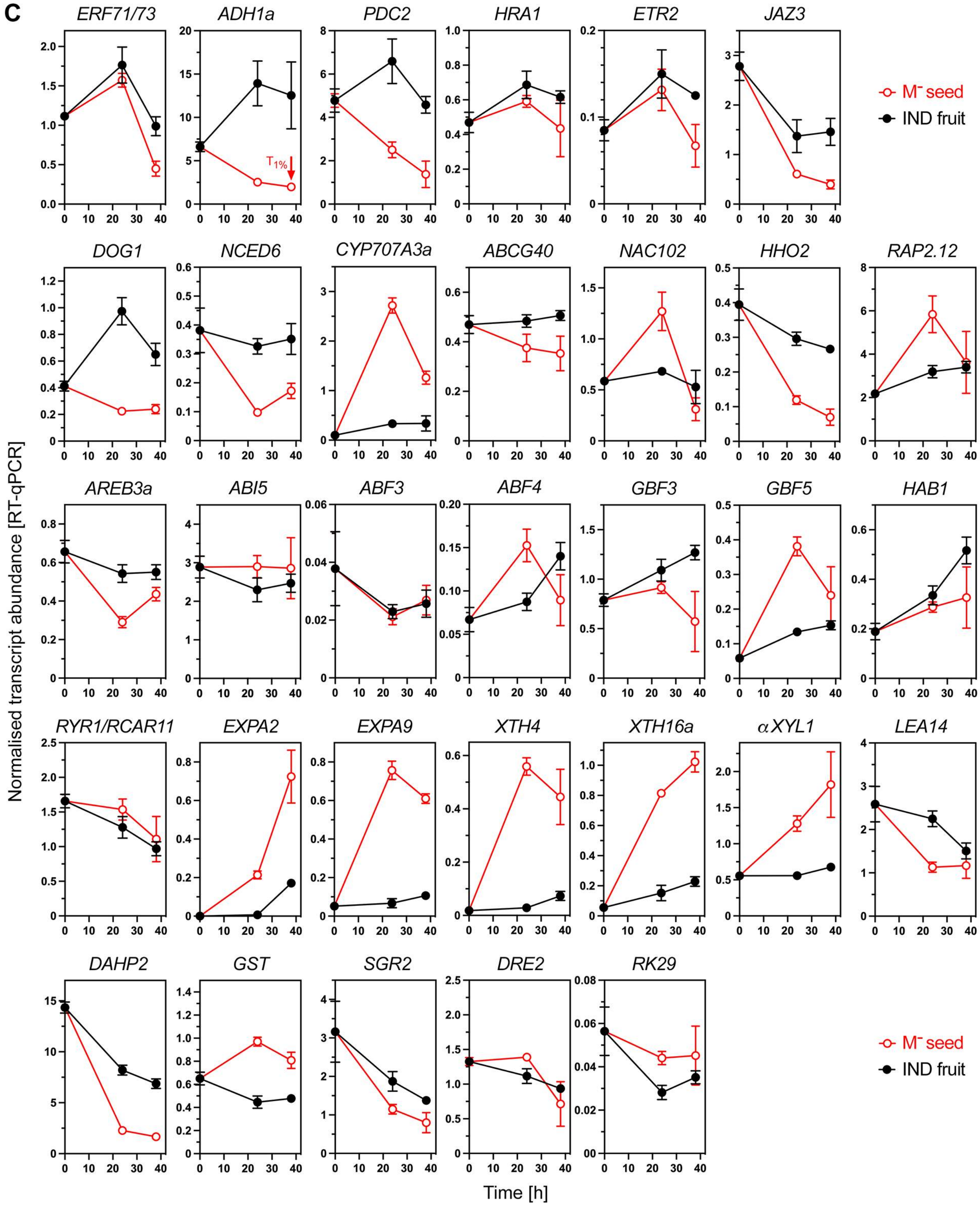

Supplemental Figure S12. Comparative analysis of germination and gene expression in IND fruits and bare M⁻ seeds as affected by hypoxia and abscisic acid (ABA) and the pericarp (Supports Figure 8). A, The effect of hypoxia (4.5±0.2% oxygen) on the germination of bare M⁻ seeds and M⁺ seeds at 14ºC in continuous white light. B, RT-pPCR expression analysis of selected genes during *Aethionema arabicum* bare M⁻ seed imbibition (14ºC, continuous light) under hypoxia (4.5±0.2% oxygen) and normoxia (21% oxygen) conditions ± 5 µM abscisic acid (ABA). The 38 h timepoint (arrow) corresponds to $T_{1\%}$ of the control (normoxia without ABA). For additional genes and expression in IND fruits see Figure 8. C, RT-

pPCR expression analysis of selected genes during *Aethionema arabicum* IND fruit and bare M^- seed imbibition under control (normoxia) conditions. Bare M^- seeds were obtained from dry IND fruits by pericarp removal and imbibed at 14ºC in continuous light. The 38 h timepoint (arrow) corresponds to $T_{1\%}$ of the control (normoxia). Mean ± SEM values of 3 (germination, RT-qPCR) biological replicate samples are presented. Differential gene expression was assessed using statistical tests (Supplemental Table S4). For *Ae. arabicum* gene names and IDs see Supplemental Table S2 or the Expression Atlas (https://plantcode.cup.uni-freiburg.de/aetar_db/index.php).

A 5'-regulatory motif analyses - FIMO and AME motif search tool best hits:

MOTIF	WIDTH	BEST POSSIBLE MATCH	TF
AP2EREBP_tnt.ERF73_col_a_m1	21	CCACCGCCGCCGCCATTTCCG	ERF73
AP2EREBP_tnt.ERF73_colamp_a_m1	21	ATGGCGGCGGCGGCGGCGGCG	ERF73
HRPEGasch2016	12	GCCCCTGGTTTT	HRPE
AP2EREBP_tnt.RAP212_col_a_m1	21	CGGCGGAAATGGCGGCGGAGG	RAP2.12
bZIP_tnt.ABI5_col_v3h_m1	18	AAATGGTGACGTGGCAGT	ABI5
bZIP_tnt.ABI5_colamp_v3b_m1	15	AATGGTGACGTGGCA	ABI5
bZIP_tnt.AREB3_col_v31_m1	15	AATGGACACGTGGCA	AREB3
bZIP_tnt.AREB3_colamp_a_m1	15	AATGGACACGTGGCA	AREB3
bZIP_tnt.ABF2_col_v3a_m1	18	AAAAATGCCACGTGACCA	ABF2
bZIP_tnt.GBF3_col_m1	15	TGCCACGTCAGCATT	GBF3
bZIP_tnt.GBF3_colamp_m1	15	AATGGTCACGTGGCA	GBF3
bZIP_tnt.GBF5_col_v3a_m1	15	AATGCTGACGTGGCA	GBF5
bZIP_tnt.GBF5_colamp_a_m1	15	AATGCTGACGTGGCA	GBF5
bZIP_tnt.GBF6_col_m1	15	TGCCACGTCAGCATC	GBF6
bZIP_tnt.GBF6_colamp_a_m1	15	AATGCTGACGTGGCA	GBF6
RAV_tnt.RAV1_col_m1	17	CAGATAATTTCTGTTGT	RAV1
RAV_tnt.RAV1_colamp_a_m1	21	TTTTCAGGTGATTTCTGTTGT	RAV1
Homeobox_tnt.ATHB13_col_a_m1	11	TCAATAATTAA	HB13
Homeobox_tnt.ATHB13_colamp_a_m1	11	TCAATAATTGA	HB13
Homeobox_tnt.ATHB20_col_a_m1	11	TCAATAATTGA	HB20
Homeobox_tnt.ATHB20_colamp_a_m1	11	TCAATTATTGA	HB20
ZFHD_tnt.ATHB23_col_b_m1	22	AATCTTAATTAATTAATAAATT	HB23
ZFHD_tnt.ATHB23_colamp_a_m1	11	CTTAATTAATT	HB23
ZFHD_tnt.ATHB33_col_a_m1	11	ACGTAATTAAT	HB33
ZFHD_tnt.ATHB33_colamp_a_m1	15	AAAAACGTAATTAAT	HB33
ZFHD_tnt.ATHB25_col_a_m1	15	TAATTAATTAAGTTT	HB25
ZFHD_tnt.ATHB25_colamp_a_m1	11	CGTAATTAATG	HB25
ZFHD_tnt.ATHB34_col_a_m1	15	TTTAATCATTAATTA	HB34
ZFHD_tnt.ATHB34_colamp_a_m1	15	TAAAACATGATTAAT	HB34
HB_tnt.ATHB5_col_a_m1	11	TCAATAATTGA	HB5
HB_tnt.ATHB5_colamp_a_m1	10	CAATGATTGA	HB5

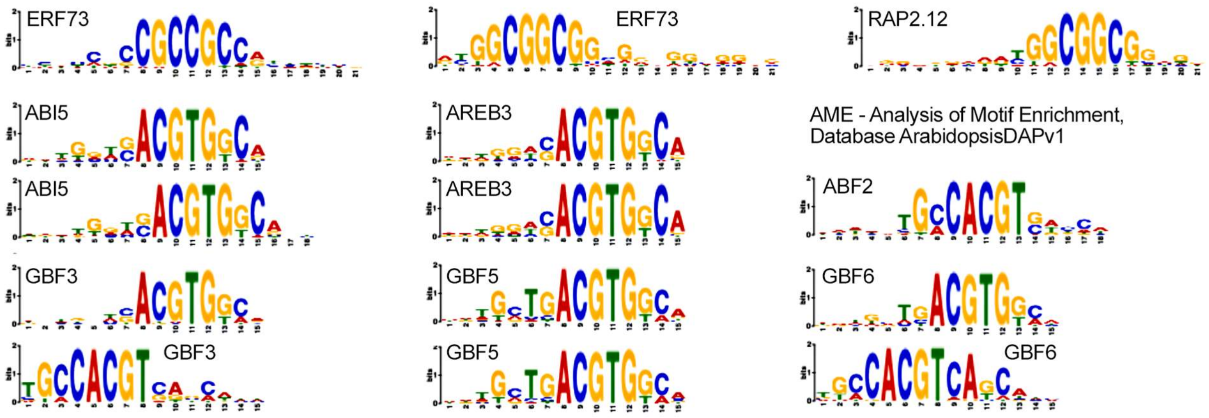

Supplemental Figure S13 continued next page…

B **Motif analyses of *Aethionema arabicum* and *Arabidopsis thaliana* ADH1 genes: detail analysis of proximal 1-kb 5'-regulatory regions**

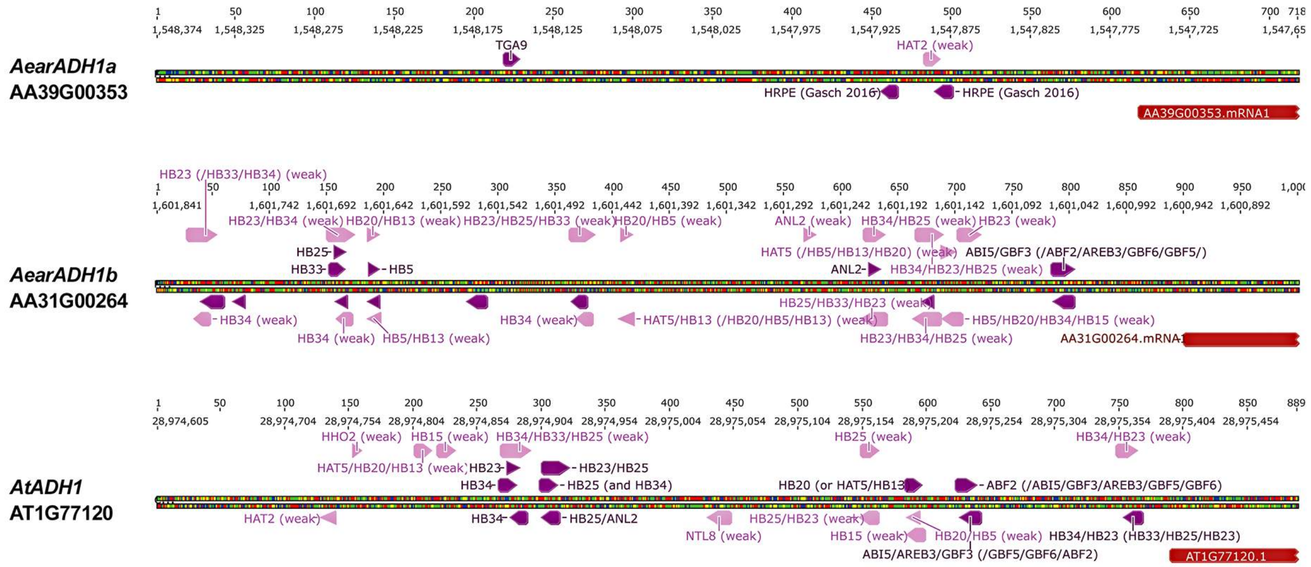

Motif analyses of ADH1 genes: ERF73 (red triangles) and HRPE (blue triangles) motif analysis of 3.5-kb regions

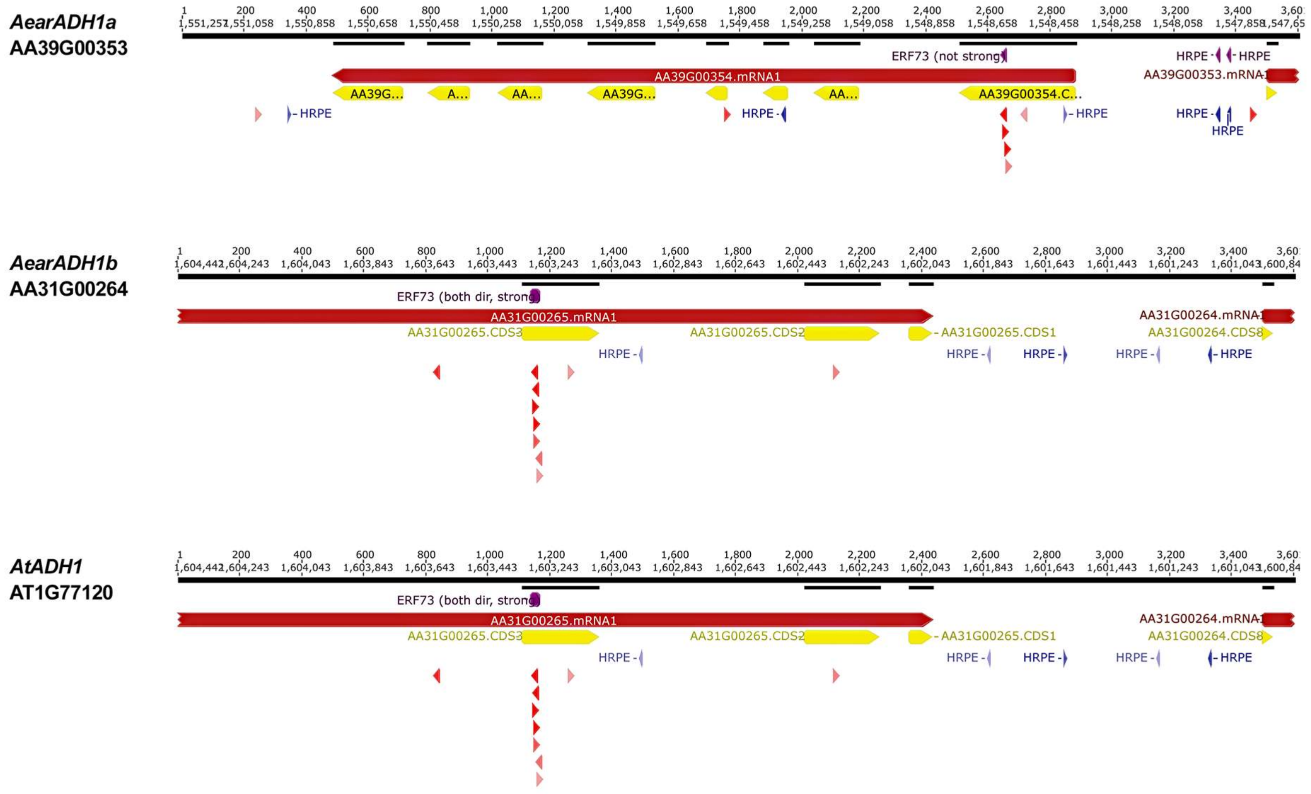

Supplemental Figure S13 continued next page…

C **Motif analyses of *Aethionema arabicum* and *Arabidopsis thaliana ERF73/71* genes: detail analysis of proximal 1-kb region**

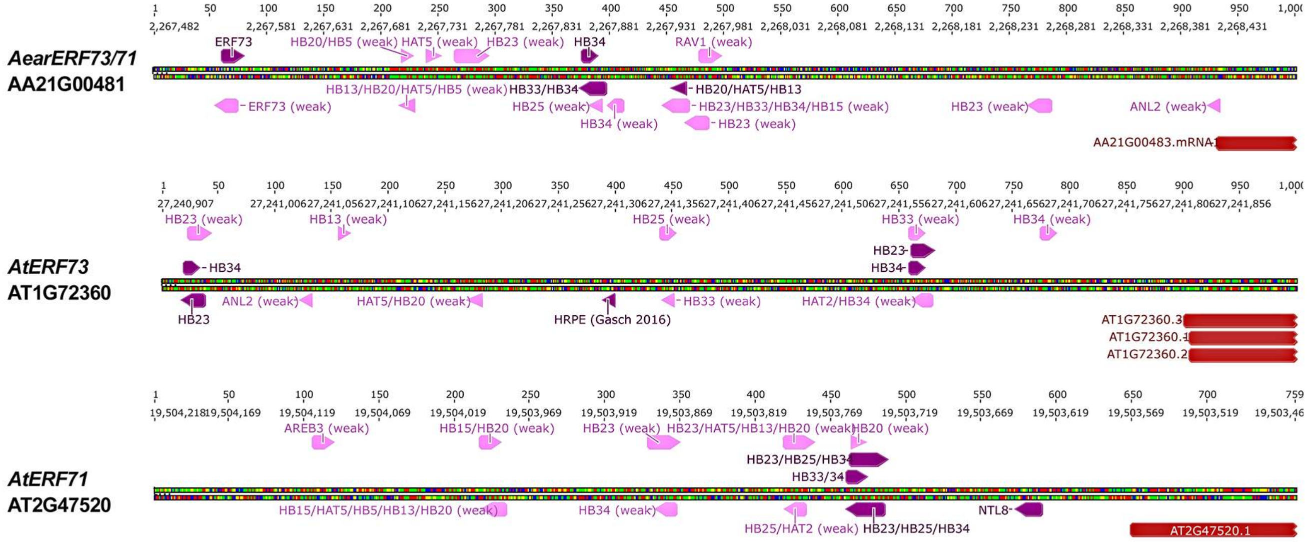

Motif analyses of *ERF73/71* genes: ERF73 (red triangles) and HRPE (blue triangles) motif analysis of 3.5-kb regions

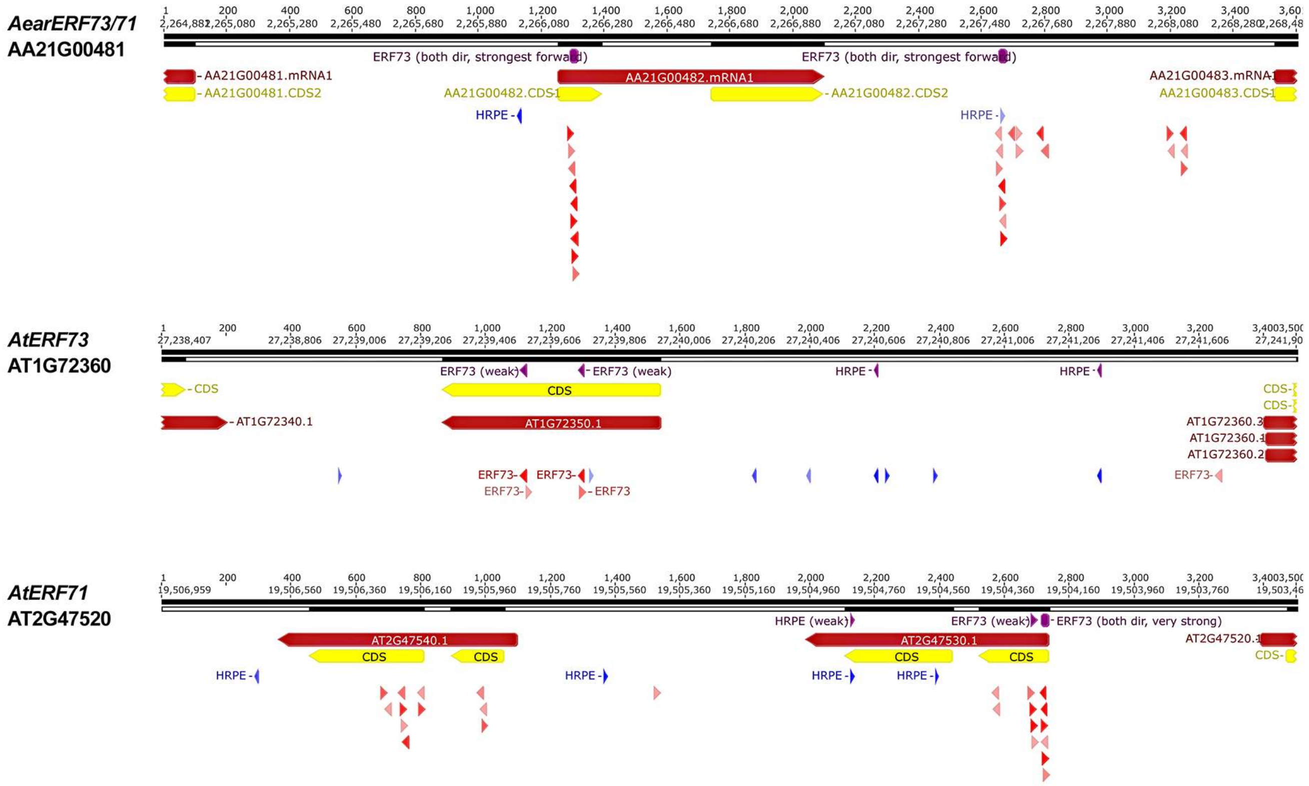

Supplemental Figure S13 continued next page…

D **Motif analyses of *Aethionema arabicum* and *Arabidopsis thaliana PDC* genes: detail analysis of proximal 1-kb regions**

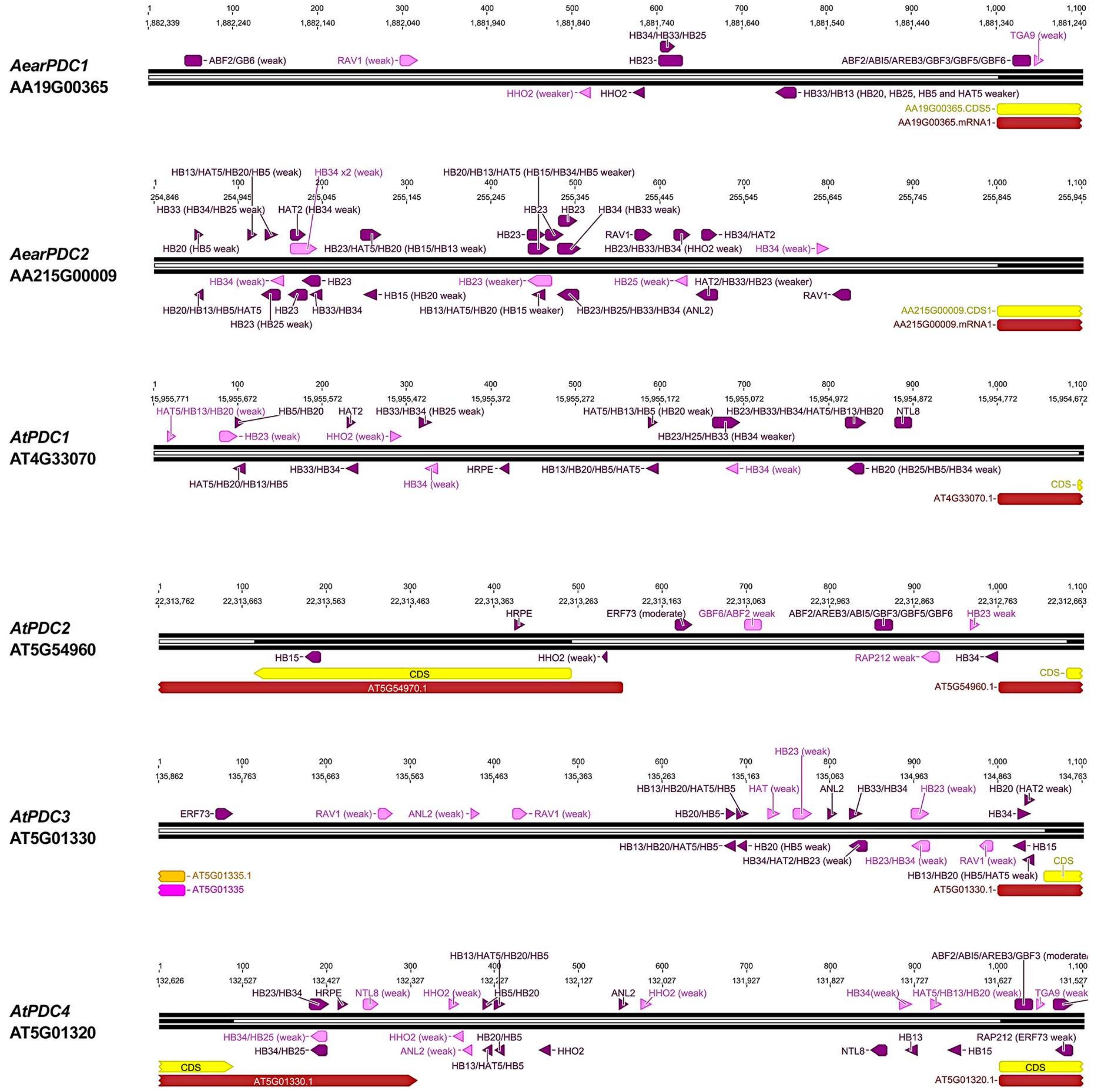

Supplemental Figure S13 continued next page…

E **Motif analyses of *PDC* genes: ERF73 (red triangles) and HRPE (blue triangles) motif analysis of 3.5-kb regions**

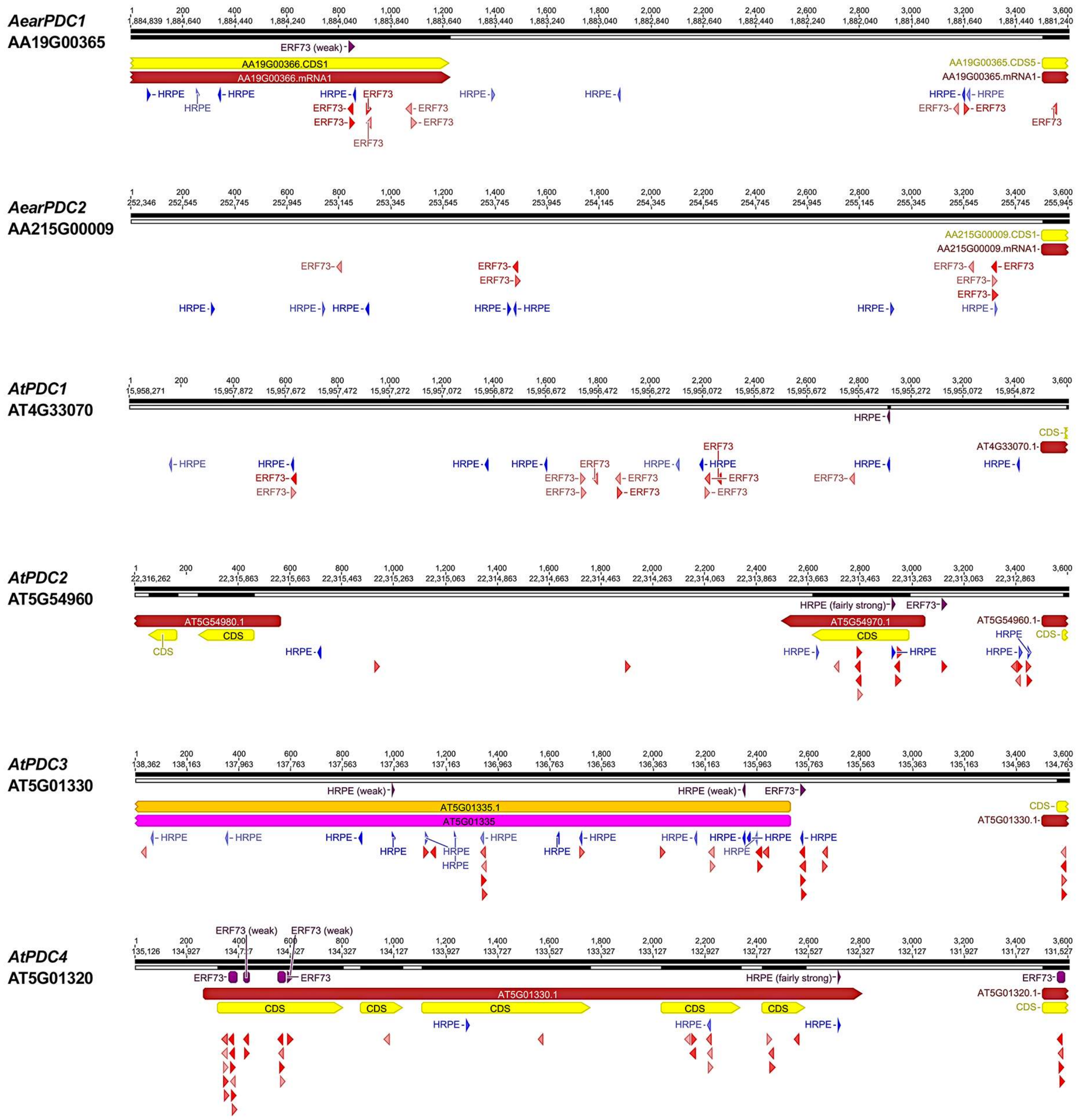

Supplemental Figure S13 continued next page…

F **Motif analyses of *Aethionema arabicum* and *Arabidopsis thaliana* LDH genes: detail analysis of proximal 1-kb regions**

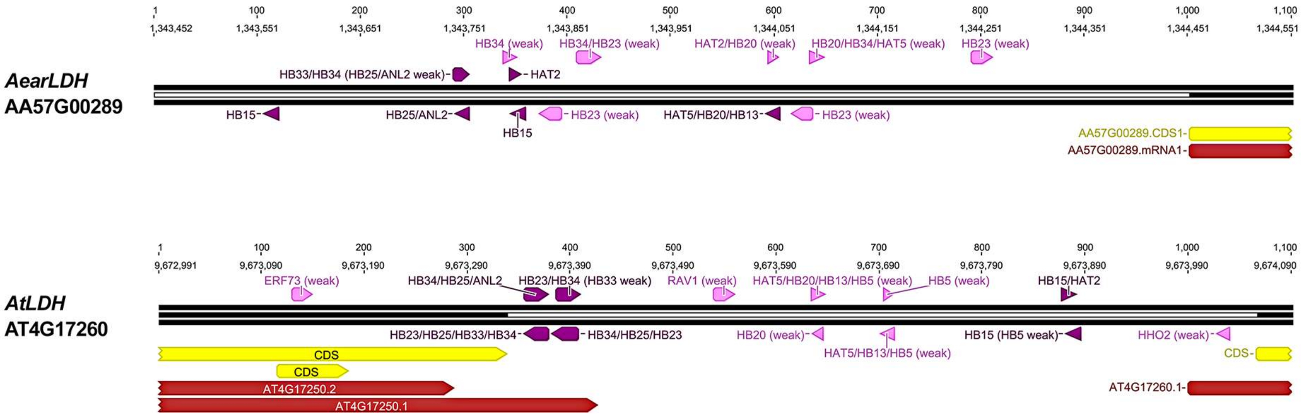

Promoter analyses of LDH genes: ERF73 (red triangles) and HRPE (blue triangles) motif analysis of 3.5-kb regions

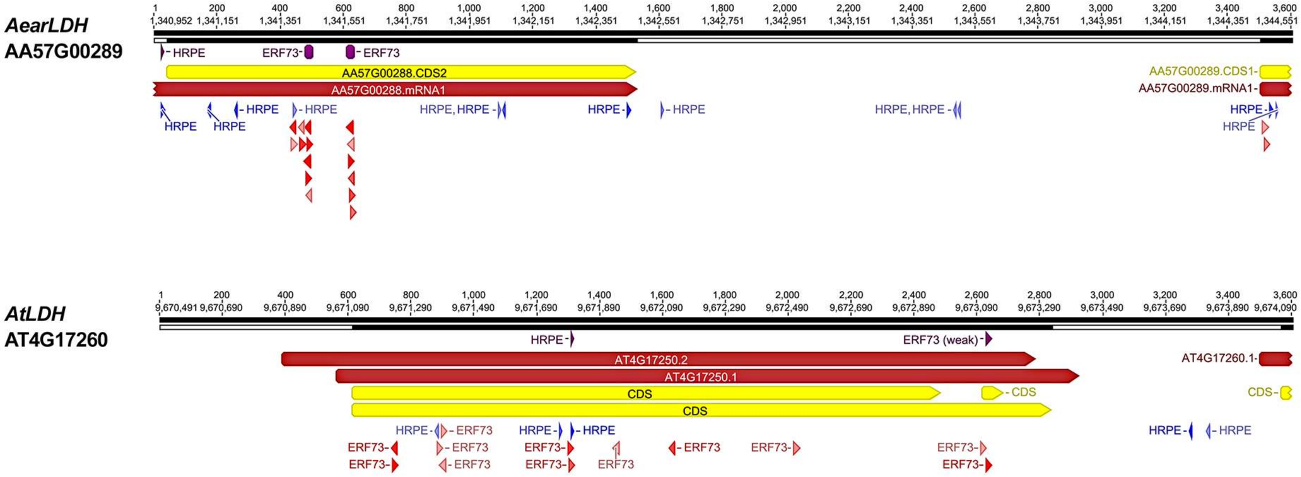

Supplemental Figure S13 continued next page…

G **Motif analyses of *Aethionema arabicum* and *Arabidopsis thaliana DOG1* genes: detail analysis of proximal 1-kb regions**

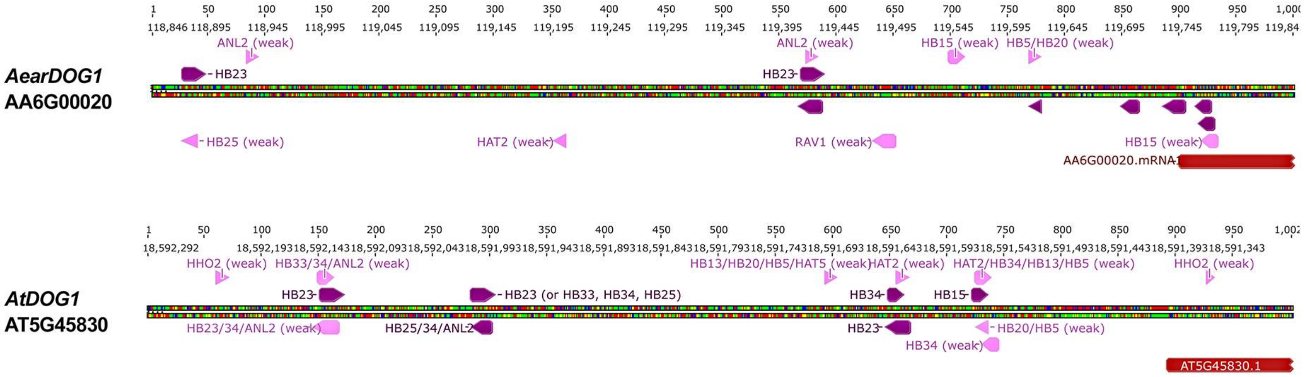

Motif analyses of *DOG1* genes: ERF73 (red triangles) and HRPE (blue triangles) motif analysis of 3.5-kb regions

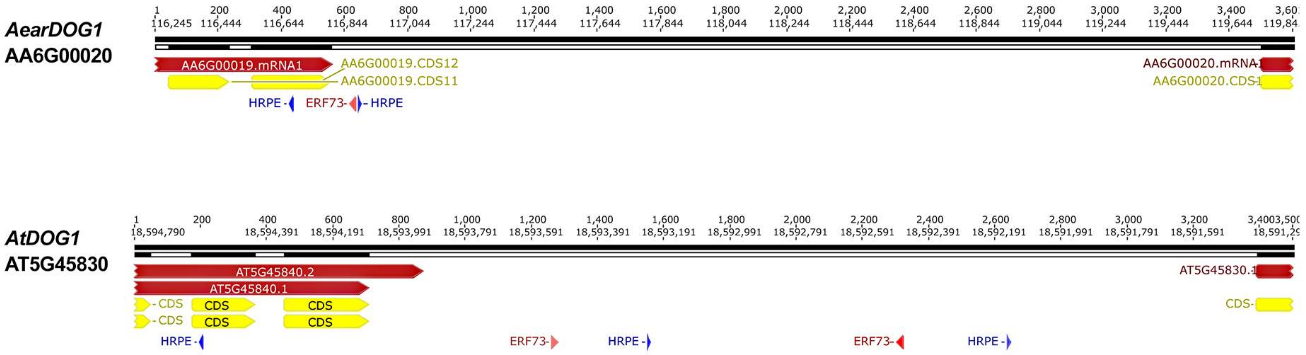

Supplemental Figure S13. Comparison of *Aethionema arabicum* and *Arabidopsis thaliana* ADH, PDC, ERF73/71, LDH and DOG1 gene 5'-regulatory regions for hypoxia and ABA-related *cis*-regulatory motifs (Supports Figure 7). A, Best possible hits for motifs used in FIMO (Grant et al., 2011) scans of promoter sequences. All motifs except one (HRPE) were from the ArabidopsisDAPv1 database (O'Malley et al., 2016) obtained from The MEME Suite (https://meme-suite.org/meme/doc/download.html) (Bailey et al., 2015). HRPE (also known as C9-motif from Gasch et al., 2016, Supplemental Data Set 2) was converted to MEME motif format. Sequence logos show similarity of ERF73 and RAP2.12 motifs, and similarity of the ABI5, AREB3, ABF2, and GBF motifs. FIMO was used to scan for all above motifs in the start codon -1000 bp to +100 bp ('1kb') and for HRPE and ERF73 motifs in -3.5 kb (-3.4 kb for *A. thaliana*) to +100 bp regions ('3.5kb') of: B, *ADH1* homologs *AearADH1a* (AA39G00353) and *AearADH1b* (AA31G00264) and *AtADH1* (AT1G77120); C, *AtERF71* (AT2G47520), *AtERF73* (AT1G72360) and homolog *AearERF73/71* (AA21G00481); *PDC* homologs *AearPDC1* (AA19G00365), *AearPDC2* (AA215G00009), *AtPDC1* (AT4G33070), *AtPDC2* (AT5G54960), *AtPDC3* (AT5G01330) and *AtPDC4* (AT5G01320) for D, 1kb regions and E, 3.5kb regions; F, *LDH* homologs *AearLDH* (AA57G00289), *AtLDH* (AT4G17260) and G, *DOG1* homologs *AearDOG1* (AA6G00020) and *AtDOG1* (AT5G45830). Identified motifs were shaded and annotated according to their p-value. Strong hits are shaded darker and weak hits shaded lighter. Hits subjectively determined to be weaker relative to other hits for a given sequence were labelled as such. p-values of <1E-4 and <1E-3 were considered as hits for the 1kb and 3.5 kb FIMO scans, respectively. Detailed output for hits is given in Supplemental Data Set S4.

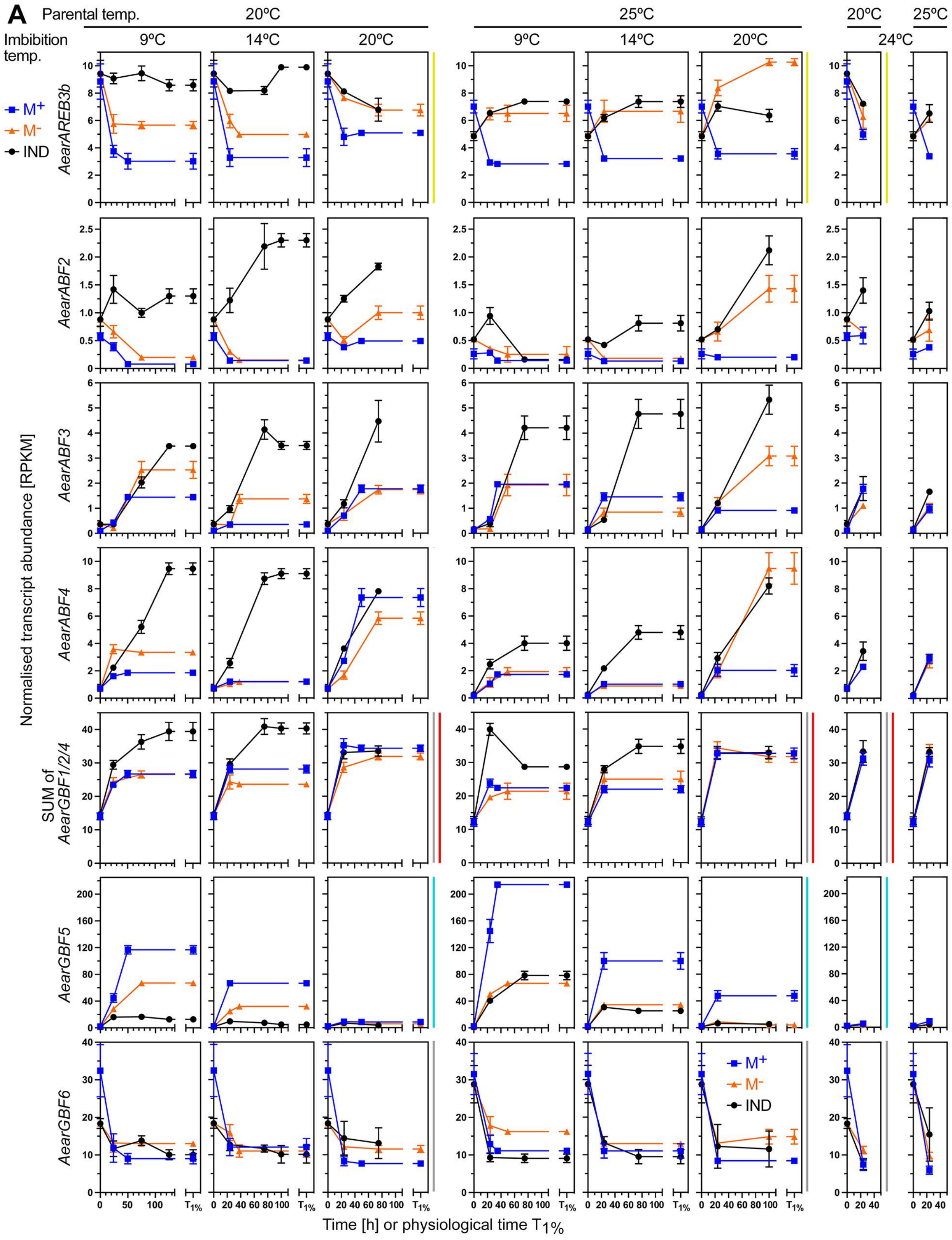

Supplemental Figure S14 continued next page…

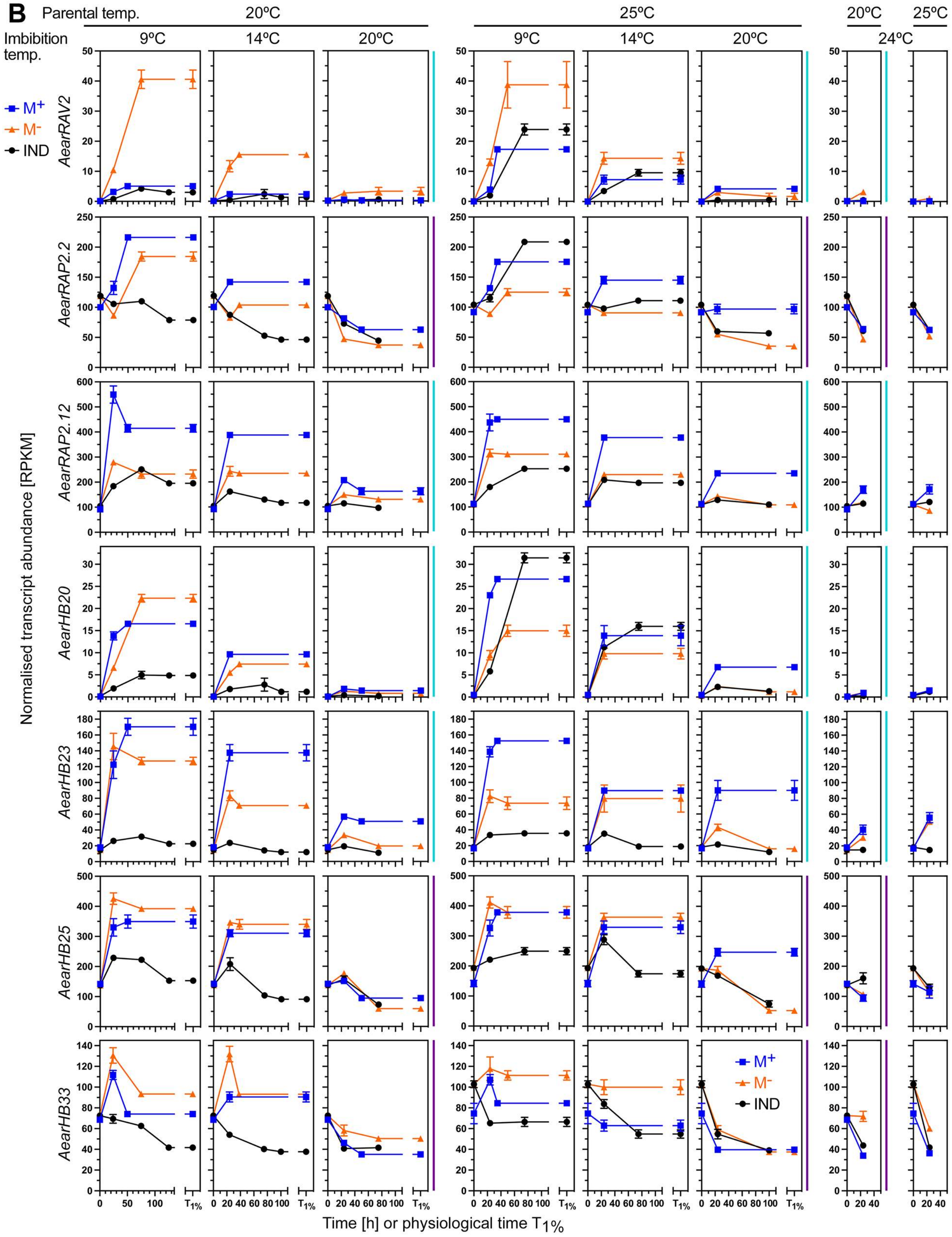

Supplemental Figure S14 continued next page…

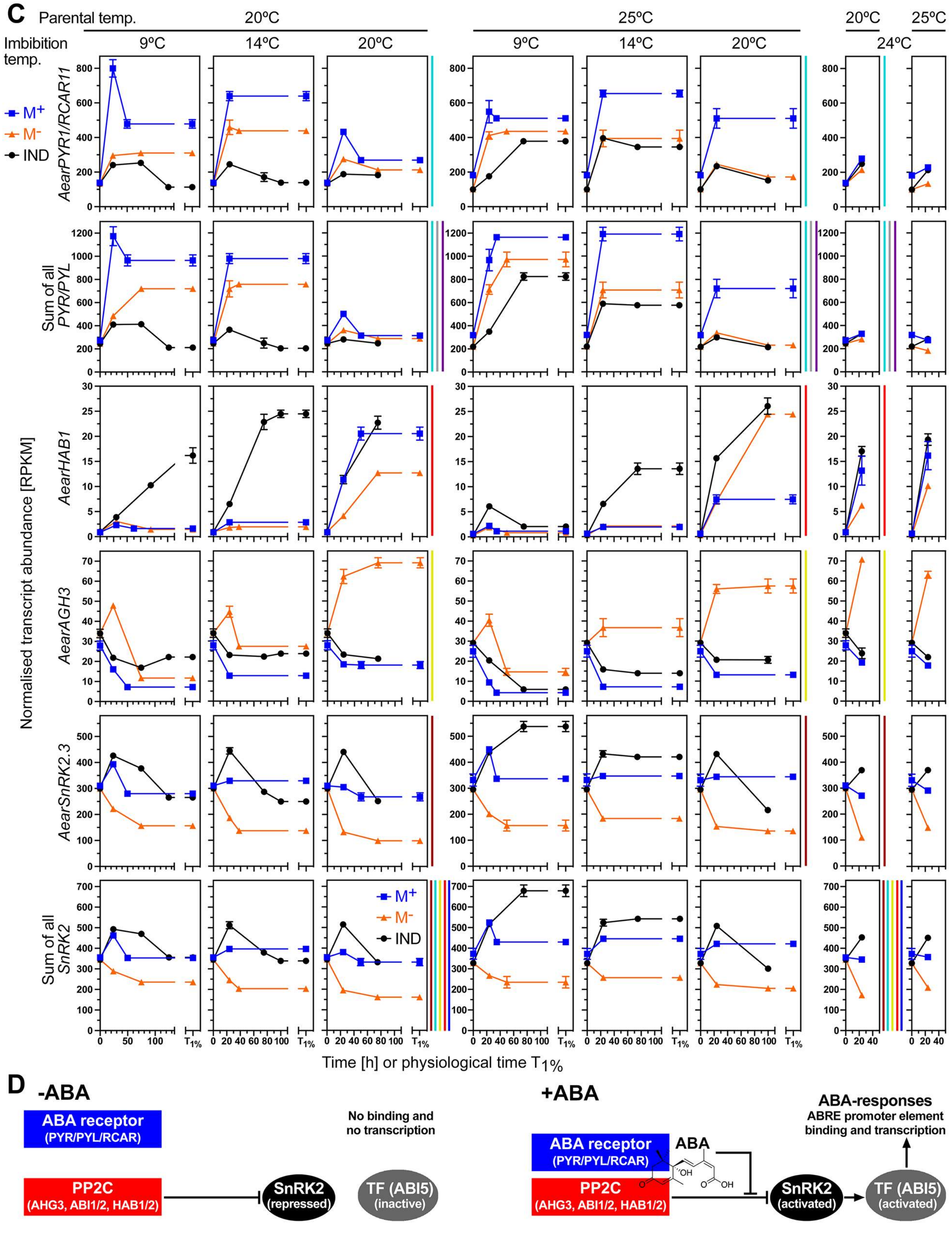

Supplemental Figure S14. Expression patterns of *Aethionema arabicum* ABA-related genes (This supports Figure 9). Transcript abundance patterns (RNA-seq) of *Ae. arabicum* genes in seeds of imbibed dimorphic diaspores (M^{+} seeds, IND fruits) and bare M^{-} seeds (extracted from IND fruits) from two maternal temperature regimes (20ºC versus 25ºC) at four different imbibition temperatures (9, 14,

20 and 24ºC). WGCNA modules (Figure 3) for these genes are indicated by the vertical color lines next to the graphs. Mean ± SEM values of 3 replicates each with 60-80 seeds are presented. A, ABA-related bZIP TF TFs binding to G-box and ABA-responsive element (ABRE) motifs, including ABF (ABRE-binding factors), AREB3 (ABRE-binding proteins), and GBF (G-box-binding factors). B, ABA and hypoxia related AP2/EREBP and homeobox (HB) TFs including RAV (Related to ABI3/VP1), RAP (HRPE-binding ERF-VII TF). In contrast to *AearABI5*, *AearGBF1-4*, and *AearAREB/ABF* TF genes, which were higher expressed in IND fruits, the *AearGBF5*, *AearHB13*, *AearHB20*, *AearHB23*, *AearHB25*, *AearHB33*, *AearRAP2.2*, *AearRAP2.12,* and *AearRAV2* TF genes were generally higher expressed in bare M^- seeds and in M^+ seeds. In *A. thaliana,* the HB13 and HB20 TFs constitute node-regulators within the co-expression network controlling seed-to-seedling phase transition (Silva et al., 2016) while other HB TFs control seed ABA sensitivity, dormancy, longevity and embryo growth (Barrero et al., 2010; Wang et al., 2011; Bueso et al., 2014; Stamm et al., 2017; Renard et al., 2021). The RAV (Related to ABI3/VP1) TFs are involved in ABA and stress responses of seeds, and the ERF-VII RAP2 TFs are known to be involved in sustaining ABA responses during hypoxia of *A. thaliana* seedlings (Papdi et al., 2015; Gasch et al., 2016). C; ABA receptors (PYR1/RCAR11, PYR/PLY/RCAR) and signaling components including protein phosphatase 2C proteins (e.g., HAB1 and AGH3) and SNF1-related protein kinase subfamily 2 (SnRK2) which has ABI5 and other ABA-related TFs as targets (Nambara et al., 2010). Transcript abundance patterns of these ABA signaling components also exhibit pericarp-affected expression patterns in the *Ae. arabicum* morphs. D, Simplified scheme of ABA perception and signaling, for details see Nambara et al. (2010). For *Ae. arabicum* gene names and IDs see Supplemental Table S2 or the Gene Expression Atlas (https://plantcode.cup.uni-freiburg.de/easy_gdb/tools/expression/expression_input.php); for RNAseq single values see the Expression Atlas or Supplemental Data Set 1. For gene IDs from and modules of the presented expression results (individual or as cumulative sum) see Supplemental Table S2; included PYR/PYL/RCAR genes and modules are listed here: AA57G00057 (PYR1/RCAR11, turquoise), AA11G00152 (grey), AA1G00061, AA29G00245 (turquoise), AA31G00648, AA31G00648, AA32G00133, AA32G01060 (purple), AA35G00054 (grey), AA56G00008 (turquoise), AA57G00145, AA61G00520, AA6G00016 (yellow), AA805G00002 (turquoise).

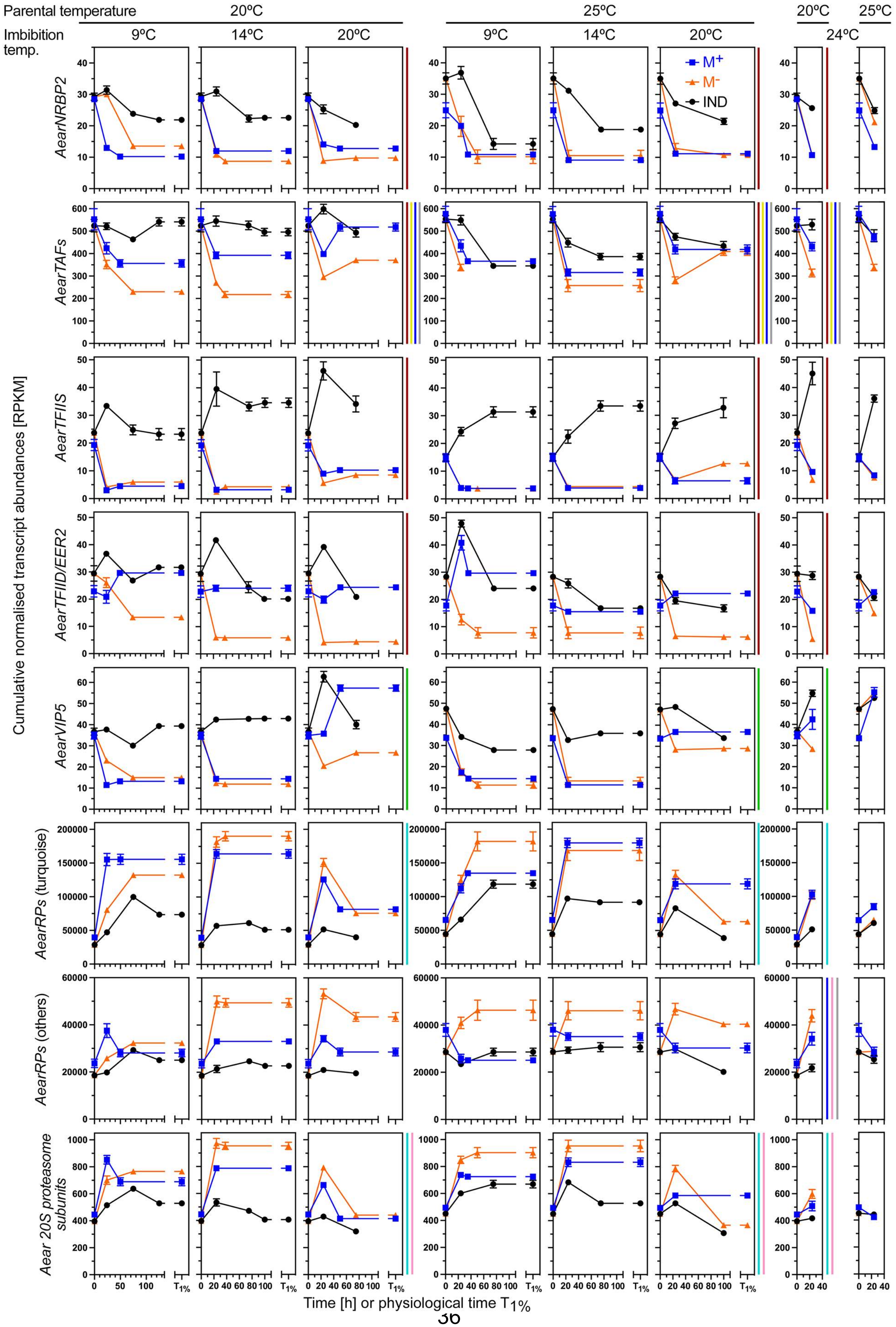

Parental temperature
20ºC
25ºC
20ºC
25ºC
Imbibition temp.
9ºC
14ºC
20ºC
9ºC
14ºC
20ºC
24ºC
M+
M-
IND
AearNRBP2
AearTAFs
AearTFIIS
AearTFIID/EER2
AearVIP5
AearRPs (turquoise)
AearRPs (others)
Aear 20S proteasome subunits
Cumulative normalised transcript abundances [RPKM]
Time [h] or physiological time T1%

Supplemental Figure S15. Expression patterns of *Aethionema arabicum* general transcription and translation related genes (Supports Figure 9). This includes the general RNA polymerase II transcription elongation complex (Antosz et al., 2017), ribosomal proteins (RPs) and 20S proteasome subunits. For details see Wilhelmsson et al. (2019). Transcript abundance patterns (RNA-seq) of *Ae. arabicum* genes in seeds of imbibed dimorphic diaspores (M^+ seeds, IND fruits) and M^- seeds (extracted from IND fruits) from two maternal temperature regimes (20ºC versus 25ºC) at four different imbibition temperatures (9, 14, 20 and 24ºC). WGCNA modules (Figure 3) for these genes are indicated by the vertical color lines next to the graphs. Mean ± SEM values of 3 replicates each with 60-80 seeds are presented. For *Ae. arabicum* gene names and IDs see the Gene Expression Atlas (https://plantcode.cup.uni-freiburg.de/easy_gdb/tools/expression/expression_input.php) for: NRBP2 (DNA-directed RNA polymerase protein, AA57G00370), cumulative TAF sum (TBP-associated factors: AA19G00069, AA46G00184, AA7G00014, AA93G00180, AA31G00549, AA3G00252, AA6G00143, AA102G00330, AA12G00070, AA15G00183, AA118G00072, AA15G00168, AA2G00137, AA33G00098), cumulative TFIIS sum (AA139G00004, AA44G00247, AA35G00003, AA93G00180, AA53G00202, AA9G00120, AA53G00203), TFIID/EER2 (AA31G00674), PAF-C/VIP5 (vernalization independence5, AA53G00526), cumulative 20S proteasome subunits sum (AA32G00927, AA326G00002, AA3G00223, AA87G00203, AA37G00101, AA31G00785, AA17G00024, AA26G00153, AA87G00239), cumulative RP sum (ribosomal proteins of the turquoise (*RPs) and grey/pink/blue/purple modules (**RPs):

***RPs:** AA101G00012, AA102G00014, AA102G00094, AA102G00243, AA10G00061, AA10G00080, AA10G00130, AA10G00137, AA10G00174, AA10G00246, AA10G00354, AA10G00397, AA116G00015, AA1312G00001, AA157G00010, AA15G00029, AA15G00304, AA162G00004, AA1679G00001, AA17G00034, AA17G00041, AA18G00038, AA18G00042, AA18G00170, AA19G00027, AA19G00104, AA19G00122, AA19G00206, AA19G00268, AA19G00272, AA19G00282, AA19G00299, AA19G00300, AA1G00040, AA20G00024, AA21G00117, AA21G00126, AA21G00142, AA21G00422, AA21G00486, AA21G00490, AA226G00005, AA22G00082, AA23G00037, AA23G00103, AA252G00005, AA25G00051, AA26G00029, AA26G00105, AA26G00107, AA26G00174, AA26G00228, AA26G00313, AA26G00402, AA26G00541, AA279G00004, AA29G00062, AA29G00097, AA29G00120, AA29G00136, AA29G00278, AA2G00051, AA2G00074, AA30G00077, AA30G00151, AA30G00277, AA30G00351, AA311G00008, AA3165G00002, AA31G00179, AA31G00196, AA31G00518, AA31G00537, AA31G00545, AA31G00733, AA31G00868, AA32G00184, AA32G00418, AA32G00452, AA32G00474, AA32G00577, AA32G00856, AA32G00913, AA32G01182, AA337G00005, AA33G00214, AA33G00250, AA33G00253, AA34G00018, AA34G00041, AA35G00035, AA35G00079, AA36G00021, AA36G00061, AA37G00154, AA38G00037, AA38G00038, AA39G00034, AA39G00138, AA39G00156, AA39G00380, AA39G00525, AA39G00536, AA39G00618, AA39G00628, AA39G00709, AA39G00764, AA3G00049, AA3G00100, AA3G00155, AA3G00197, AA40G00048, AA40G00110, AA40G00119, AA40G00120, AA40G00125, AA40G00138, AA40G00490, AA40G00566, AA40G00643, AA41G00015, AA44G00144, AA44G00294, AA44G00425, AA44G00509, AA44G00549, AA44G00550, AA44G00659, AA45G00025, AA46G00082, AA48G00034, AA4G00202, AA4G00258, AA52G00021, AA53G00041, AA53G00289, AA53G00416, AA53G00609, AA53G00917, AA53G00945, AA53G01026, AA53G01078, AA53G01298, AA53G01346, AA54G00080, AA54G00146, AA54G00200, AA54G00208, AA54G00274, AA54G00294, AA54G00404, AA56G00034, AA57G00082, AA57G00225, AA58G00014, AA598G00001, AA5G00068, AA5G00080, AA5G00081, AA5G00168, AA5G00254, AA60G00122, AA60G00131, AA60G00190, AA60G00274, AA61G00059, AA61G00106, AA61G00154, AA61G00286, AA61G00291, AA61G00341, AA61G00453, AA61G00469, AA61G00604, AA634G00006, AA63G00010, AA63G00014, AA63G00040, AA64G00014, AA69G00034, AA6G00024, AA6G00038, AA70G00001, AA71G00017, AA77G00006, AA7G00019, AA7G00025, AA87G00051, AA87G00166, AA87G00216, AA87G00291, AA89G00010, AA8G00038, AA8G00132, AA8G00148, AA8G00265, AA8G00319, AA90G00024, AA90G00045, AA93G00112, AA93G00120, AA93G00125, AA96G00014, AA96G00070, AA96G00143, AA96G00153, AA9G00044, AA9G00101, AA9G00158, AA9G00170.

****RPs:** AA10G00010, AA10G00387, AA11G00002, AA11G00055, AA14G00024, AA16G00060, AA16G00071, AA21G00173, AA21G00221, AA21G00348, AA21G00412, AA25G00056, AA26G00495, AA26G00566, AA26G00692, AA31G00860, AA32G00227, AA33G00249, AA33G00251, AA33G00279, AA40G00523, AA40G00524, AA44G00242, AA44G00717, AA44G00719, AA45G00019, AA4G00198, AA52G00039, AA53G01352, AA56G00064, AA61G00345, AA65G00313, AA78G00023, AA87G00117, AA88G00003, AA93G00183, AA9G00203, AA104G00013, AA123G00016, AA18G00076, AA18G00235, AA1976G00001, AA21G00091, AA21G00273, AA29G00007, AA31G00096, AA31G00455, AA32G00110, AA32G00256, AA32G00442, AA32G00581, AA32G00921, AA32G01176, AA33G00107, AA33G00137, AA34G00040, AA37G00047, AA37G00051, AA38G00113, AA40G00013, AA447G00006, AA44G00057, AA4G00096, AA53G00830, AA54G00092, AA60G00009, AA60G00061, AA61G00683, AA72G00011, AA78G00038, AA7G00077, AA87G00075, AA10G00188, AA119G00004, AA131G00038, AA14G00034, AA18G00011, AA19G00483, AA24G00028, AA26G00426, AA30G00072, AA30G00134, AA31G00420, AA31G00703, AA31G00855, AA32G00542, AA32G00843, AA32G01181, AA33G00099, AA37G00041, AA38G00070, AA39G00471, AA40G00277, AA44G00379, AA45G00031, AA4G00252, AA53G00417, AA57G00013, AA60G00181, AA65G00020, AA6G00134, AA87G00134, AA94G00008, AA39G00366.

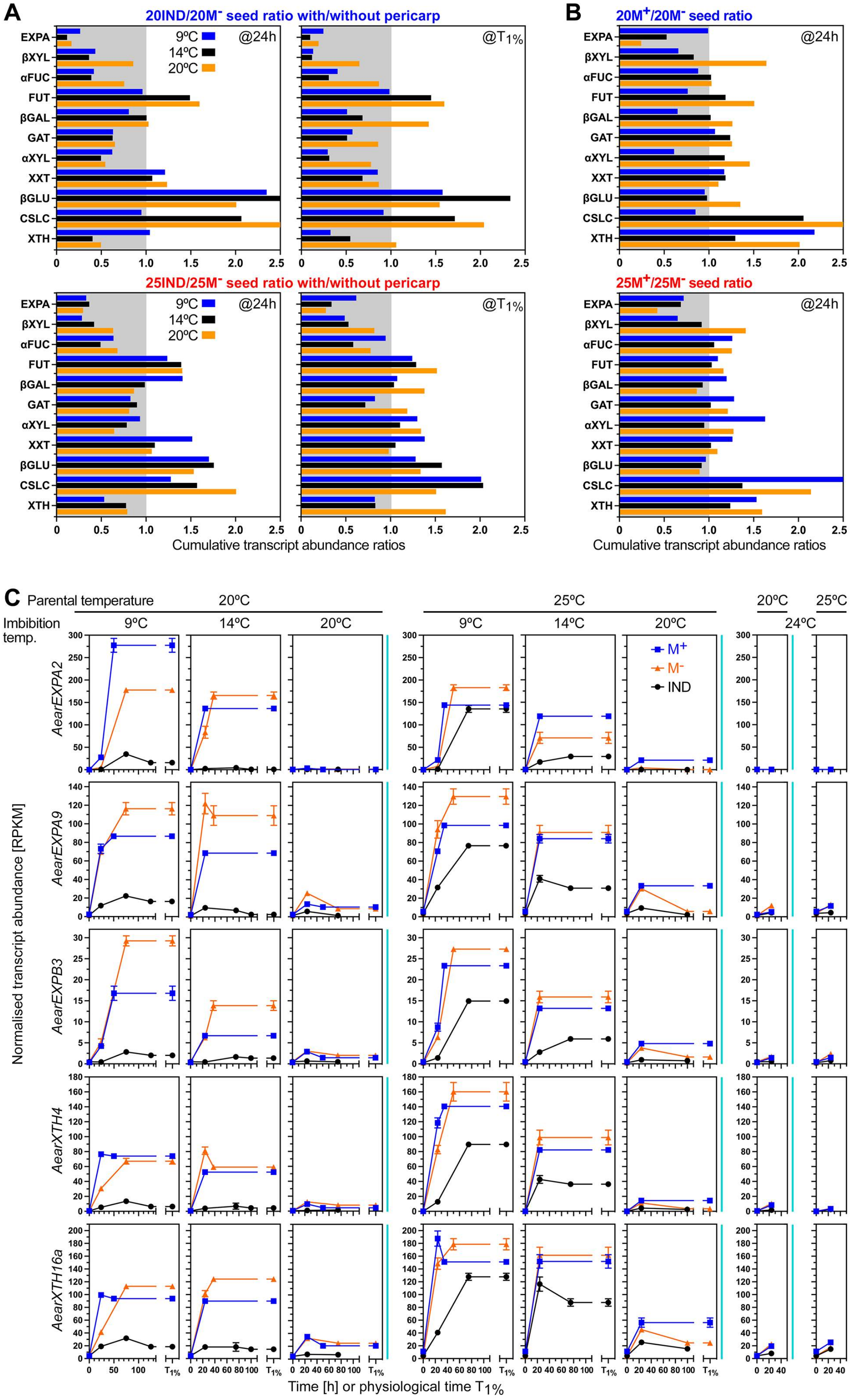

Supplemental Figure S16 continued next page…

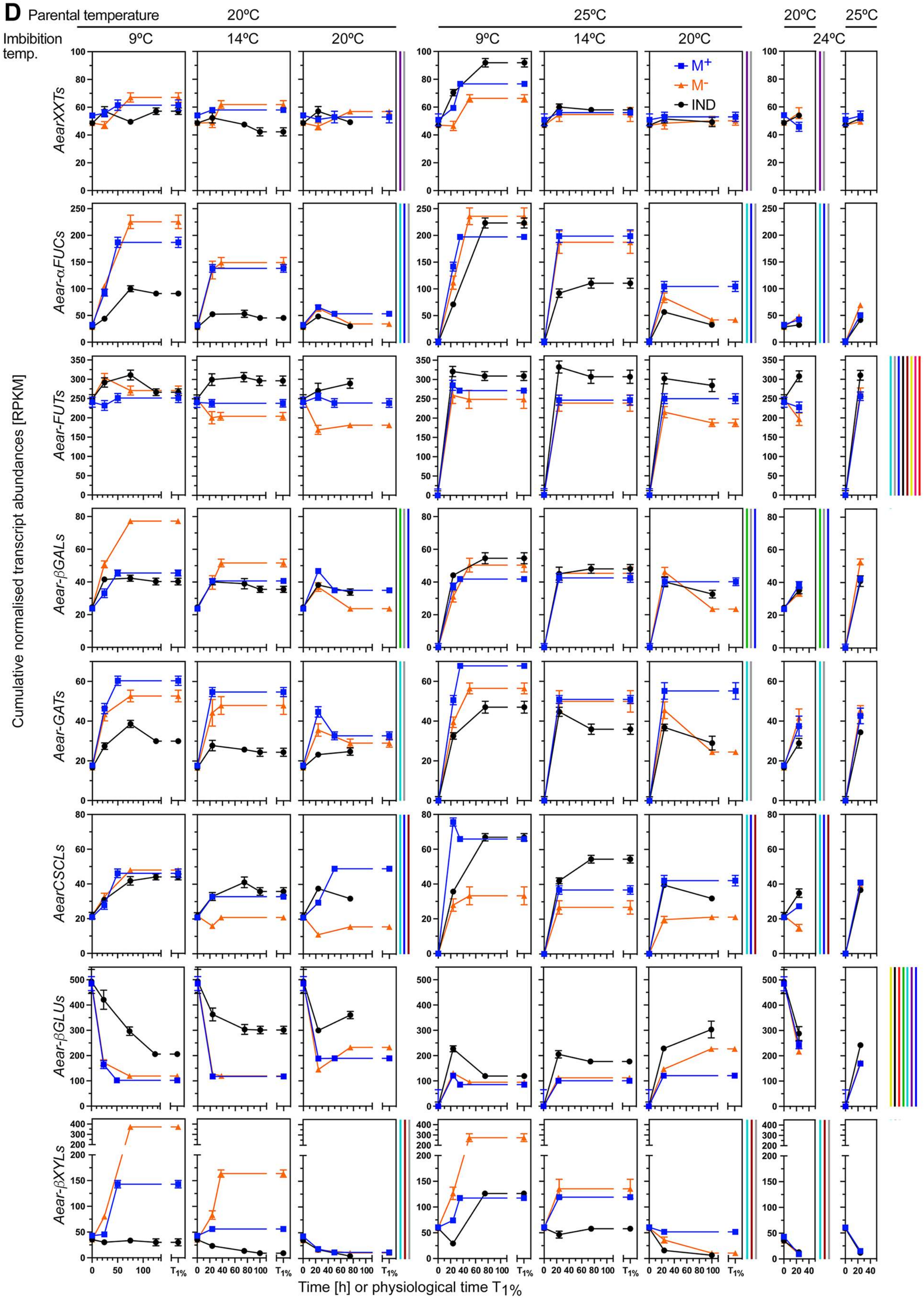

Supplemental Figure S16. Expression patterns of *Aethionema arabicum* cell wall remodeling protein genes with focus on expansins and enzymes modifying xyloglucan (supports Figure 10). Transcript

abundance patterns (RNA-seq) in seeds of imbibed dimorphic diaspores (M^+ seeds, IND fruits) and bare M^- seeds (extracted from IND fruits) from two maternal temperature regimes (20ºC versus 25ºC) at four different imbibition temperatures (9, 14, 20 and 24ºC). WGCNA modules (Figure 3) for these genes are indicated by the vertical color lines next to the graphs. Mean ± SEM values of 3 replicates each with 60-80 seeds are presented. A and B, Effect of the pericarp on the expression ratios of expansin and xyloglucan-related cell-wall remodeling protein genes in the M^- seeds of imbibed IND fruits and isolated M^- seeds at T1% (A) and at 24 h (B). C, Transcript abundance patterns of *Ae. arabicum* expansins (EXP) and xyloglucan *endo*-transglycoylases/hydrolases (XTH). D, Transcript abundance patterns of *Ae. arabicum* xyloglucan modifying enzymes: α-xylosyltransferase (XXT), α-fucosidase (FUC), α-fucosyltransferase (FUT), β-galactosidase (βGAL), α-galactosyltransferase (GAT), glucan synthase (CSCL), β-glucosidase (βGLU). For *Ae. arabicum* gene names/IDs see lists or the Expression Atlas (https://plantcode.cup.uni-freiburg.de/easy_gdb/tools/expression/expression_input.php); for RNAseq single values see the Expression Atlas or Supplemental Data Set S1. Symbols, gene IDs and modules of these genes are listed below:

XXT	AA31G00510	purple	βGAL	AA18G00007	
XXT	AA32G00279	grey	βGAL	AA26G00093	
XXT	AA39G00160		βGAL	AA30G00144	
XXT	AA61G00614		βGAL	AA31G00649	
XXT	AA65G00104		βGAL	AA32G00498	green
αFUC	AA118G00010	turquoise	βGAL	AA32G00867	grey
αFUC	AA118G00011	turquoise	βGAL	AA44G00698	
αFUC	AA118G00012	blue	βGAL	AA4G00117	
αFUC	AA19G00339		βGAL	AA4G00118	blue
αFUC	AA32G00891	grey	GAT	AA21G00082	
αFUC	AA901G00001	turquoise	GAT	AA57G00452	turquoise
αFUC	AA93G00092		GAT	AA5G00089	grey
FUT	AA10G00221	magenta	GAT	AA87G00320	turquoise
FUT	AA14G00009	blue	CSCL	AA102G00313	
FUT	AA15G00140		CSCL	AA29G00116	blue
FUT	AA19G00061	grey	CSCL	AA53G01041	turquoise
FUT	AA21G00205	turquoise	CSCL	AA627G00001	brown
FUT	AA26G00281	blue	βGLU	AA10G00241	yellow
FUT	AA26G00769	turquoise	βGLU	AA11G00006	black
FUT	AA2G00219	turquoise	βGLU	AA12G00212	turquoise
FUT	AA31G00030	red	βGLU	AA26G00479	red
FUT	AA39G00026	turquoise	βGLU	AA26G00787	yellow
FUT	AA39G00435		βGLU	AA44G00262	
FUT	AA40G00525		βGLU	AA45G00011	green
FUT	AA43G00095		βGLU	AA61G00378	
FUT	AA4G00195	red	βGLU	AA62G00052	
FUT	AA4G00273	brown	βGLU	AA78G00002	purple
FUT	AA53G00296	grey	βGLU	AA78G00006	green
FUT	AA53G00630	brown	βGLU	AA8G00196	blue
FUT	AA53G00855	grey	βXYL	AA44G00238	
FUT	AA53G01435	yellow	βXYL	AA44G00321	grey
FUT	AA54G00100		βXYL	AA58G00011	turquoise
FUT	AA54G00344		βXYL	AA7G00012	turquoise
FUT	AA57G00016	black	EXPA2	AA35G00022	turquoise
FUT	AA586G00001	magenta	EXPA9	AA19G00314	turquoise
FUT	AA946G00002	grey	EXP3	AA228G00009	turquoise
FUT	AA9G00083		XTH4	AA110G00001	turquoise
αXYL	AA32G01046	turquoise	XTH16a	AA78G00036	turquoise

Supplemental Table S1. *Aethionema arabicum* seed and fruit harvest results: seed and fruit mass, amounts and ratios of each harvested from plants grown at two temperatures.

Maternal temperature (ºC)	Seed/ fruit	mass (mg)[1]	Harvested (g)	Seed number	Fruit number[2]	Seed mass (%)	Diaspore mass (%)	Diaspore number (%)	Fruit number (%)
20	M+	0.3355	72.0	214605	N/A	69.6	37.3	65.1	N/A
	M-	0.273	31.4	114938	N/A	30.4	N/A	N/A	N/A
	DEH	-	-	N/A	53651	N/A	N/A	N/A	31.8
	IND	1.0535	121.1	N/A	114938	N/A	62.7	34.9	68.2
25	M+	0.378	50.0	132275	N/A	87.0	67.9	83.7	N/A
	M-	0.2894	7.5	25847	N/A	13.0	N/A	N/A	N/A
	DEH	-	-	N/A	33069	N/A	N/A	N/A	56.1
	IND	0.9161	23.7	N/A	25847	N/A	32.1	16.3	43.9

[1] Seed mass based on 8 × 100 seed / fruit weights
[2] Fruit number for DEH fruits based on approximation of an average of 4 M^+ seeds per DEH fruit.

Supplemental Table S2. *Aethionema arabicum* gene names and IDs and modules (Figure 3) for the presented expression results in Figures 5, 7, 8, 9, 10 and Supplemental Figures S5, S8, S10, S11, and S12. For further details about individual genes and gene IDs and link to the v3.1 genome (Fernandez-Pozo et al., 2021) see the Gene Expression Atlas (https://plantcode.cup.uni-freiburg.de/easy_gdb/tools/expression/expression_input.php).

Figure	Symbol	*Aethionema arabicum* gene ID (v2.5 genome)	Module
5, 8, S5	NCED6	AA78G00012	green
5, 8, S5	CYP707A3a	AA6G00060	blue
5, 8	ABCG40	AA37G00179	brown
5, 8	DOG1	AA6G00020	
S5	ZEP/ABA1	AA8G00025	brown
S5	NCED2	AA57G00116	
S5	NCED3a	AA26G00165	
S5	NCED3b	AA15G00127	
S5	NCED5	AA54G00417	grey
S5	NCED9	AA31G00716	grey
S5	ABA2/SDR1	AA32G01008	grey
S5	AAO	AA8G00200	yellow
S5	AAO	AA8G00197	
S5	AAO	AA19G00094	
S5	AAO	AA32G00943	brown
S5	CYP707A3b	AA57G00196	
S5	CYP707A2	AA32G00787	grey
S5	CYP707A4	AA26G00741	
S5	CYP707A4	AA26G00600	
S5	GA3OX1	AA37G00176	turqoiuse
S5	GA3OX2	AA31G00895	purple
S5	GA3OX4	AA31G00896	
S5	GA2OX1	AA31G00720	
S5	GA2OX2	AA54G00411	grey
S5	GA2OX3	AA18G00108	
S5	GA2OX4	AA18G00203	
S5	GA2OX6	AA7G00001	
S5	GA2OX7	AA2G00197	
S5	GA2OX8	AA57G00320	
7, 8	ERF71/73 (HRE1/2)	AA21G00481	grey
7, 8	ADH1a	AA39G00353	brown
7	ADH1b	AA31G00264	green
S9	PDC1	AA19G00365	
8, S9	PDC2	AA215G00009	yellow
S9	LDH	AA57G00289	yellow
S11	AspAT	AA46G00122	red
S11	AspAT-ASP1	AA32G00623	turquoise
S11	AspAT-ASP2	AA7G00130	grey

S11	AspAT-ASP3	AA44G00406	blue
S11	MDHc	AA19G00113	grey
S11	MDHc	AA32G00436	grey
S11	NADP-ME2	AA44G00419	grey
S12	NAC102	AA4G00115	brown
S12	HHO2	AA1304G00001	green
8, S12	HRA1	AA93G00176	grey
S12	PCO1	AA40G00576	grey
S12	PCO2	AA87G00177	grey
S12	ACO1	AA33G00241	turquoise
8, S12	ETR2	AA17G00111	blue
8, S12	JAZ3	AA26G00461	brown
9	ABI5	AA60G00170	green
9	AREB3a (ABF)	AA93G00015	green
S14	AREB3b (ABF)	AA61G00149	yellow
9	ABF1	AA2G00098	red
S14	ABF2	AA210G00005	
S14	ABF3	AA118G00053	
8, S14	ABF4	AA26G00601	
S14	GBF1	AA30G00183	red
8, 9	GBF3	AA21G00361	red
S14	GBF2	AA32G00122	red
S14	GBF4	AA19G00150	grey
S14	GBF5	AA33G00153	turquoise
S14	GBF6	AA45G00023	grey
S14	RAV2	AA39G00678	turquoise
S14	RAP2.2	AA26G00147	purple
S14	RAP2.12	AA15G00147	turquoise
8, S14	HAB1	AA31G00661	red
S14	AHG3	AA52G00006	yellow
S14	SNRK2.10/2B	AA53G00514	red
S14	SNRK2.3	AA8G00036	brown
S14	SNRK2.4	AA65G00151	yellow
S14	SNRK2.5	AA46G00035	red
S14	SNRK2.5	AA4G00103	turquoise
S14	SNRK2.7	AA44G00187	blue
9	HB13	AA38G00103	turquoise
S14	HB20	AA10G00418	turquoise
S14	HB23	AA9G00043	turquoise
S14	HB25	AA4G00267	purple
S14	HB33	AA31G00431	purple
10	αXYL	AA32G01046	turquoise

Supplemental Table S3. RT-qPCR primers targeting *Aethionema arabicum* genes. Primers were designed using Primer3 2.3.4 (Untergasser et al., 2012).

Gene	ID	Direction	Sequence (5´ to 3´)
CTU2†	AA30G00197	Forward	GTTGGCTCGTCCTCCTCAAG
		Reverse	CCGTACAATTGTGCACTCCC
PECT1†	AA255G00007	Forward	AGAAGGAGTTTCCAGCACCG
		Reverse	TGTGAGACTCGAACACTGGC
DAHP2	AA111G00011	Forward	GTGAGCAGGGTGACAGGTAC
		Reverse	CCCAACCCAAAGCATGTGTG
DRE2	AA40G00275	Forward	GAAACCTCAGCTCCCTTCGG
		Reverse	TCCTCGATCTCAGCCCTACC
EXPA9	AA19G00314	Forward	CTGAATGGTGTCTCCCAGGG
		Reverse	CATCTCCTGCTCCAGCTACG
GST	AA3G00170	Forward	CTAAATGGCGCAAGTGGGTG
		Reverse	TCAACCCATGTTTCAGCAGC
LEA14	AA1G00071	Forward	CTGGCCGGGAAATTGCAAAG
		Reverse	ACAACGGGAAGGTCAACGAC
NCED6	AA78G00012	Forward	TCGTACGAGTCGGCTTGTTC
		Reverse	GGAGTGACCGTGTAGCTCAC
RK29	AA4G00252	Forward	TGCTCCGTCTCCAGAAATCG
		Reverse	TCTGCTGCTGCTTCTTCCTC
SGR2	AA6G00349	Forward	GGACCTGCGATATTCGAAGC
		Reverse	TGCCCATCCCTGTAACTGAG
XTH4	AA110G00001	Forward	CAGCCACCGATTTTGCAGAC
		Reverse	TCTCTAAACCGCCACGTGTC
HHO2	AA1304G00001	Forward	TCCTTCTCCGGATTCACAACC
		Reverse	CGTGTCACTTTTAGCACCGC
RAP2.12	AA15G00147	Forward	TCACTTCCACTCCGAAACCC
		Reverse	ATCTCAGCTGCCCATTTCCC
ETR2	AA17G00111	Forward	ATGGGCTGCTTGGAGATCAC
		Reverse	ACGACTGAGATGTTCCCGTG
GBF3	AA21G00361	Forward	TGCTCCTCCTGAAACTTGGC
		Reverse	AGCTCTTCAGTTTCCGCCTG
PDC2	AA215G00009	Forward	TCACTCAGGAGCTTAGGTGC
		Reverse	AGTCGGGTGAGGAATTGCAG
JAZ3	AA26G00461	Forward	GCTTGCCGTCAACATACCAC
		Reverse	TTGTGACCCTTTCTTTGCGC
ABF4***	AA26G00601	Forward	GCAGGCTTACACCTTGGAAC
		Reverse	AGCATTGCCTTTTGTTCCCC
HAB1***	AA31G00661	Forward	TTCGGTGTTCTTGCCATGTC
		Reverse	GCCACACCAAGATCCTCCTC
αXYL1	AA32G01046	Forward	CACTATCCTCCGCCTCTTCG
		Reverse	ACCAGAAATCTCCTGCACGG

GBF5*	AA33G00153	Forward	AAATCTGCTCTCCGCCGTAG
		Reverse	AGCTCCGGTAAGTACTGCTTG
EXPA2	AA35G00022	Forward	GAGGCCACGCTACCTTCTAC
		Reverse	GCAAGCTCCACATGTTGCTC
ABCG40	AA37G00179	Forward	CCTTACTCGGGACCATCGTG
		Reverse	TCTCCATCACTTGCAGGCTC
ADH1a	AA39G00353	Forward	AGAATCTCTGGTGCGTCGAG
		Reverse	CACACTCCTGTCCACTCCAC
NAC102	AA4G00115	Forward	GGAGCTTGATTTACCGGCG
		Reverse	CTTTCTCACCGTATAATGCCAAAT
PYR1/RCAR11	AA57G00057	Forward	ACCGGCGAATACATCCACTG
		Reverse	AACCACCGTCCAGATCTGTG
ABI5	AA60G00170	Forward	TCAAGAACCTACCTTCCCTACAC
		Reverse	CCACCATAACAAACACCGGC
DOG1	AA6G00020	Forward	GGTGGATGCAGACCGTCTTC
		Reverse	AGTTGCTCCGCCGTTAGATC
CYP707A3a*	AA6G00060	Forward	AAAACTCCCTCTTCCTCCGG
		Reverse	GGGTTTTGAACACCGATCCG
XTH16a	AA78G00036	Forward	ACGAGACAGGGAAGCCTTATG
		Reverse	GGACACCAAGATGTTCTGCG
AREB3a	AA93G00015	Forward	AGAATCTGCAGCACGTTCAC
		Reverse	CTGGAGGTGGTGTACTTGGG
HRA1	AA93G00176	Forward	ATGACCGTGGTTCAGGTTCC
		Reverse	TTTCTTCCCGTCGGTTCCTG
ABF3*	AA118G00053	Forward	TGGAAAATCATGCTCCACTTGC
		Reverse	CTTCCGGGAGATACTGCAGC
ERF71/73	AA21G00481	Forward	GTGGGCAAGAGGAGAAGGAG
		Reverse	ACCAAGCCAGAGACGAACAC

†Reference genes used for normalization.
*Annealing temperature 65°C.

Supplemental Table S4. 2-way ANOVA of RT-qPCR analyses presented in Figure 8 and Supplemental Figure S12. P values and significance rating (<0.0001 ****, <0.001 ***, 0.01 **, <0.05 *, >0.05 ns = not significant) are indicated for treatment and time.

Aethionema arabicum **gene**	**M- seed hypoxia** *versus* **M- seed normoxia**		**M- seed ABA/hypoxia** *versus* **M- seed ABA/normoxia**		**IND fruit** *versus* **M- seed normoxia**		**M- seed normoxia** *versus* **M- seed ABA/normoxia**		**M- seed hypoxia** *versus* **M- seed ABA/hypoxia**	
	**Hypoxia**	**Time**	**Hypoxia**	**Time**	**Pericarp**	**Time**	**ABA**	**Time**	**ABA**	**Time**
AearERF71/73	<0.0001 ****	<0.0001 ****	<0.0001 ****	<0.0001 ****	0.0049 **	<0.0001 ****	0.4833 ns	<0.0001 ****	0.0781 ns	<0.0001 ****
AearADH1a	<0.0001 ****	<0.0001 ****	<0.0001 ****	<0.0001 ****	<0.0001 ****	0.3751 ns	0.0466 *	<0.0001 ****	0.0857 ns	<0.0001 ****
AearPDC2	<0.0001 ****	<0.0001 ****	<0.0001 ****	<0.0001 ****	<0.0001 ****	0.0024 **	0.1228 ns	<0.0001 ****	0.9791 ns	<0.0001 ****
AearHRA1	<0.0001 ****	<0.0001 ****	<0.0001 ****	<0.0001 ****	0.2059 ns	0.1666 ns	0.5774 ns	0.6086 ns	0.3470 ns	<0.0001 ****
AearETR2	<0.0001 ****	<0.0001 ****	<0.0001 ****	<0.0001 ****	0.1347 ns	0.0328 *	0.7348 ns	0.2084 ns	0.0047 **	<0.0001 ****
AearJAZ3	0.0014 **	<0.0001 ****	<0.0001 ****	<0.0001 ****	0.0101 *	<0.0001 ****	0.3652 ns	<0.0001 ****	0.2757 ns	0.0043 **
AearDOG1	<0.0001 ****	0.0006 ***	<0.0001 ****	0.0024 **	<0.0001 ****	0.0200 *	0.2546 ns	0.0155 *	0.7241 ns	<0.0001 ****
AearNCED6	<0.0001 ****	0.2729 ns	<0.0001 ****	0.1153 ns	0.0075 **	0.0187 *	0.2160 ns	0.0007 ***	0.2821 ns	0.0016 **
AearCYP707A3a	0.1700 ns	<0.0001 ****	0.6986 ns	<0.0001 ****	<0.0001 ****	<0.0001 ****	0.0428 *	<0.0001 ****	0.4023 ns	<0.0001 ****
AearABCG40	0.0002 ***	0.1377 ns	0.0006 ***	0.0974 ns	0.0319 *	0.5958 ns	0.8296 ns	0.1442 ns	0.8372 ns	0.0029 **
AearAREB3a	<0.0001 ****	0.8502 ns	0.0001 ***	0.7501 ns	0.0064 **	0.0006 ***	0.5384 ns	<0.0001 ****	0.4647 ns	0.0200 *
AearABF4	<0.0001 ****	<0.0001 ****	0.0013 **	<0.0001 ****	0.7507 ns	0.0234 *	0.1887 ns	0.0116 *	0.5542 ns	<0.0001 ****
AearGBF3	<0.0001 ****	0.0009 ***	<0.0001 ****	<0.0001 ****	0.0274 *	0.3455 ns	0.0431 *	0.2008 ns	0.1645 ns	<0.0001 ****
AearHAB1	<0.0001 ****	0.0002 ***	0.0036 **	<0.0001 ****	0.1373 ns	0.0085 **	0.0423 *	0.0123 *	0.8956 ns	<0.0001 ****
AearEXPA2	<0.0001 ****	<0.0001 ****	<0.0001 ****	<0.0001 ****	<0.0001 ****	<0.0001 ****	0.0582 ns	<0.0001 ****	0.1439 ns	0.2364 ns

AearEXPA9	<0.0001 ****	<0.0001 ****	<0.0001 ****	0.0003 ***	<0.0001 ****	<0.0001 ****	0.0065 **	<0.0001 ****	0.0014 **	<0.0001 ****
AearXTH4	<0.0001 ****	<0.0001 ****	<0.0001 ****	0.0002 ***	<0.0001 ****	<0.0001 ****	0.0434 *	<0.0001 ****	0.2153 ns	0.0003 ***
AearXTH16a	<0.0001 ****	<0.0001 ****	<0.0001 ****	<0.0001 ****	<0.0001 ****	<0.0001 ****	0.0017 ns	<0.0001 ****	0.0561 ns	0.0002 ***
AearaXYL1	<0.0001 ****	0.0033 **	<0.0001 ****	0.0002 ***	0.0001 ***	0.0010 **	0.4687 ns	<0.0001 ****	0.4576 ns	0.0831 ns
AearLEA14	<0.0001 ****	0.0366 *	<0.0001 ****	0.0080 **	0.0621 ns	0.0028 **	0.0488 *	0.0122 *	0.0356 *	<0.0001 ****
AearDAHP2	<0.0001 ****	<0.0001 ****	<0.0001 ****	<0.0001 ****	<0.0001 ****	<0.0001 ****	0.7507 ns	<0.0001 ****	0.0164 *	<0.0001 ****
AearGST	0.3703 ns	0.0015 **	0.0022 **	0.0002 ***	<0.0001 ****	0.3631 ns	0.9376 ns	0.0005 ***	0.1178 ns	0.0002 ***
AearNAC102	<0.0001 ****	0.0062 **	<0.0001 ****	0.0017 **	0.2084 ns	0.0011 **	0.2400 ns	<0.0001 ****	0.3277 ns	<0.0001 ****
AearHHO2	0.0016 **	<0.0001 ****	0.0517 ns	<0.0001 ****	0.0002 ***	<0.0001 ****	0.3151 ns	<0.0001 ****	0.3017 ns	0.0001 ***
AearRAP2.12	0.9204 ns	0.0011 **	0.0137 *	0.0007 ***	0.1206 ns	0.0191 *	0.6362 ns	0.0061 **	0.0260 *	<0.0001 ****
AearABI5	0.2944 ns	0.0459 *	0.3405 ns	0.0081 **	0.3051 ns	0.7289 ns	0.0456 *	0.3181 ns	0.4720 ns	<0.0001 ****
AearABF3	0.0031 **	0.5402 ns	0.0457 *	0.1557 ns	0.9786 ns	0.1721 ns	0.3885 ns	0.2669 ns	0.6744 ns	0.0234 *
AearGBF5	0.1957 ns	<0.0001 ****	0.8175 ns	<0.0001 ****	0.0028 **	0.0004 ***	0.3555 ns	<0.0001 ****	0.0178 *	<0.0001 ****
AearPYR1/RCAR11	0.5352 ns	0.0010 ***	0.0106 *	0.0002 ***	0.3721 ns	0.0135 *	0.8969 ns	0.0670 ns	0.0036 **	<0.0001 ****
AearSGR2	0.5242 ns	0.0014 **	0.6165 ns	0.0044 **	0.2945 ns	0.0025 **	0.3554 ns	0.0028 **	0.8572 ns	0.0021 **
AearDRE2	0.0315 *	0.0006 ***	0.3487 ns	<0.0001 ****	0.8865 ns	0.0096 **	0.1848 ns	0.0044 **	0.2321 ns	<0.0001 ****
AearRK29	0.3184 ns	0.1106 ns	0.1325 ns	0.0303 *	0.2511 ns	0.0880 ns	0.8494 ns	0.3435 ns	0.8404 ns	0.0121 *
