# Supplementary material for: The dimorphic diaspore model Aethionema arabicum (Brassicaceae): Distinct molecular and morphological control of responses to parental and germination temperatures
Source: Plant Cell. 2024 Mar 21;36(7):2465–90. doi: 10.1093/plcell/koae085 (PMC11218780; doi:10.1093/plcell/koae085)
Supplement: koae085_Supplementary_Data [file tpc.00654.2023-s07.pdf › tpc.00654.2023-s07.pdf]

THE PLANT CELL

The dimorphic diaspore model Aethionema arabicum (Brassicaceae): Distinct molecular and morphological control of responses to parental and germination temperatures

Jake Chandler, Per Wilhelmsson, Noe Fernandez-Pozo, Kai Graeber, Waheed Arshad, Marta Perez, Tina Steinbrecher, Kristian Ullrich, Thu-Phuong Nguyen, Zsuzsanna Mérai, Klaus Mummenhoff, Günter Theißen, Miroslav Strnad, Ortrun Mittelsten Scheid, M. Eric Schranz, Ivan Petřík, Danuše Tarkowská, Ondřej Novák, Stefan Rensing, and Gerhard Leubner-Metzger

Review Timeline:

Submission Date:	19-Jul-2023
Editorial Decision:	14-Sep-2023
Revision Received:	14-Dec-2023
Editorial Decision:	06-Feb-2024
Revision Received:	21-Feb-2024
Accepted:	23-Feb-2024

Prof. Dr. Gerhard Leubner-Metzger
Royal Holloway University of London
Egham, Surrey TW200EX
United Kingdom

Dear Dr. Leubner-Metzger:

We have received reviews of your manuscript entitled "The dimorphic diaspore model Aethionema arabicum (Brassicaceae): Distinct molecular and morphological control of responses to parental and germination temperatures." Thank you for submitting your best work to The Plant Cell. The editorial board agrees that the work you describe is substantive, falls within the scope of the journal, and may become acceptable for publication, pending revision and potential re-review.

We ask you to pay attention to the following points in preparing your revision: Reviewers and members of the editorial board agreed that the results from the transcriptomics experiments are not conclusive enough to justify some of the papers' conclusions, linking transcriptional changes to phenotypic outcomes. This section of the paper, in particular, should be revised, as suggested by the reviewers, and the language modified to more accurately reflect the uncertaintly of any conclusions that can be drawn from transcriptomic data alone.

Please contact us if there are ambiguous comments or if you wish to discuss the revision.

Given the nature of the comments, we are offering you 60 days from when we have issued this decision to complete the revision. If a revision is not returned within this time frame, and if you have not been granted an extension, we will withdraw the manuscript, which will leave you free to submit the work elsewhere. If you need an extension, we encourage you to contact us at any point before the 60 days have passed.

When you are ready to submit the revised version, please upload a highlighted copy that indicates all changes made in response to the editor and reviewer recommendations. Include an itemized list of all changes made in response to each of the reviewer's suggestions in the "Response to Reviewers" section; please note that reviewers do not have access to your cover letter, nor was this decision letter shared with them.

Thank you for the privilege of reviewing your work. We look forward to receiving your revised manuscript.

Sincerely,

The Plant Cell Editorial Board

Please note the following:

-The Plant Cell now requires authors to complete and submit an author revisions checklist upon submission of a revised manuscript. The aim of the checklist is to aid authors in preparing a high-quality manuscript, facilitate the review and assessment of revised manuscripts, and help to ensure that journal standards are maintained across the board. If your manuscript is accepted, the completed checklist will be published as supplemental material attached to the article online. Please download a copy of the checklist (pdf fillable form) at this link, for submission with your revised manuscript: https://tpc.msubmit.net/html/Author_Revisions_Checklist.pdf.

-Supplemental materials should be restricted to large datasets and tables, presentation of replicates, and validation of reagents, methods, or genotypes. Any data that are used to support the major claims must be in the main manuscript. Supplemental figure legends must indicate what figure in the main manuscript is supported by the supplemental data presented. Please justify how each of the supplemental figures meet the criteria.

-Sampling methods and nature of "biological replicates" should be described precisely (i.e. different plants, parts of plants, pooled tissue, independent pools of tissue, sampled at different times, etc), along with a clear description of and rationale for any statistical analyses conducted. The reader should know exactly what was sampled; what forms the basis of the calculation of any means and statistical parameters reported. This is also necessary to ensure that proper statistical analysis was conducted.

-Want to add this revision deadline to your calendar? Click below!

-- Reviewer comments:

Reviewer #1 (Comments for the Author):

Minor revision. Please see attached document.

Reviewer #2 (Comments for the Author):

This work describes an interesting study which investigates the importance of the maternal tissues of the fruit in the control of seed dormancy, and more precisely the response of seeds to the maternal growth environment. The authors provide strong evidence that environmental sensing by pericarp tissues is important for effects of temperature on progeny seed behaviour, and show therefore that this is at least in part a bona fide maternally derived transgenerational signalling process.

The physiology is well-executed and backed by metabolomic and transcriptomic work which (with some presentational uncertainties, see below) together shows that pericarp ABA plays an important role in the response of seed dormancy to the maternal growth environment. To my knowledge this is a novel and interesting finding.

The work can be improved thus:

1. The location of the ABA is unclear because for the data presented the authors insufficiently clarify whether they show data content of seeds removed from pericarps or seed and pericarp combined in the IND seeds. The text indicates that ABA was quantified in the IND pericarp. Fig 5 Y axis states 'seed ABA content'. Ideally the authors would show both, on a per fruit/seed basis so that it's clear what the relative ABA contents are in each compartment and how these change upon imbibition after the different maternal temperature treatments.

2. The figure panels are referred to out of order in the narrative which then becomes hard to follow. First figure 3A is discussed and then later figures. Only much later does the discussion refer to figures 3B and C. This should be changed.

3. I found that the results section is reduced in quality from line 584 and descends into speculative discussion based on transcriptional changes which clear suggest hypotheses but the authors do not test any of them such that their significance remains unclear. I would either remove these sections, substantially reduce them or ideally improve the manuscript by backing up the work with supporting experiments. For oxygen requirement is a testable hypothesis via manipulation of the partial pressure of O2 during germination. Without this type of work the discussion is too speculative in its current form.

4. Line 422: 'Thus, genes in the green module were induced under conditions unfavorable for germination.'
Is this correct? It looks to me as if these modules contain genes expressed in dry seeds that are to some degree retained in IND seeds but lost upon imbibition in the other samples.

5. Figure 4 'time' is not defined. Imbibition time?

6. Line 332 germinating seed transcriptome is less similar to dry seed than non-germinating seed transcriptome, probably an obvious finding that's not worth a comment.

7. Unless I mis-understand the main conclusion is that pericarp ABA is important for temperature effects on dormancy, but that changes in the seed response to ABA are also required for the full effect. It's unclear whether these arise due to maternal effects during seed maturation of in the seeds themselves or because of some other function of the pericarp. Somehow the abstract could better reflect this.

Reviewer #3 (Comments for the Author):

The authors report a large-scale transcriptomic study focused on the different dispersal strategies of the mustard species Aethionema, which produces two kinds of structures for dispersal - mucilaginous seeds and indehiscent fruits - depending on the environmental conditions. By studying plants grown under different temperatures during

reproductive growth and during germination, the authors identify associations between the dispersal morphs and global gene expression patterns, the levels of several hormones and associated metabolites, and biophysical and morphological properties. Some of the observations, confirming and expanding on a previous study (Lenser et al, 2016), include difference in the ratio of fruit morphs as a result of parental temperature at reproduction (higher temperatures favor the formation of dehiscent fruits) and pericarp-induced dormancy (where seeds still enclosed in the walls of indehiscent fruits exhibit delayed germination compared to both mucilaginous seeds and seeds artificially separated from the fruit). The authors further identify 11 modules of co-expressed genes in a set of 11260 genes using WGCNA, and investigate module gene expression under the different experimental conditions. Case studies aim to clarify the role of the pericarp on ABA metabolism, dormancy and germination and the potential role of oxygen deprivation (or hypoxia-associated gene expression) in the observed phenotypic plasticity.

The initial phenotypic observations are impressive and include thousands of plants grown under different conditions. The transcriptomic data, available as Gene Expression Atlas online, is the central piece of the work (135 sample and experiment accessions submitted to SRA), and is well-curated. I expected to find a table summarizing the overall sequencing and mapping statistics for each library - this is an obvious omission. There are nice, although very preliminary, observations on the biophysical properties of the pericarp. The role of the pericarp in controlling dormancy is interesting, and the results from hormonal levels and expression seem to disprove potential inhibitory role from leakage of ABA or other compounds from the pericarp to the seed.

My main concerns are with the analyses and interpretation of expression data - there are many observed transcriptional changes, which are correlated with phenotypes and conditions but it is unclear if these expression changes have causal effects. Initial RNAseq data is projected after PCA - as currently drawn, the areas on the PC plot (fig. 2) seem a bit arbitrary. How are these circumscribed on the graph? I agree that it *looks* like PC1 reflects germination progress and PC2 presence/absence of pericarp but there are also clear outliers - can this conclusion be tested more formally? Overall, I don't think that describing the PC space warrants that much attention. Furthermore, WGCNA is very coarse (11 co-expressed modules), and I am not convinced that these modules have the biological meaning implied by the authors. The correlations of module expression with some sample traits is deemed statistically significant - what does that mean? Parental temperature was not strongly correlated with any module? This is surprising, could the authors offer any explanation why? In many cases, specific gene(s) from a given module are hand-picked to explain phenotypic differences or environmental effects, with limited downstream validation (although qPCR is provided for some genes). The use of DAPseq data from Arabidopsis in Aethionema is flawed - it assumes that TF binding sites have not changed over more than 40 million years of evolution, which is unjustified. Separate DAPseq dataset with Aethionema TFs has to be generated for the motif analysis.

Detail responses to the reviewer comments:

Reviewer #1 (Comments for the Author):

Minor revision. Please see attached document.

Response: The minor revision requested by this reviewer was fully implemented. Typos were corrected throughout the manuscript.

****

Reviewer #2 (Comments for the Author):

This work describes an interesting study which investigates the importance of the maternal tissues of the fruit in the control of seed dormancy, and more precisely the response of seeds to the maternal growth environment. The authors provide strong evidence that environmental sensing by pericarp tissues is important for effects of temperature on progeny seed behaviour, and show therefore that this is at least in part a bona fide maternally derived transgenerational signalling process.

The physiology is well-executed and backed by metabolomic and transcriptomic work which (with some presentational uncertainties, see below) together shows that pericarp ABA plays an important role in the response of seed dormancy to the maternal growth environment. To my knowledge this is a novel and interesting finding.

Response: Thank you for the positive remarks on the novelty and interest of our findings. We fully addressed the "presentational uncertainties" in the revised manuscript (see below). And we thank this reviewer for proposing the hypoxia experiments which allowed us to experimentally verify the conclusion about the role of hypoxia (see below).

The work can be improved thus:

1. The location of the ABA is unclear because for the data presented the authors insufficiently clarify whether they show data content of seeds removed from pericarps or seed and pericarp combined in the IND seeds. The text indicates that ABA was quantified in the IND pericarp. Fig 5 Y axis states 'seed ABA content'. Ideally the authors would show both, on a per fruit/seed basis so that it's clear what the relative ABA contents are in each compartment and how these change upon imbibition after the different maternal temperature treatments.

Response: We have improved the shortcomings/insufficient clarity in presenting the ABA results. This was achieved by redesign of Figure 6A which now shows ABA and other hormone contents in the IND pericarp (upper panel) and ABA in dry and imbibed dimorphic diaspores in pmol/compartment (per pericarp and per seed and per IND fruit) for in M+ seeds (dry and imbibed seeds), bare M- seeds (dry and imbibed seeds), M- seeds inside IND fruits and separately pericarps only (dry and imbibed IND fruits separated into M- seeds and pericarps at the times indicated), and entire IND fruits (dry and imbibed IND fruits). Figure 5 shows the corresponding ABA in seeds values in pmol/gDW.

Further to this, Supplemental Figure S6A-C shows ABA and other hormone contents during the imbibition at different temperatures. Note that we also used the revision of Supplemental Figure S6A-C to correct a calculation error of the pericarp and fruit hormone metabolite

contents which unfortunately occurred in the original version because we used the wrong published value for the pericarp mass (from Table 1 in Arshad et al 2019 for which we will do an erratum). This is now corrected and we think well presented in the three figures.

We have improved the main text (lines 537-549 paragraph of the original manuscript) for clarity and especially refer to the new Figure 6A and new Supplemental Figure S6C, which provides sufficient clarity about the ABA contents in different compartments.

2. The figure panels are referred to out of order in the narrative which then becomes hard to follow. First figure 3A is discussed and then later figures. Only much later does the discussion refer to figures 3B and C. This should be changed.

Response: To solve this problem, we deleted Figure 3B/C from the new Figure 3 and moved it to the new Supplemental Figure 7 where it is combined with the other "pericarp extract" results. This is also in line with adding the new Figure 8 with the hypoxia results (see next point).

3. I found that the results section is reduced in quality from line 584 and descends into speculative discussion based on transcriptional changes which clear suggest hypotheses but the authors do not test any of them such that their significance remains unclear. I would either remove these sections, substantially reduce them or ideally improve the manuscript by backing up the work with supporting experiments. For oxygen requirement is a testable hypothesis via manipulation of the partial pressure of O2 during germination. Without this type of work the discussion is too speculative in its current form.

Response: We thank the reviewer for proposing the additional hypoxia experiments to test the hypothesis that the pericarp (fruit coat) confers hypoxia to the encased M^- seed inside IND fruits, leading to inhibition/delay of germination, we imbibed bare M^- seeds (extracted from IND fruits by pericarp removal) in hypoxia (4.5% oxygen) and compared the expression of 32 transcriptome-derived DEGs to normoxia (21% oxygen, control). The results from this additional experiments revealed that, in almost all cases, the IND/pericarp-regulated gene expression patterns in the RNAseq data were also observed in RT-qPCR analysis with bare M^- seeds imbibed in hypoxia, which were also delayed in germination.

We therefore revised this section (page 19-23) of the manuscript by deleting more speculative parts and replacing it with the results from the hypoxia experiments presented in the new Figure 8 and Supplemental Figure S13. We conclude that hypoxia can indeed mimick the influence of the pericarp for gene regulation of the encased M^- seed inside IND fruits and delay in germination.

4. Line 422: 'Thus, genes in the green module were induced under conditions unfavorable for germination.'
Is this correct? It looks to me as if these modules contain genes expressed in dry seeds that are to some degree retained in IND seeds but lost upon imbibition in the other samples.

Response: We have deleted this sentence, it is not needed.

5. Figure 4 'time' is not defined. Imbibition time?

Response: We have corrected this by replacing "Time" with "Imbibition time".

6. Line 332 germinating seed transcriptome is less similar to dry seed than non-germinating seed transcriptome, probably an obvious finding that's not worth a comment.

Response: We have reworded and shortened this sentence.

7. Unless I mis-understand the main conclusion is that pericarp ABA is important for temperature effects on dormancy, but that changes in the seed response to ABA are also required for the full effect. It's unclear whether these arise due to maternal effects during seed maturation of in the seeds themselves or because of some other function of the pericarp. Somehow the abstract could better reflect this.

Response: To consider this we have added to the abstract "…identified a role of the IND pericarp in imposing coat dormancy by generating hypoxia affecting ABA sensitivity." and "Parental temperature affected ABA contents and ABA-related gene expression and altered IND pericarp biomechanical properties ".

****

Reviewer #3 (Comments for the Author):

The authors report a large-scale transcriptomic study focused on the different dispersal strategies of the mustard species Aethionema, which produces two kinds of structures for dispersal - mucilaginous seeds and indehiscent fruits - depending on the environmental conditions. By studying plants grown under different temperatures during reproductive growth and during germination, the authors identify associations between the dispersal morphs and global gene expression patterns, the levels of several hormones and associated metabolites, and biophysical and morphological properties. Some of the observations, confirming and expanding on a previous study (Lenser et al, 2016), include difference in the ratio of fruit morphs as a result of parental temperature at reproduction (higher temperatures favor the formation of dehiscent fruits) and pericarp-induced dormancy (where seeds still enclosed in the walls of indehiscent fruits exhibit delayed germination compared to both mucilaginous seeds and seeds artificially separated from the fruit). The authors further identify 11 modules of co-expressed genes in a set of 11260 genes using WGCNA, and investigate module gene expression under the different experimental conditions. Case studies aim to clarify the role of the pericarp on ABA metabolism, dormancy and germination and the potential role of oxygen deprivation (or hypoxia-associated gene expression) in the observed phenotypic plasticity.

Response: Thank you for this summary which reflects our work very well. We have extended the revised manuscript by adding hypoxia experiments, confirming the role of changing the "potential role of oxygen deprivation (or hypoxia-associated gene expression) in the observed phenotypic plasticity. " (Please see response to point 3 of reviewer #2).

The initial phenotypic observations are impressive and include thousands of plants grown under different conditions. The transcriptomic data, available as Gene Expression Atlas online, is the central piece of the work (135 sample and experiment accessions submitted to SRA), and is well-curated. I expected to find a table summarizing the overall sequencing and mapping statistics for each library - this is an obvious omission.

Response: The table summarizing the overall sequencing and mapping statistics for each library is now available as part of Supplemental Data Set 1. This is now also stated explicitly in the materials and methods section.

There are nice, although very preliminary, observations on the biophysical properties of the pericarp. The role of the pericarp in controlling dormancy is interesting, and the results from hormonal levels and expression seem to disprove potential inhibitory role from leakage of ABA or other compounds from the pericarp to the seed.

Response: This part has been improved in the revised manuscript by presenting the ABA results better (see point 1 of reviewer #2) and by additional experimental results from hypoxia experiments (see responses to point 3 of reviewer #2).

My main concerns are with the analyses and interpretation of expression data - there are many observed transcriptional changes, which are correlated with phenotypes and conditions but it is unclear if these expression changes have causal effects. Initial RNAseq data is projected after PCA - as currently drawn, the areas on the PC plot (fig. 2) seem a bit arbitrary. How are these circumscribed on the graph? I agree that it "looks" like PC1 reflects germination progress and PC2 presence/absence of pericarp but there are also clear outliers - can this conclusion be tested more formally? Overall, I don't think that describing the PC space warrants that much attention.

Response: We have modified and shortened this part (see point 6 of reviewer #2). We cannot think of a more formal way to draw the areas in the PCA plot. This is just for better visualisation helping the reader, but can be removed if wanted by the publisher.

Furthermore, WGCNA is very coarse (11 co-expressed modules), and I am not convinced that these modules have the biological meaning implied by the authors. The correlations of module expression with some sample traits is deemed statistically significant - what does that mean? Parental temperature was not strongly correlated with any module? This is surprising, could the authors offer any explanation why?

Response: The correlation analysis clearly identifies modules associated with, for example, "imbibition time", "imbibition temperature", "ABA", "GR50%", "Gmax", "IND fruit", "M- seed" and more. In our opinion, this justifies the number of 11 modules as a good choice. In addition, increasing the number of modules would reduce the number of genes assigned to each module potentially precluding meaningful statistically significant enrichment of Gene Ontologies (Figure 4, Supplemental Data Set 2). Correlations of expression of module genes with trait analyses is a commonly employed method to associate trait data with identified modules. Statistical significance was based on the Pearson correlation coefficient and the number of data points. This provides us with a reasonable level of confidence that the correlation between module gene expression and trait data is not due to chance. Indeed, it seems that "parental temperature" is not strongly correlated by itself, to any trait, which may be due to the much stronger effects of the "imbibition time", "imbibition temperature", "ABA", "GR50%", "Gmax", "IND fruit", "M- seed" per se on module gene expression. Indeed, we observe that differences in gene expression of diaspores are more evident only at the two lower temperatures, where morph-specific germination differences are also more evident. Further, it seems that specific expression patterns, for example for ABA-related genes (Figure 5) mainly differ in the extent of the response between the two parental temperatures, but not in the general expression pattern.

In many cases, specific gene(s) from a given module are hand-picked to explain phenotypic differences or environmental effects, with limited downstream validation (although qPCR is provided for some genes).

Response: This has changed in the revised version by the additional hypoxia RT-qPCR experiments with 32 genes (new Figure 6 and Supplemental Figure S13). These additional experiments validated the expression patterns of these genes in almost all cases. For the effect of the pericarp compared to the bare M- seeds for example, NAC102 and ABI5 are exceptions (Supplemental Figure S13). On the other hand, validation of the effect of hypoxia on the expression patterns of the 32 genes was confirmed (Figure 8; exception CYP707A3). We conclude that these RT-qPCR experiments support that hypoxia can indeed mimic the proposed role of the pericarp in gene regulation through conferring hypoxia to the encased M^- seed inside IND fruits thereby delaying germination.

The use of DAPseq data from Arabidopsis in Aethionema is flawed - it assumes that TF binding sites have not changed over more than 40 million years of evolution, which is unjustified. Separate DAPseq dataset with Aethionema TFs has to be generated for the motif analysis.

Response: Generating separate DAPseq dataset with Aethionema TFs for the motif analysis is not possible in the timeframe of a revision. In our opinion, this is also not needed because we focus our analysis on very general motifs which are widely conserved among multiple species. We have revised this section of the manuscript (page 19-23) by deleting more speculative parts and replacing it with the results from the hypoxia experiments presented in the new Figure 8 and Supplemental Figure S13 (see point 3 of reviewer 2). We now refer to Figure 7C as a "hypothethical working model". While this is true for the details of the hypoxia and ABA regulation (their promoter motifs), this is not any more the case for the hypoxia and ABA regulation of the 32 genes for which we did RT-qPCR experiments (see previous point). From these expression patterns (Figure 8 and Supplemental Figure S13), hypoxia seems to be upstream of ABA, which identified a role of the IND pericarp in imposing coat dormancy by generating hypoxia affecting ABA sensitivity.

Dear Dr. Gerhard Leubner-Metzger:

We have received reviews of your manuscript entitled "The dimorphic diaspore model Aethionema arabicum (Brassicaceae): Distinct molecular and morphological control of responses to parental and germination temperatures." On the basis of the advice received, the board of reviewing editors would like to accept your manuscript for publication in The Plant Cell. This acceptance is contingent on revision based on the comments of our reviewers. In particular, please consider revising the text to deal with the reviewer's comments. We don't think any additional experiments are necessary, but do think the reviewer's point are important, and can be addressed by softening language and adding explanation in the text.

Please highlight all changes and include a detailed annotation of changes of the text, with line numbers, and noting your responses to the comments.

To submit your revised manuscript, click:

Link Not Available

If you have any questions about the revision submission procedures, please contact the Editorial Office Staff (tpc-submissions@aspb.org). If you cannot return the revised manuscript within 30 days, please let us know. Otherwise, we will assume that you have elected not to revise the manuscript and withdraw it.

Thank you very much for the privilege of reviewing this work. I look forward to receiving the next version.

Sincerely

The Plant Cell Editorial Board

Reviewer #3 (Comments for the Author):

Thank you for considering my suggestions - the library statistics are great, the supplementary datasets are curated beautifully, and I appreciate the addition of seed coat experiments, which strengthen original arguments. I have two comments, which are related to the interpretation of the data and not to the value of the dataset:

1. The coarseness of the WGCNA analysis - as the authors correctly argue in their response, reducing the number of genes assigned to a module will affect the significance of GO enrichment. The problem is that the more genes are included in a set, the more likely it is to get GO term enrichment by chance. The current analysis includes more than 11000 expressed genes spread out across 11 modules (average 1000 genes/module - a vast number demanding the question whether this is a biologically meaningful entity that would inform subsequent work - I don't think it is in this context). No doubt that correlation of gene expression and traits to identify modules of coexpressed genes (based on Pearson correlation) is useful - what is deemed significant here and how that relates to the trait under consideration?
2. I am skeptical about using motif (and binding site) information from Arabidopsis in Aethionema - overall conservation of cis-regulatory elements in Brassicaceae aside, there is no actual evidence that ERF7X binding is conserved. It is difficult, without rigorous testing, to judge the conservation of cis-regulation among species, particularly phylogenetically divergent ones exhibiting divergent traits like Arabidopsis and Aethionema. These analyses are not only hypothetical but also misleading - they leave the impression that such comparisons are straightforward and we do not need to study many different species to discover relevant biology, something the authors would agree is not justified.

* throughout the paper: PRINCIPAL component analysis (not principle component analysis)

Please also note the following:

-The Plant Cell now requires authors to complete and submit an author revisions checklist upon submission of a revised manuscript. The aim of the checklist is to aid authors in preparing a high-quality manuscript, facilitate the review and assessment of revised manuscripts, and help to ensure that journal standards are maintained across the board. If your manuscript is accepted, the completed checklist will be published as supplemental material attached to the article online. Please

download a copy of the checklist (pdf fillable form) at this link, for submission with your revised manuscript: https://tpc.msubmit.net/html/Author_Revisions_Checklist.pdf.

-Supplemental materials should be restricted to large datasets and tables, presentation of replicates, and validation of reagents, methods, or genotypes. Any data that are used to support the major claims must be in the main manuscript. Supplemental figure legends must indicate what figure in the main manuscript is supported by the supplemental data presented. Please justify how each of the supplemental figures meet the criteria.

-Sampling methods and the nature of "biological replicates" should be described precisely (i.e. different plants, parts of plants, pooled tissue, independent pools of tissue, sampled at different times, etc.), along with a clear description of and rationale for any statistical analyses conducted. The reader should know exactly what was sampled; what forms the basis of the calculation of any means and statistical parameters reported. This is also necessary to ensure that proper statistical analysis was conducted.

Detail responses to the editorial and reviewer comments:

Editor decision letter

"We have received reviews of your manuscript entitled "The dimorphic diaspore model Aethionema arabicum (Brassicaceae): Distinct molecular and morphological control of responses to parental and germination temperatures." On the basis of the advice received, the **board of reviewing editors would like to accept your manuscript for publication in The Plant Cell.** This acceptance is contingent on revision based on the comments of our reviewers. In particular, please consider revising the text to deal with the reviewer's comments. **We don't think any additional experiments are necessary, but do think the reviewer's point are important, and can be addressed by softening language and adding explanation in the text.**"

Response: Thank you for the positive response. We also realised that original reviewer #1 and #2 did not make further comments and assume they were fully satisfied with our first revision. For the second revision which addresses two remaining points of reviewer #3, we have addressed these by improving the parts of the manuscript, including by "softening language and adding explanation in the text" as proposed by the board of reviewing editors.

Reviewer #3 (Comments for the Author):

"Thank you for considering my suggestions - the library statistics are great, the supplementary datasets are curated beautifully, and I appreciate the addition of seed coat experiments, which strengthen original arguments. I have two comments, which are related to the interpretation of the data and not to the value of the dataset:"

Response: We thank the reviewer for the appreciation of our revision 1 in which we already addressed most of the criticism.

"1. The **coarseness of the WGCNA analysis** - as the authors correctly argue in their response, reducing the number of genes assigned to a module will affect the significance of GO enrichment. The problem is that the more genes are included in a set, the more likely it is to get GO term enrichment by chance. The current analysis includes more than 11000 expressed genes spread out across 11 modules (average 1000 genes/module - a vast number demanding the question whether this is a biologically meaningful entity that would inform subsequent work - I don't think it is in this context). No doubt that correlation of gene expression and traits to identify modules of coexpressed genes (based on Pearson correlation) is useful - what is deemed significant here and how that relates to the trait under consideration?"

Response: In our opinion, the critisised "coarseness of the WGCNA analysis" (11 modules each with between 259 and 2213 genes) does not seem to generate the problem claimed by reviewer #3, it in fact led to identifying "meaningful" key biological processes as already exemplified in the correlation analysis to traits (Figure 4). Even the largest module (turquoise with 2213 genes) delivered a comprehensive set of cell-wall remodelling genes (Figure 10 and Supplemental Figure S16), all these genes were identified as enriched in the analysis and correlated with "germination-promotion".
Whether or not a larger number of modules each with fewer genes would have led to further "biologically meaningful" process identifications has not been tested, simply

because the 11 modules led to the successful identification of the genes discussed in our article. We also do not claim that that there are no further "biologically meaningful" genes. However, the ones identified with our 11 modules reveal that the approach was successful because they are representing "biologically meaningful processes". To address the criticism we have added explanatory text to the section "Co-expressed gene modules in dry and imbibed seed transcriptomes associated with morph-specific germination responses".

2. **I am skeptical about using motif (and binding site) information from Arabidopsis in Aethionema** - overall conservation of cis-regulatory elements in Brassicaceae aside, there is **no actual evidence that ERF7X binding is conserved**. It is difficult, without rigorous testing, to judge the **conservation of cis-regulation among species**, particularly phylogenetically divergent ones exhibiting divergent traits like Arabidopsis and Aethionema. **These analyses are not only hypothetical but also misleading** - they leave the impression that such comparisons are straightforward and we do not need to study many different species to discover relevant biology, something the authors would agree is not justified.

Response: To address these points we have improved the entire section "Evidence for pericarp-mediated hypoxia, morph-specific transcription factor expression, and ABA signaling in the control of *Ae. arabicum* dormancy and germination". This was by softening language, by adding explanation in the text, and by a complete re-arrangement of the section which now focuses on the hard facts first, and has the hypothetical working model more towards the end with explicit emphasis that it requires experimental validation in future research.

The revised section now starts (**first paragraph**) with added explanation about the current state of cross-species knowledge for TFs and cis-regulatory elements across species. The additional references support this as we of course wanted to keep this part brief (omitted most of the rice ERF VII literature). We also highlight the evolved difference between *Ae. arabicum* and *A. thaliana* in their ERF71/73 and ADH genes.

Several ERF group VII TFs have also been identified in kiwifruit (*Actinidia* spp.) and their involvement in triggering ADH and PDC gene expression upon waterlogging stress (hypoxia) was demonstrated (Bai et al., 2021; Liu et al., 2022). We also emphasize recent work by **Bai et al. (2024) who demonstrated that overexpression of AvERF73 in *A. thaliana* and *Actinidia chinensis* (kiwifruit) enhances waterlogging tolerance, demonstrating that *cis*-elements targeted by ERF group VIIs controlling response to hypoxia are similar across the core Eudicots (Rosids and Astrids).**

The **second paragraph** describes the observed expression patterns of ADH/PDC and other *Ae. arabicum* homologs for the *A. thaliana* core list of hypoxia-responsive genes (Gasch et al 2016). The **third paragraph** provides the experimental evidence for their regulation by hypoxia in the RT-qPCR experiments with M^- seeds where hypoxia mimics the pericarp of IND fruits. The **fourth and fifth paragraph** is now the in silico analysis (chord diagram and FIMO). While the HRPE and ABRE motifs are more widely conserved, we refer to the ERF73 motif explicitly as "putative ERF73 motif" to consider the criticism*. The derived model is explicitly referred to as "hypothetical working model" in the main text and the figure. We have also added explanation that the "hypothetical working model" requires experimental validation in future research.

* Bai et al. (2024) recently demonstrated that overexpression of AvERF73 in *A. thaliana* and *Actinidia chinensis* enhances waterlogging tolerance including enrichment in GO term "cellular response to hypoxia". This work demonstrated that the ERF73 *cis*-elements of these two distantly related species are similar and DAP-seq of *A. chinensis* identified a core GCCGCC binding motif which is typical for ERFs (Bai et al., 2024). There is therefore evidence that ERF7X binding is widely conserved and cis-acting elements are similar.

Reviewer #3 seems to imply that at least some cis-elements are conserved, and it is our view that the fantastic resources of the model plant Arabidopsis should be used to generate working models. These have some value as confirmed by numerous publications in the field, especially if they are applied to related Brassicaceae species including *Aethionema*. Based on the work of Bai et al. (2024) this is even more justified also for ERF73

* throughout the paper: PRINCIPAL component analysis (not principle component analysis)

Response: The typo occurred once the Figure 2 legend and once in the Figure S3 legend and was corrected.

Dear Dr. Gerhard Leubner-Metzger:

We are pleased to inform you that your paper entitled "The dimorphic diaspore model Aethionema arabicum (Brassicaceae): Distinct molecular and morphological control of responses to parental and germination temperatures" has been accepted for publication in The Plant Cell, pending a final editorial review by a science editor. At this stage, your manuscript will be evaluated by a science editor with respect to its presentation of scientific content, compliance with journal policies, and presentation for a broad readership. The Plant Cell has contracted with Plant Editors (planteditors.com) to provide this service to our authors, and you will soon receive additional information on this process.

Please note that each author needs to link their ORCID identifier to their account in the system before your manuscript can be published. If any authors do not have an ORCID linked to their account, they will receive a message with a link to complete this task. Please ensure that ALL of your coauthors have completed this task as soon as possible.

ASPB offers an OPEN option that allows authors to have their online articles available for free to all users immediately upon publication. For more information about the ASPB OPEN option, refer to the Final Submission Checklist Form.

The Plant Cell and The Arabidopsis Information Resource (TAIR) are collaborating to collect functional annotation data about Arabidopsis genes from authors. This includes information about the gene's molecular function (e.g., kinase activity, ATP synthetase activity), the biological process/es it is involved in (e.g., endosperm development, threonine biosynthesis), its subcellular location (e.g., nucleus, ER), anatomical or developmental expression pattern (e.g., leaf, ovule, flower stage 10, seedling stage), or its partner in a protein-protein interaction (e.g., AT1G01010 interacts with AT1G01020).

If your paper contains results falling into one or more of these categories for Arabidopsis genes, we request that you now submit these data for inclusion in TAIR by filling in the form provided at the following URL: https://www.arabidopsis.org/doc/submit/functional_annotation/123. If you need further clarification on what types of data can be submitted please contact curator@arabidopsis.org.

Finally, we encourage your submission of artwork for the journal cover. Monthly issues will have an online cover image and selected covers will be used for posters, other promotional items, and "wallpaper" for mobile devices. For more information, refer to Cover Submission in the Instructions for Authors https://tpc.msubmit.net/cgi-bin/main.plex?form_type=display_auth_instructions.

We look forward to seeing your paper published.

Sincerely,

The Plant Cell Editorial Board

=====
IMPORTANT REMINDER: PEER REVIEW REPORTS
=====

If you opted to publish a peer review report along with your article during the original submission process, it will be prepared by the editorial staff and publicly posted with your manuscript, inside the zip file that contains any other supplemental material. As a reminder, the peer review report is a public record of all comments from editors and reviewers, as well as your prior responses, as you received them in the decision letters for each draft of your manuscript. If you agreed to publish this report and have changed your mind, or are not sure if you selected this option, please contact the editorial office as soon as possible before signing the license agreement from our publisher.

=====

----FOR ASPB OFFICE USE ONLY (DO NOT EDIT)----
MSID: 35298
Corresponding Author: Prof. Dr. Leubner-Metzger
